# Gathering Evidence to Leverage Musculoskeletal Magnetic Stimulation Towards Clinical Applicability

**DOI:** 10.1002/smsc.202300303

**Published:** 2024-02-26

**Authors:** José G. S. Figueiredo, Bárbara M. de Sousa, Marco P. Soares dos Santos, Sandra I. Vieira

**Affiliations:** ^1^ Department of Medical Sciences Institute of Biomedicine (iBiMED) University of Aveiro 3810‐193 Aveiro Portugal; ^2^ Department of Mechanical Engineering Centre for Mechanical Technology & Automation (TEMA) University of Aveiro 3810‐193 Aveiro Portugal; ^3^ LASI ‐ Intelligent Systems Associated Laboratory Guimarães 4800‐058 Portugal

**Keywords:** animal models, clinical studies, combined magnentic fields (CMF), inductive coupling, musculoskeletal disorders, pre‐clinical studies, pulsed electromagnetic fields (PEMF)

## Abstract

Musculoskeletal disorders are among the main causes of disease‐associated disability. Moreover, the incidence and prevalence of osteoporosis and osteoarthritis, as well as the risk of bone fractures and the need for joint replacements, are expected to increase with longer life expectancy. New approaches based on electromagnetic stimulation have been developed, aiming to shorten bone healing time, attenuate osteoporosis and osteoarthritis, and increase implants' osseointegration. Inductive coupling (IC), a non‐invasive methodology to deliver magnetic stimuli, has reached clinical trials and some clinical practices but is not yet considered a standard procedure. Indeed, its feasibility in clinical use is still under discussion, and optimal stimulation parameters are fairly undefined. This comprehensive review describes the research trends and applicability of IC‐based therapeutics for musculoskeletal disorders, and starts identifying top‐performing magnetic stimulation parameters. Insights into the magnetic stimuli setups that promote osteogenesis are provided, based on pre‐clinical and clinical evidence from 117 in vivo studies in animal models and human patients. Potential cellular and molecular biomechanisms mediating IC‐induced effects on osteoblasts and osteoclasts are also explored. The transversal knowledge herein delivered will hopefully support innovative designs and medical devices that will implement IC stimulation as a clinical standard and effective therapeutic for musculoskeletal disorders.

## Graphical abstract

Inductive coupling (IC)‐induced magnetic stimulation is here reviewed as a safe and promising non‐invasive therapy to manage musculoskeletal disorders, such as osteoporosis, osteoarthritis, and implant osseointegration. Gathered data, based on the trends, operating parameters and outcomes of 117 IC pre‐clinical and clinical studies, will aid on designing clinical studies aimed to infer on optimal stimuli parameters of higher success rates.

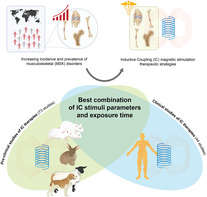

## 1 Introduction

The 2019 Global Burden of Disease placed musculoskeletal disorders among the 10 top drivers of increasing burden, given its increasing impact on disability‐adjusted life‐years (DALYs) from 1990 to 2019.^[^ ^1^ ^]^ Accordingly, these disorders were also leading causes of years lived with disability (YLDs) during that period.^[^ ^2^ ^]^ It is thus imperative that health systems rapidly address the needs of individuals affected by musculoskeletal conditions endangering functional health.^[^ ^3^, ^4^ ^]^ Surgery is indicated for severe musculoskeletal disorders that cause substantial pain and disability, and for symptoms refractory to conservative treatment.^[^ ^5^ ^]^ Total hip and knee arthroplasties are among the most common and successful surgical procedures, and are projected to grow around 71% and 85% by 2030 in the United States.^[^ ^6^ ^]^ Despite continuous improvements in surgical techniques and implant designs, revision arthroplasties are still rising due to unsuccessful osseointegration.^[^ ^7^, ^8^ ^]^ Innovative therapies for musculoskeletal disorders, and strategies to improve the efficacy of ongoing ones, are therefore an urgent healthcare priority.^[^ ^4^ ^]^

Active bone healing, key for long‐term successful therapies, may be achieved by stimulating bone tissue remodeling via biochemical, mechanical, or electromagnetic stimuli.^[^ ^9^ ^]^ The benefits of exogenously delivered electromagnetic stimuli have been recognized for pain relief, wound healing, treatment of neuromuscular dysfunctions, pharmaceuticals’ delivery (iontophoresis), among others.^[^ ^10^, ^11^, ^12^, ^13^ ^]^ Bone and other musculoskeletal tissues may also benefit from these, given their responsiveness to extracorporeal electromagnetic stimuli that mimic natural endogenous bone electromagnetic fields and induce similar biological effects.^[^ ^14^, ^15^ ^]^ Indeed, bone composition confers specific electromechanical properties to this tissue, activated by mechanical loading through compression or tension.^[^ ^16^, ^17^ ^]^ Two hypotheses account for the electrical potentials generated in loaded bone: the piezoelectric effect (mainly on dry bone, and attributed to the crystalline micelle of collagen molecules)^[^ ^16^, ^18^, ^19^ ^]^ and the streaming potentials (that explain the smaller potentials on hydrated bone, which in vivo few piezoelectric responses given its water content).^[^ ^17^, ^20^ ^]^ Following mechanical loading, the bioelectrical signals in wet bone are attributed to the electrokinetic behavior of the ionic movement after changes in spatial charge density caused by bone bending or extracellular fluid movement in the bone matrix.^[^ ^21^, ^22^, ^23^, ^24^ ^]^ Bone macrostructure (cortical or trabecular) and intrinsic factors, like age, mineral, and organic content, induce additional variations in bone dielectric properties.^[^ ^25^, ^26^, ^27^, ^28^, ^29^ ^]^

Many researchers have explored the effects of electromagnetic fields on the tightly orchestrated bone remodeling process.^[^ ^30^, ^31^, ^32^ ^]^ Several animal models have been used to test the efficacy of electromagnetic stimulation in preventing and even reversing osteoporosis, a major musculoskeletal disorder characterized by low bone mass and structural deterioration due to imbalanced bone remodeling.^[^ ^33^, ^34^ ^]^ Electromagnetic stimulation is also valuable for bone regeneration upon fracture, promoting bone repair and fracture healing.^[^ ^35^, ^36^ ^]^ Electromagnetic stimulation may ultimately improve osseointegration, crucial for the long‐term survival of implantable orthopedic devices.^[^ ^8^, ^37^, ^38^ ^]^ Current bone‐implant interfaces cannot ensure a timeless stable performance, mainly when interfacing osteoporotic bones,^[^ ^39^ ^]^ leading to loss of primary stability and early implant loosening, and contributing to the increased incidence of revision arthroplasties.^[^ ^40^, ^41^, ^42^ ^]^ Multifunctional smart implants, acting on bone‐implant interfaces and with the ability to deliver personalized electromagnetic stimuli, may ultimately control bone regrowth and optimize osseointegration, thus reducing the incidence of revision surgeries.^[^ ^37^, ^43^, ^44^, ^45^ ^]^

Three methods have been used to provide electromagnetic stimulation, which includes the delivery of electrical fields (EF) and magnetic fields (MF). Direct electric current (DC) stimulation, requiring direct contact of the cathode with injured bone tissues or bone cell cultures used as research models, has been widely studied for bone healing.^[^ ^46^, ^47^, ^48^ ^]^ However, the invasiveness of DC stimulators is inherently associated with infections, inflammation, soft tissue discomfort, or cathode rupture.^[^ ^47^ ^]^ Contrariwise, non‐invasive stimulation through EF and/or MF has significant advantages, including the absence of toxic chemicals’ formation and immune responses in host tissues, besides requiring simpler designs and involving little tissue handling.^[^ ^49^ ^]^ Further, devices incorporating non‐invasive stimulators allow both repeated treatment and monitoring of target regions.^[^ ^37^, ^43^, ^44^, ^49^ ^]^ The Food and Drug Administration (FDA) has already approved non‐invasive bone growth stimulators acting through the other two methods: capacitive coupling (CC) and inductive coupling (IC).^[^ ^50^ ^]^ Classic CC stimulation architectures require two electrodes placed on opposite sides of the target tissue to deliver sinusoidal EFs, while IC stimulation is mostly achieved through the delivery of pulsed electromagnetic fields (PEMF) or combined magnetic fields (CMF), provided by a device surrounding the body target region.^[^ ^50^ ^]^ PEMF stimulation involves the flow of alternating electric currents through solenoids or coils, generating MFs that in turn induce electric voltages on bone tissues.^[^ ^47^, ^48^ ^]^ These induced voltages depend on the MF strengths and the magnetic properties of the target tissues. CMF stimulation combines these coil‐generated PEMF with an overlapping static MF provided by permanent magnets.^[^ ^47^ ^]^ Both CC and IC methods have been proposed as therapeutic strategies to minimize osteoporosis, promote bone fracture healing, and improve osseointegration on bone‐implant interfaces.^[^ ^51^ ^]^ Notwithstanding the wide number of studies on IC applications for bone conditions, it is now crucial to optimize the stimulation elicited by IC in clinical settings by identifying the IC operating parameters that deliver the best bone healing outcomes. Other reviews have addressed the efficacy of IC stimulation on bone disorders, but 1) for specific conditions, like bone fractures,^[^ ^52^ ^]^ osteoporosis,^[^ ^53^ ^]^ osteoarthritis and joint disabilities;^[^ ^54^, ^55^ ^]^ or 2) limited to PEMF's clinical applications.^[^ ^56^, ^57^, ^58^ ^]^

We here comprehensively overview the most relevant bone healing‐related outcomes induced by IC in vivo, both in animal models and human patients, aiming to identify top‐performer magnetic stimulation parameters that induce significant improvements in musculoskeletal conditions. A search was carried out on PubMed to find studies published until the end of 2023, focused on the delivery of magnetic stimulation via IC in vivo, in both animal models and humans, to treat bone detrimental conditions, such as fractures or osteoporosis, or to promote bone‐implant osseointegration. Relevant parameters were extracted from the retrieved studies, including the type of stimulation, stimulation device, waveform, frequency, periodicity, MF strength, daily exposure time, and assay duration. Magnetic stimuli parameters were uniformized according to the standardized signal characterization (Figure S1, Supporting Information). Data relative to the animal models, alongside the clinical conditions evaluated and supplementary procedures or treatments, were also collected. Focus was placed on the most frequently assessed biological/clinical outcomes: radiographic assessments of bone unions, micro‐CT scan analyses of bone microarchitecture, histological analyses of the healing tissue, final biomechanical properties, and osteogenesis‐related molecular markers. Data on these biological outcomes were extracted from the articles’ text, tables or embedded figures. Unless otherwise specified, indicated changes (incremental or decremental %, or fold‐changes) refer to stimulated versus unstimulated conditions.

## 2 Pre‐Clinical Studies of IC Therapies

### 2.1 Overview of IC Pre‐Clinical Designs

**Figure 1:**
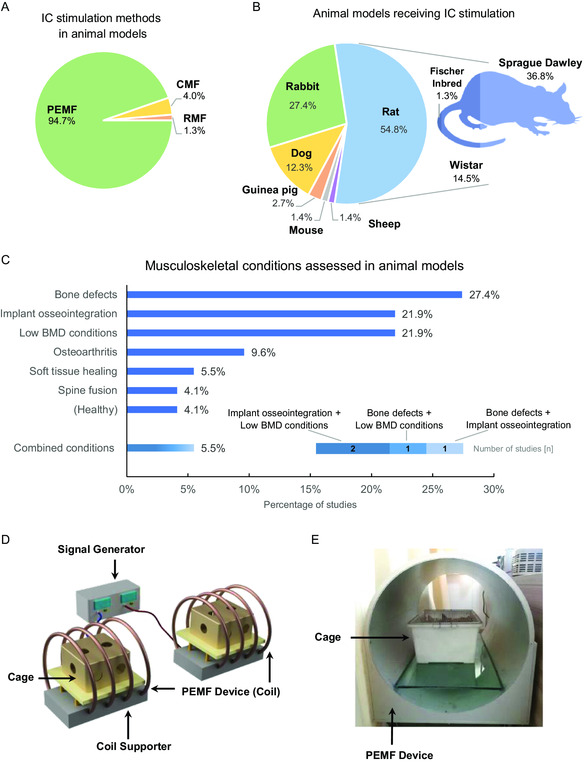
General trends of IC stimulation on pre‐clinical studies with animal models of musculoskeletal disorders. A) IC stimulation methods applied; B) Type of animal models used; C) Musculoskeletal conditions assessed in the animal studies retrieved (details in Table S1, Supporting Information). ”Bone defects” include osteotomies, fractures, and bone drills; “Low BMD conditions” are conditions resulting in low bone mineral density (BMD) and bone fragility, such as osteoporosis, diabetes, and glucocorticoid treatments; “Combined conditions” represent 4 studies (out of the 73) in which 2 conditions were simultaneously assessed. D) Illustrative schematic of a typical IC stimulation device used in pre‐clinical studies to deliver PEMF to animals. Based on illustrations from Jing et al.^[^ ^120^ ^]^ and Cai et al.^[^ ^87^ ^]^ E) Photograph of an actual IC stimulation device used in a pre‐clinical study here reviewed. Reproduced and adapted with permission.^[^ ^102^ ^]^ Copyright 2018, Wiley. CEF: constant electromagnetic field; CMF: combined magnetic field; PEMF: pulsed electromagnetic field; RMF: rotating magnetic field.

Our search has retrieved 73 pre‐clinical studies using IC stimulators to treat musculoskeletal conditions in animal models (Table S1, Supporting Information). PEMF was by far the most used IC stimulation method (94.7%) and various waveforms (sinusoidal, square, triangular, and sawtooth) have been tested (**Figure** **1**A and Table S1, Supporting Information). As presented in Figure 1B, rats (54.8%; mainly Sprague Dawley, but also Wistar and Fischer Inbred) were the most used animals to test IC stimulation, followed by rabbits (27.4%). Other animal models used were dogs (Beagle and mixed breed), guinea pigs (New Zealand White and Japanese White), mice, and sheep.

The pre‐clinical therapeutical efficacy of IC stimulation has been tested for several musculoskeletal conditions, namely bone defects, implants’ osseointegration, conditions of low bone mineral density (BMD), soft tissue healing, spine fusion, and osteoarthritis, as well as in healthy subjects. Bone defects^[^ ^59^, ^60^, ^61^, ^62^, ^63^, ^64^, ^65^, ^66^, ^67^, ^68^, ^69^, ^70^, ^71^, ^72^, ^73^, ^74^, ^75^, ^76^, ^77^, ^78^, ^79^, ^80^, ^81^ ^]^ were the most assessed condition (in 27.4% of the studies; Figure 1C and Table S1, Supporting Information). IC stimulation has also been widely used to assess implants’ osseointegration (21.9%),^[^ ^70^, ^82^, ^83^, ^84^, ^85^, ^86^, ^87^, ^88^, ^89^, ^90^, ^91^, ^92^, ^93^, ^94^, ^95^, ^96^, ^97^, ^98^, ^99^ ^]^ which was usually tested on rabbits.^[^ ^83^, ^86^, ^87^, ^88^, ^91^, ^92^, ^93^, ^94^, ^95^, ^96^, ^97^, ^98^, ^99^, ^100^, ^101^ ^]^ A typical IC stimulation device applied in pre‐clinical studies to deliver PEMF is schematically presented in Figure 1D. Additionally, Figure 1E portrays a photograph of an actual IC stimulation device used in a pre‐clinical study here reviewed.^[^ ^102^ ^]^

**Figure 2:**
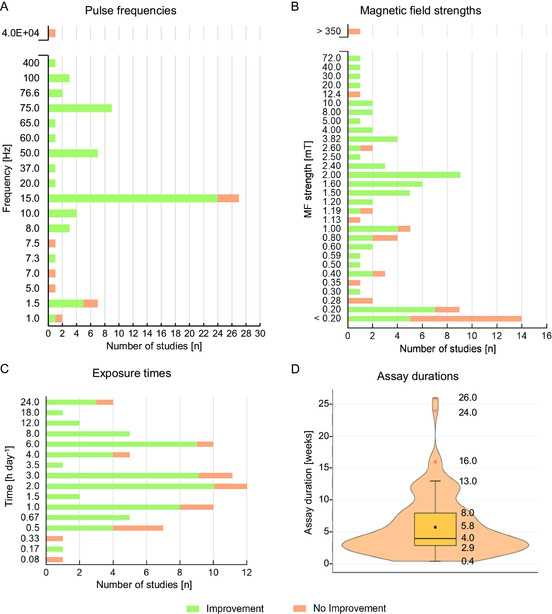
IC stimuli parameters applied in animal pre‐clinical studies. Graphical analysis of the different IC stimulation parameters used in the 73 animal studies reviewed, plotting the number of studies applying such parameters, and the stimulation's relative success (details in Table S1 and S2, Supporting Information). A) Pulse frequencies used, in Hz; of note, 5 studies had non‐defined pulse frequencies and 6 studies tested 2 different pulse frequencies, totalizing the 75 pulse frequencies here presented. B) Magnetic field (MF) strengths applied, in mT; 10 studies had non‐defined MF strengths, while 6, 3, and 1 studies tested 2, 3, and 4 different MF strengths, respectively, and 1 study tested 14 different MF strengths, totalizing the 91 MF strengths presented. C) Daily exposure time to stimulation, in hours day^−1^; 3 studies had non‐defined exposure times, while 4 and 2 studies tested 2 and 3 different exposure times, respectively, totalizing the 78 different exposure times presented. Studies reporting positive outcomes upon IC stimulation are colored green, and studies reporting no effect or detrimental outcomes are colored orange. D) Box‐violin plot with the distribution and summary statistics of the total duration of the IC stimulation assay, in weeks; 12 and 7 studies tested 2 and 3 different durations, respectively, while 1 study tested 4 different durations and another tested 5 different durations, totalizing the 106 different assay durations presented.

The tested pulse frequencies ranged from 1 Hz^[^ ^73^ ^]^–400 Hz,^[^ ^66^ ^]^ with the most tested frequency being 15 Hz (in 36.5% of the tested frequencies, **Figure** **2**A). Unless stated otherwise, all frequencies here indicated refer to pulses (that comprise sets of bursts), and not to individual bursts (see Figure S1, Supporting Information, for the distinction between pulses and bursts). MF strengths (Figure 2B) ranged from 0.02 mT^[^ ^69^ ^]^–600 mT,^[^ ^103^ ^]^ but around three‐fourths (74.7%) of the tested set ups used MF strengths up to 2.0 mT. Noteworthy, most studies reporting no improvements have overall used low frequencies (<8 Hz) and MF strengths (<1.2 mT). Animals were exposed to IC stimulation from 5 min day^−1^ up to a full day, but exposure times were tendentially between 1–3 h day^−1^ (in 44.9% of studies, Figure 2C). IC stimulation assays lasted 6 weeks on average (Figure 2D).

Regarding the biological outcomes, these may differ due to anatomical and physiological differences between the animal models. For comparative purposes, the biological outcomes were analyzed by groups of animals categorized according to their size, as follows: 1) small‐sized animals (rats, mice, and guinea pigs); 2) intermediate‐sized animals (rabbits); and 3) medium‐sized animals (dogs and sheep).

### 2.2 Effects of IC Stimulation on Small‐Sized Animals

Rats (*Rattus norvegicus*) were the most used animal models to test IC stimulation in vivo (Figure 1). Rats are anatomic and physiologically well‐studied, have fast growth and reproductive rates and require little aftercare, being appropriate to study various stimulation parameters at reasonable costs. In contrast, their quite smaller size, different proportions of cortical and trabecular bone, innate bone growth after sexual maturity, and higher healing capabilities, may result in different responses from humans upon similar stimuli.^[^ ^104^ ^]^ IC stimulation has been used to improve different musculoskeletal conditions in these animals, as detailed in the sections below, divided by types of musculoskeletal conditions, and in Table S1 and S2, Supporting Information.

PEMF have been applied to determine whether bone tissues from healthy rats responded well to electromagnetic stimuli. These studies were focused on major macroscopical and microarchitectural effects on bone and also spotlighted some molecular markers and signaling mechanisms altered upon PEMF stimulation under physiological conditions. PEMF of 1 Hz and 30 mT, delivered by the FDA‐approved transcranial stimulator Magstim 220 for 30 min day^−1^ over 3 weeks, has increased cortical and trabecular bone thickness by nearly +50%, in all measurements.^[^ ^105^ ^]^ To understand the PEMF's action mechanism on healthy bones, a study applied PEMF of 50 Hz, 0.6 mT, 1.5 h day^−1^ over 3 months, and measured molecular markers related to bone homeostasis and to the sAC/cAMP/PKA/CREB signaling pathway. PEMF stimulation increased BMD (≈+10%) and bone volume (1.69‐fold change), as well as the mechanical properties (up to +14.3% maximum load), and microarchitecture of bone: increased trabecular thickness (Tb.Th, +39.0%) and number (Tb.N, +21.2%), and reduced trabecular separation (Tb.S, −40.5%). Moreover, this therapy significantly increased serum levels of the bone formation marker procollagen type I N‐terminal propeptide (P1NP, +15.0%), and tendentially reduced the bone resorption marker C‐terminal cross‐linked telopeptides of type I collagen (CTX‐I, −19.1%). Increased expression levels of femur p‐PKA and p‐CREB, soluble adenyl cyclase (sAC), and serum cAMP were also observed, indicating osteogenic effects through the sAC/cAMP/PKA/CREB signaling pathway.^[^ ^102^ ^]^

#### 2.2.1 Managing Bone Defects in Small‐Sized Animals Through IC Stimulation

PEMF of rather different parameters were applied to heal rat bone defects (fractures and osteotomies) and consolidate bone fusions, with promising effects such as faster osseous bridging, assessed by osteogenic markers and other readouts.^[^ ^60^, ^63^, ^66^, ^69^, ^77^, ^78^, ^80^, ^81^, ^106^ ^]^ PEMF of 15 Hz quasi‐rectangular pulses and 0.15–0.18 mT MF strength, applied for 12 h day^−1^ over 5 weeks to bone defects of rats’ premaxilla, were able to increase by 2‐fold the alkaline phosphatase (ALP) activity from day 7 (D7) to D14, and Ca^2+^ incorporation at D14 by 3‐fold.^[^ ^60^ ^]^ PEMF of the same frequency and higher MF strengths also ameliorated other rats’ bone defects.^[^ ^80^, ^81^ ^]^ Daily distracted tibial osteotomies stimulated with 15 Hz, 1.2 mT, 3 h day^−1^ for 4 weeks, improved bone volume (+34.9%), mineral density (+19.9%), cartilage mineralization (+31.9%), and bone strength (ultimate load +171%, energy to failure +235.7%, elastic modulus +95.3%).^[^ ^81^ ^]^ At a molecular level, these stimuli additionally increased tissue levels of collagen‐I (1.38‐fold) and osteocalcin (OC, 1.71‐fold).^[^ ^81^ ^]^ IC stimulation has been tested on other bone defects, and femur incisions treated with PEMF of 15 Hz and 5 or 10 mT, for 2 h day^−1^ during a week, exhibited improvements in several biological outcomes evaluated at D21 after therapy onset. These included BMD (+19.6% and +16.9%, for 5 and 10 mT, respectively), serum ALP (+22.6%/+12.1%), serum Ca^2+^ (+15.0%/+2.8%), fracture load (+19.4%/+18.0%), and bending energy (+15.9%/+18.5%).^[^ ^80^ ^]^

Studies using PEMF of higher pulse frequencies, from 20 to 400 Hz, have mostly reported positive impacts on bone healing (Figure 2). For example, PEMF of 50 Hz and 1.5 mT, applied to rat tibial fractures for 3.5 h day^−1^ during 3 weeks, increased osteogenic histological markers and serum OC (+36.8%).^[^ ^65^ ^]^ The same PEMF parameters applied to rat femur fractures for 6 h day^−1^ over 4 weeks, also improved histopathological scores at D21 and D30 (≈+20.0%).^[^ ^77^ ^]^ Rat calvaria defects stimulated with similar PEMF (60 Hz, 1.0 mT) for 8 h day^−1^ over 5 days, outperformed the control group by presenting increased bone volume (+350.2%) and microarchitecture (+153.2% Tb.Th; +71.2% Tb.N; −20.0% Tb.S).^[^ ^78^ ^]^ Serum markers of osteogenesis (osteopontin, OPN) and angiogenesis (von Willebrand factor, vWF) were also upregulated.^[^ ^78^ ^]^ Rats’ femur fractures stimulated with PEMF of 400 Hz pulses (bursts of 27 MHz) for 10 min day^−1^ over 2 weeks, had increased circulating ALP (+21.7%) and OC (+21.5%) levels, more bone volume in the callus (1.14‐fold) with better microarchitecture (+18.4% Tb.Th; −16.5% Tb.S), which led to mechanically stronger bones (+60.8% strength; −32.0% elastic deformation).^[^ ^66^ ^]^

An FDA‐approved PEMF device, Physio‐Stim, delivering pulses at the most commonly used frequency (15 Hz pulse), of triangular or sawtooth bursts at 3.85 kHz, and 2.0 mT with a daily exposure of 3 h day^−1^, has improved healing of rat fibular osteotomies.^[^ ^63^, ^69^ ^]^ One study applied such stimulation parameters over 3 and 5 weeks, and reported higher osteotomy gap filling and a 2‐fold increase in hard callus formation rate and volume, and in bending strength.^[^ ^69^ ^]^ Another study applied this stimulation over 10 weeks and observed reduced bone volume loss by 0.31‐fold.^[^ ^63^ ^]^ Stimuli delivered by other FDA‐approved devices, like Osteo‐Stim (1.5 Hz, 0.02 mT, 3 h day^−1^)^[^ ^69^ ^]^ and PAP IMI (5 Hz, 4 min day^−1^),^[^ ^61^ ^]^ were not as effective for osteotomies, with unstimulated bones of the control group healing faster,^[^ ^61^ ^]^ or higher fibrous content being found in PEMF‐stimulated bones.^[^ ^61^, ^69^ ^]^ This loss of effectiveness may potentially be ascribed to their lower frequencies and generated MF.

#### 2.2.2 Studies of IC Stimulation for Soft Tissue Disorders in Small‐Sized Animals

Physio‐Stim was also used for soft tissue healing in rats^[^ ^107^, ^108^, ^109^ ^]^ and PEMF of parameters similar to the ones described above (15 Hz; ≈2 mT) led to radiographical, biomechanical, and histological improvements in teared rotator cuffs.^[^ ^107^, ^108^ ^]^ When delivered for 1 h day^−1^ over 4 weeks, it increased the tendon modulus and stiffness and enhanced collagen‐I and fibronectin tendon expression.^[^ ^108^ ^]^ When delivered for 3 h day^−1^, such stimuli increased the tendon modulus and maximum stress at 4 weeks, and improved bone quality at 16 weeks.^[^ ^107^ ^]^ However, another study applying the same stimuli to teared Achilles’ tendons, for 1 or 3 h day^−1^ over 1, 3, or 6 weeks, did not observe major improvements in bone volume or BMD, and even reported impaired tendon mechanical properties (e.g., reduced stiffness and modulus at 3 weeks).^[^ ^109^ ^]^

#### 2.2.3 Tackling Osteoporosis in Small‐Sized Animals Through IC Stimulation

Osteoporosis, a highly relevant musculoskeletal disorder, is usually studied in animal models by mimicking osteoporotic phenotypes of low BMD, either through ovariectomy (OVX; the most used osteoporosis model),^[^ ^110^ ^]^ disuse (hindlimb unloading (HU) which is a model that avoids weight‐bearing by the animal's hindquarters)^[^ ^103^, ^111^ ^]^ or drug induction (e.g., heparin treatment).^[^ ^112^ ^]^ In two studies, stimulation of OVX rats with PEMF of 8 Hz, 3.82 mT, for 40 min day^−1^ over 12 weeks prevented bone microarchitecture and biomechanical deterioration.^[^ ^113^, ^114^ ^]^ Vertebral bodies stimulated with those PEMF demonstrated increased BMD and bone volume up to +32.78% and 1.23‐fold, respectively, and improved microarchitecture: up to +14.5% Tb.Th, +20.3% Tb.N, and −16.8% Tb.S.^[^ ^113^, ^114^ ^]^ These stimuli also reduced serum tartrate‐resistant acid phosphatase 5b (TRACP5b, −26.1%), and mRNA levels of receptor activator of NF‐κB ligand (RANKL, −41.0%), while increased by 3.8‐fold osteoprotegerin (OPG) mRNA expression in vertebral bodies, femurs, and tibias.^[^ ^113^ ^]^ These molecular alterations are usually related to reduced osteoclast differentiation/activation, decreased bone resorption and increased bone deposition.^[^ ^113^ ^]^ Mechanically, PEMF‐stimulated vertebral bodies and femurs were stronger, with increased load‐bearing, energy to failure, and stiffness (up to +15.3%, +30.9%, +11.7%, respectively).^[^ ^113^, ^114^ ^]^ Importantly, applying this PEMF treatment immediately post‐OVX yielded better results than if the treatment was initiated 12 weeks post‐OVX, highlighting that the timing of PEMF therapy initiation is important to achieve optimal effects.^[^ ^114^ ^]^ Another study treating OVX rats with PEMF of higher pulse frequency (15 Hz lineal waveform pulses) but lower MF (0.5 mT), for 3 h day^−1^ over 6 weeks, observed faster bridging of fibular fractures (+17.4%), improved repair (+11.7%), and callus elastic modulus (+68.8%), and no alterations in bone volume.^[^ ^64^ ^]^ Other studies using PEMF of equal frequency but higher MF strength, have also improved bone preservation in OVX rats. PEMF of 15 Hz, 1 mT, applied to rats with periodontitis (with and without OVX) for 3 h day^−1^ over 3 weeks, has (in both cases) attenuated the effects of bone loss (+27.8% BMD), increased bone volume‐to‐total volume ratio (BV/TV + 28.6%), improved bone microarchitecture (+11.5% Tb.N, −8.0% Tb.S) and reduced markers of inflammatory response such as IL‐1β, IL‐6, TNF‐α, IL‐10 and vascular endothelial growth factor (VEGF).^[^ ^115^ ^]^ PEMF of 15 Hz, 2.4 mT, for 8 h day^−1^ over 10 weeks, improved bone biomechanical properties (+15.2% maximum load; +46.6% elastic modulus), reduced bone loss (+41.8% BMD; +1.83‐fold BV/TV), enhanced bone microarchitecture (+54.7% Tb.N, +17.6% Tb.Th, −18.6% Tb.S) and reduced structure model index (SMI, −31.7%).^[^ ^116^ ^]^ OVX‐induced bone loss was also avoided when OVX mice were treated with similar PEMF: 15 Hz of square wave pulses and 2.4–2.6 mT MF strength, for 1 h day^−1^ over 8 weeks.^[^ ^117^ ^]^ PEMF of 15 Hz and MF strengths of either 0.41, 1.2, 4.1, or 12.4 mT, applied for 3 h day^−1^ over 6 weeks, overall improved trabecular bone formation rate, relatively to sham controls, untreated OVX and rats treated with alendronate, a drug to reduce osteoporosis‐related bone loss and fracture risk.^[^ ^118^ ^]^ The 12.4 mT‐treatment induced the highest Tb.N increase and Tb.S decrease, while outcomes like osteoclast number, canalicular length, lacunae number, and size were more sensitive to PEMF of lower MF strengths.^[^ ^118^ ^]^ PEMF of 1.2 mT ameliorated trabecular BMD and bone mineral content (BMC) loss (by 0.63‐fold) almost as efficiently as alendronate.^[^ ^118^ ^]^

For disuse osteoporosis models, PEMF of 10 Hz and 3.82 mT, for 40 min day^−1^ over 12 weeks, improved bone microarchitecture (increased Tb.Area and Tb.N; reduced Tb.S) and increased serum OC by +14.8% in HU rats.^[^ ^119^ ^]^ Improvements in various biological outcomes were also obtained by increasing PEMF frequency and daily exposure time, while decreasing MF strength and total assay duration. For example, disuse‐induced decrease of bone mass and deterioration of trabecular and cortical bone microarchitectures were attenuated by such a PEMF stimulation (15 Hz, 2.4 mT, 2 h day^−1^ over 4 weeks), which also increased mineral apposition and bone formation rates (both ≈+119%) and bone mechanical properties.^[^ ^120^ ^]^ In terms of potential molecular and cellular action mechanisms, this IC stimulus significantly promoted osteoblastogenesis (+53.3%) and increased serum levels of bone formation markers (+47.0% OC; +59.2% P1NP), while decreasing bone resorption ones (−16.4% CTX‐I; −15.2% TRACP5b). Altered expression of genes related to Wnt/β‐catenin signaling indicates that this pathway can promote some improvements in animals with osteoporosis.^[^ ^120^ ^]^ PEMF of similar parameters (15 Hz; 2 h day^−1^) but even lower MF strength (0.8 mT), applied over 8 weeks, also attenuated BMD loss in the proximal femur of HU rats (+32.7% than non‐stimulated HU animals), and acted on bone remodeling pathways: +42.1% serum TGF‐β1 and −21.7% serum IL‐6.^[^ ^111^ ^]^ PEMF of higher frequency (50 Hz) and lower MF strength (0.6 mT), applied for 1.5 h day^−1^ over 4 weeks, also considerably prevented HU‐induced loss of BMD (by ≈50%) and of maximum loads in femurs and vertebral bodies, while preserving cortical and trabecular bone microarchitectures.^[^ ^121^ ^]^ Again, besides inducing these radiological and biomechanical outcomes, PEMF increased bone formation markers (+137.5% OC; +39.0% P1NP) and reduced bone resorption ones (−18.8% CTX‐I; −35.3% TRACP5b).^[^ ^121^ ^]^ At a molecular signaling level, these PEMF reverted the usual HU‐induced decreases of parathyroid hormone and its downstream signaling molecule cAMP (+56.4% and +106.0% than non‐stimulated HU animals), sustaining the PEMF‐induced activation of the sAC/cAMP/PKA/CREB pathway, in an overall attempt to prevent bone loss.^[^ ^121^ ^]^ Contrarily, rotating magnetic fields (RMFs) of lower frequencies and much higher MF strengths (7 Hz, 380–600 mT), applied for 2 h day^−1^ over 4 weeks, were ineffective in preserving bone quality in HU rats.^[^ ^103^ ^]^

In cases of drug‐induced osteoporosis, PEMF of 7.3 Hz and 0.8 mT for 1 h day^−1^ over 4 weeks reverted heparin‐induced bone loss, increasing new bone area by 4‐fold and reducing serum CTX (−20.7%).^[^ ^112^ ^]^ PEMF of higher frequency and MF strength (50 Hz, 4.0 mT) for 40 min day^−1^ over 12 weeks prevented bone adverse effects of glucocorticoid‐induced osteoporosis by avoiding BMD loss and improving bone health serum markers (+58.3% ALP; ‐10.6% TRACP5b).^[^ ^122^ ^]^ Molecularly, PEMF acted through the Wnt/β‐catenin pathway (increased Wnt10b, LRP5, β‐catenin; decreased Dkk‐1, Axin2, SOST).^[^ ^122^ ^]^

Finally, in senile osteoporosis models, PEMF of 8 Hz and 3.82 mT (applied for 40 min day^−1^) partially reverted aging‐related bone deterioration, after 12 weeks of stimulation. Improved BMD, bone microarchitecture, and serum bone markers (+78.1% ALP; −28.0% TRACP5b) were reported, and again the Wnt/β‐catenin signaling was suggested as a mediator of these improvements, given the upregulation of genes related to this pathway (Wnt3a, LRP5, β‐catenin), and the Wnt target gene Runx2 (a transcriptional modulator of osteoblasts differentiation, that upregulates OC), and downregulation of the Wnt antagonist PPAR‐γ.^[^ ^123^ ^]^

#### 2.2.4 IC Stimulation to Improve Implant Osseointegration in Small‐Sized Animals

IC stimulation was also tested on rat implants’ osseointegration. A study applying PEMF with the most common parameters (15 Hz, 1.0 mT), has compared the effectiveness of 1 vs 3 h day^−1^ exposures, for 5 days week^−1^ over 45 days, in promoting titanium tibial implants’ osseointegration.^[^ ^85^ ^]^ Both exposures increased Tb.N by ≈50% but, although 3 h day^−1^ exposure preserved more Tb.Th around implants (−55.0% vs. −37.2%), 1 h day^−1^ exposure generally induced better results: increased bone volume (4.7‐fold), BMD (+54.6%) and bone‐implant contact (BIC, 2.2‐fold), better removal torque tests (+47%), enhanced cell viability, protein content, and nodules’ mineralization.^[^ ^85^ ^]^ In a relevant study that placed a titanium implant in the rat's right tibial crest, and performed an osteotomy in the contralateral tibial crest, increased peri‐implant and osteotomy ossification were observed in the group stimulated for 0.5 h twice‐a‐day with PEMF of 50 Hz and high MF strength (72 mT), demonstrating that this IC therapy can accelerate both bone‐healing and peri‐implant bone formation.^[^ ^70^ ^]^ PEMF of higher frequency and lower MF strength (75 Hz, 2.5 mT), delivered by Biostim for 6 h day^−1^ over 60 days, also improved osseointegration of intramedullary titanium pins in rat femurs. Namely, increased bone volume (1.52‐fold), BIC (2.40‐fold), and microhardness (+19.4%) were detected, as well as reduced cortical width (Ct.Wi, −32.3%), fibrous capsule formation (capsule thickness, −52.8%) and osteoclast number (−72.9%).^[^ ^90^ ^]^ Inspired by a study on rabbits’ femur implants,^[^ ^82^ ^]^ PEMF stimulation of 100 Hz and 0.2 mT was successfully applied to titanium tibial implants in OVX rats for 4 h day^−1^ over 2 weeks, resulting in higher peri‐implant bone volume (up to 1.75‐fold) and increased Tb.S (up to +31.5%).^[^ ^84^ ^]^

#### 2.2.5 Handling Osteoarthritis in Small‐Sized Animals with IC Stimulation

In two studies of a rat model mimicking temporomandibular joint osteoarthritis, PEMF of 2 mT and 15 Hz pulses (bursts of 5 kHz), applied for 2 h day^−1^ over 3 or 6 weeks, partially reversed microarchitecture deterioration, as shown by increased BV/TV (up to 1.4‐fold) and Tb.Th (up to +36.8%); reduced bone surface (BS/BV, 0.83‐fold) and Tb.Sp (up to −29%)^[^ ^124^ ^]^ and reversed the decrease in cartilage thickness (by ≈20.6% on average).^[^ ^125^ ^]^ This IC stimulation also reversed the upregulated osteoclast activity and related RANKL gene expression, and the abnormal downregulation of osteogenic factors (OPG, ALP, Runx2, and OC), that commonly occur at early osteoarthritis stages.^[^ ^124^ ^]^ Additionally, these IC stimulation parameters inhibited the up‐regulation of pro‐inflammatory and degradative proteins (TNF‐α, IL‐1β, MMP‐13, ADAMTS‐5, IL‐6, MMP‐3, MMP‐9, and COL‐X) in the synovium, that classically characterize osteoarthritis.^[^ ^125^ ^]^ To induce knee osteoarthritis, rats were administered low‐dose monosodium iodoacetate and further treated for 2 h day^−1^ over 4 weeks with PEMF of higher frequency (75 Hz; 1.6 mT). Improved bone volume (1.18‐fold) and microarchitecture (+18.5% Tb.Th, +6.8% Tb.N, −13.6% Tb.S) of osteoarthritic knees were recorded, as well as increased serum levels of osteogenic markers (e.g., +47% OC), and decreased bone and cartilage resorption ones (−7.8% CTX‐I; −19.9% CTX‐II).^[^ ^126^ ^]^ A follow‐up study using the same model and stimulus reported similar radiological findings (increased BV/TV, Tb.Th, Tb.N; decreased Tb.S), and correlated these with increased OPG/RANKL ratio (+87.0%) and Wnt/β‐catenin pathway activation (increased Wnt3a, LRP5, β‐catenin).^[^ ^127^ ^]^

Other animals used as osteoarthritis models are guinea pigs (*Cavia porcellus*) of the Dunkin‐Hartley breed, that spontaneously develop degenerative joint diseases around 3 months old.^[^ ^104^ ^]^ Improved cartilage thickness (+15.2%) and lower subchondral bone thickness (SBT, −14.7%) were detected in guinea pigs receiving the same PEMF (75 Hz, 1.6 mT), for 6 h day^−1^ over 13 weeks for knee osteoarthritis, although with no significant differences in epiphyseal trabecular bone remodeling.^[^ ^128^ ^]^ Another study stimulated guinea pigs in late knee osteoarthritis stages with 1.5 mT PEMF for 6 h day^−1^ over 12 weeks and tested two pulse frequencies: 37 and 75 Hz. Both frequencies lowered Fibrillation Index (FI) and SBT, with the 75 Hz being more beneficial to cartilage (+20.6% thickness; −18.5% cartilage FI). Nevertheless, both frequencies were detrimental for microarchitecture (averages of −21.2% Tb.N and +26.5% Tb.S).^[^ ^129^ ^]^

### 2.3 Effects of IC Stimulation on Intermediate‐Sized Animals

Rabbits (*Oryctolagus cuniculus*) are the second usual choice to study IC stimulation on bone remodeling and implants’ osseointegration (Figure 1). Comparatively to smaller models, rabbits undergo more secondary bone remodeling and have sufficient cortical and cancellous bone, and satisfactory intramedullary space to test various types of bone implants. Compared to larger animals, rabbits reach skeletal maturity earlier, and have more fatty bone marrow and thinner condylar cartilage than humans, besides faster cortical bone remodeling and joint cartilage healing rates at young ages.^[^ ^104^ ^]^

#### 2.3.1 Managing Bone Defects in Intermediate‐Sized Animals Using IC Stimulation

Various studies investigated the IC stimulation impact on callus and bone formation in rabbits’ bone defects (fractures/osteotomies).^[^ ^67^, ^68^, ^71^, ^72^, ^76^, ^79^ ^]^ Daily distracted tibial osteotomies, exposed to PEMF of 1.5 Hz for 1 h day^−1^, up to 23 days, generated more callus at D9‐D16, improved fracture bridging at D21, decreased gaps at D9‐D23, leading to increased torque (+52.3%) and stiffness (+31.3%) as early as D9, that reached levels of intact bone by D16.^[^ ^71^ ^]^ A study testing different MF strengths on rabbits’ fibular osteotomies concluded that both PEMF of 0.141 and 0.395 mT (at 15 Hz) significantly increased callus bending stiffness when applied 24 h day^−1^ over 16 days.^[^ ^72^ ^]^ A comparative study assessing rabbit tibial osteotomies stimulated with either PEMF (15 Hz, 1.6 mT) or CMFs (76.6 Hz sine wave of 20 mT, superimposed with a 20 mT static MF) by 0.5, 3 or 6 h day^−1^ for 2–3 weeks, reported increased periosteal callus area at D14 by ≈10% and 30% upon 3 and 6 h day^−1^ exposures, respectively. The stimulation method and daily exposure influenced the amplitude of the effects on bone stiffness and torque, being the highest for PEMF applied for 6 h day^−1^ (+46.7% and +69.9%, respectively; against +30.6% and +55.5% for CMFs, although not statistically different).^[^ ^67^ ^]^ Another study has compared PEMF (20 Hz, 10 mT) and CMFs (20 Hz, 40 ± 5 mT, applied via an intramedullary implant), delivered 1 h day^−1^ over 5 weeks, to treat femoral condylar defects. Although both stimuli promoted new bone formation in the medullar cavity, intramedullary‐applied CMFs performed better, inducing hypertrophic chondrocytes and higher osteogenic effects than PEMF, including new bone volume (+1.86‐fold vs. +1.43‐fold for PEMF) and microarchitecture (Tb.N: +25.0% vs. +9.4%; Tb.S: −20.0% vs. −5.0%).^[^ ^68^ ^]^ Regarding higher frequencies, rabbits with lateral knee arthrotomies (joint incision) filled with a collagenous scaffold and stimulated by PEMF (75 Hz, 1.5 mT) for 4 h day^−1^ over 6 weeks, had improved cartilage morphology, less hypocellularity, and better bone microarchitecture parameters, when compared to the unstimulated scaffold group.^[^ ^79^ ^]^

#### 2.3.2 IC Stimulation Effects on Implant Osseointegration in Intermediate‐Sized Animals

Bone replacement of rabbits’ hindlimbs was commonly used to test IC‐induced implant osseointegration. Matsumoto et al.^[^ ^82^ ^]^ applied 100 Hz PEMF to improve osseointegration of dental implants in rabbits’ femurs and tested two exposure times (4 and 8 h day^−1^), and three MF strengths (0.2, 0.3, and 0.8 mT), over 1, 2, or 4 weeks. Both exposure times increased BIC and bone area over control, without significant differences between them. BIC and bone area also increased with all MF strengths tested but were higher for 0.2 mT (+1.92‐fold BIC; +1.70‐fold area) and 0.3 mT (+1.9‐fold BIC; +1.55‐fold area) than for 0.8 mT (+1.34‐fold BIC; +1.03‐fold area). Bone formation around the implant was higher at 2 weeks than at 1 week, but not significantly different from bone formation at 4 weeks.^[^ ^82^ ^]^ Similar PEMF stimulation (100 Hz, 0.2 mT, 4 h day^−1^) applied over 2 weeks on titanium dental implants placed in the mandible, significantly increased osteoblast number and trabecular bone upon 6 weeks post‐treatment.^[^ ^96^ ^]^

Another study using PEMF of similar MF strengths but at 1/10 of the pulse frequency and with increased exposure (10 Hz, 0.2–0.4 mT, 24 h day^−1^) for up to 4 weeks, also improved osseointegration of implants in rabbits’ tibial metaphyses.^[^ ^86^ ^]^ No gaps or connective tissue were observed on the bone‐implant interface, and bone microarchitecture has improved, specifically bone volume (+62.5%), Tb.N (+35.5%), Tb.S (−29.1%), BIC (+1.30‐fold), and connectivity density (+43.1%).^[^ ^86^ ^]^ Intramedullary Kirshner wires implanted in rabbits’ humerus stimulated with identical PEMF (10 Hz, 0.2 mT, 12 h day^−1^) for 2 weeks, also exhibited increased bone formation around the implant (+128.2%), and higher ALP (+190.0%) and osteoblast proliferative (+62.5%) activities in the bone marrow.^[^ ^76^ ^]^ Another study using stationary or movable femoral and tibial intramedullary Kirshner wires, stimulated 4 h day^−1^ with PEMF of 15 Hz pulses of trapezoidal bursts over 3 weeks, concluded that stimulated movable femoral implants had a stronger repair response than unstimulated movable ones (+1.44‐fold new bone formation; +21.5% enlargement of the medullary canal area).^[^ ^92^ ^]^ These increases were even higher when compared to stationary femoral implants (either stimulated or not), suggesting that PEMF effects can be synergistically enhanced when combined with other stimuli.^[^ ^92^ ^]^ However, these differential effects were not observed for tibial implants, potentially derived from the femur's higher vascularity, cellularity and healing capacity, or even to its increased susceptibility to electromagnetic fields.^[^ ^92^ ^]^ Stimulating femoral porous titanium implants with PEMF of 2.0 mT and similar 15 Hz pulses, for lower daily exposures (2 h day^−1^) but longer assay durations (6 and 12 weeks), also improved bone microarchitecture around implants (−39.9% BS/BV, +83.3% BV/TV, +19.0% Tb.N, +45.7% Tb.T, −36.8% Tb.S) and increased OC (+120.5%) and Runx2 (+104%) levels, potentially via the osteogenic Wnt/β‐catenin pathway (Wnt1 + 230%; Lrp6 + 279%; β‐catenin +490%).^[^ ^98^ ^]^

In a study comparing tibial implants, PEMF of 1.5 Hz (quasi‐square pulses), 0.18 mT, applied 8 h day^−1^ for 6 weeks, accelerated bone formation and maturation (thicker trabeculae; most significantly upon 3–4 weeks of stimulation) around hydroxyapatite (HA) but not around tricalcium phosphate (TCP) implants.^[^ ^91^ ^]^ Moreover, HA tibial implants stimulated with PEMF of higher pulse frequency and MF strength (50 Hz, 8 mT), for 0.5 h twice‐a‐day over 4 weeks, presented earlier integration and faster bone formation around implants than unstimulated controls.^[^ ^94^ ^]^ Accelerated osseointegration and increased mineral apposition rate (+66.4%^[^ ^97^ ^]^), bone contact (affinity index: +1.47‐fold^[^ ^83^ ^]^ and +1.11‐fold^[^ ^97^ ^]^) and trabecular microhardness (+49.3%^[^ ^83^ ^]^ and +36.0%^[^ ^97^ ^]^) were observed around femoral HA implants stimulated with PEMF of 75 Hz, 1.6 mT, for 6 h day^−1^ over 3 weeks,^[^ ^83^, ^97^ ^]^ but no impact on bone microarchitecture was visible.^[^ ^83^ ^]^ Importantly, PEMF stimuli of much higher frequencies, as the ones applied to rabbit tibiae bearing titanium dental implants (11.8 kHz pulse, 20 MHz bursts, 0.5 h day^−1^, for 21 or 42 days), did not significantly alter histological morphology or removal torque when compared to controls.^[^ ^95^ ^]^

Implant osseointegration was also evaluated in low BMD conditions, namely in type‐1 diabetes mellitus (T1DM) rabbit models and in glucocorticoids‐treated rabbits.^[^ ^87^, ^88^ ^]^ Both studies used the same porous implants on rabbits’ hindlimbs, and equal PEMF stimuli (15 Hz, 2.0 mT, for 2 h day^−1^), only differing in the assay's duration: 8 weeks for T1DM rabbits and 4 weeks for glucocorticoid‐treated animals.^[^ ^87^, ^88^ ^]^ In both cases, PEMF stimulation could revert the bone microarchitecture deterioration associated with each model and increase bone formation.^[^ ^87^, ^88^ ^]^ Compared to controls, PEMF highly increased BIC (≈4‐fold) and bone volume (≈+70%) in both models, Tb.N (+54.7% in T1DM; +32.1% in glucocorticoid‐treated), cortical thickness and area (+15.2% and +14.9% for T1DM; +19.2% and +28.2% for glucocorticoid‐treated), among other parameters (Table S2, Supporting Information). Bones were also mechanically stronger, given their increased elastic modulus (up to +30%) and hardness (up to +39.6% for T1DM, +28.7% for glucocorticoid‐treated).^[^ ^87^, ^88^ ^]^ Osteoblastogenesis markers of the Wnt/β‐catenin pathway were increased by PEMF in T1DM rabbits,^[^ ^87^ ^]^ while the balance between positive and negative osteoblast regulators has improved in the serum of PEMF‐stimulated glucocorticoid‐treated rabbits (e.g., ≈+40.0% OC and P1NP).^[^ ^88^ ^]^ PEMF induced no effects on circulating bone‐resorption markers (RANKL/RANK signaling; CTX‐1; TRACP5b).^[^ ^87^, ^88^ ^]^

#### 2.3.3 Tackling Soft Tissue Disorders in Intermediate‐Sized Animals via IC Stimulation

Regarding soft tissue healing applications, rabbits subjected to patellectomy and further exposed for 0.5 h day^−1^ for 8–16 weeks to CMF (alternating stimuli of 76.6 Hz, 0.04 mT; constant stimuli of 0.02 mT), experienced significant histological benefits for newly formed bone (+99.2%) and regenerated fibrocartilage zone (+41.9%). Significant biomechanical benefits were also observed but only at week 16: load to failure (+25.9%), ultimate strength (+23.7%), and energy to failure (+67.3%).^[^ ^130^ ^]^

### 2.4 Effects of IC Stimulation on Medium‐Sized Animals

Dogs (*Canis lupus familiaris*) were not widely used to assess IC stimulation (Figure 1) but can offer some advantages when studying the bone response to injury and treatments. Their skeleton is similar to humans in bone weight, density, organic and inorganic composition, water fraction, secondary osteons, epiphyseal fusion after maturity, intracortical remodeling activity, age‐associated bone loss, and poor ability to regenerate cartilage defects. However, dogs’ skeleton differs from humans’ by retaining plexiform bone on cortical periosteal and endosteal surfaces, having thinner articular cartilage and higher annual trabecular turnover rates, which translates into higher rates of solid bony fusion and lower rates of non‐union, comparatively to humans.^[^ ^104^ ^]^

#### 2.4.1 Managing Bone Defects in Medium‐Sized Animals Through IC Stimulation

The earliest evidence of PEMF effects on bone dates back to 1974 with a study on bone defects, when dog fibular osteotomies were stimulated for 24 h day^−1^ with PEMF of 1 or 65 Hz over 4 weeks.^[^ ^73^ ^]^ Although both settings accelerated bone repair, the higher pulse frequency (65 Hz), above the naturally occurring 1 Hz and 2 mV cm^−1^ biologic events, yielded better outcomes, namely predominantly parallel orientation of the callus fiber bundles, accelerated ossification patterns, and reduced cartilage. These histological features translated into improved mechanical strength (+114.3% load‐bearing) at 28 days post‐fracture.^[^ ^73^ ^]^

Other findings in dogs also support the use of PEMF to repair bone defects and improve the success rate of bone (spine) fusion.^[^ ^62^, ^131^ ^]^ Exposure of osteotomized tibiae for 1 h day^−1^ to PEMF of 0.2 mT and 1.5 Hz (asymmetrical pulses), has enhanced callus formation by 53%, and its maturation in the bone healing late‐phase. Increased formation of new bone was histologically demonstrated, with more bone (+1.63‐fold) and fibrous (+1.33‐fold) tissues, and less cartilage (0.71‐fold) in the maturating callus. Also, stimulated tibiae had faster recovery upon load‐bearing (+1.46‐fold) and higher mechanical strength (+20.4% torque and +54.6% torsional stiffness).^[^ ^62^ ^]^ Dogs that have undergone spine fusion and received 1.5 Hz PEMF for 6 h day^−1^ over 24 weeks, had significantly increased BMD of the anterior vertebral body (+12%), and slightly increased flexion and bending stiffness.^[^ ^131^ ^]^

#### 2.4.2 IC Stimulation of Medium‐Sized Animals with Osteoporosis and Cartilage Defects

In OVX osteoporosis dog models, PEMF of 1.5 Hz for 1 h day^−1^ during 12 weeks reduced bone loss from 23.1% to 9.5%, but had no effect on bone remodeling within the bone cortex.^[^ ^132^ ^]^ For combined therapies studies, a PEMF of higher frequency and strength (75 Hz, 1.5 mT) was applied for 6 h day^−1^ over 13 weeks to dogs with articular cartilage defects on the stifle joint (knee), treated with tissue‐engineered osteochondral grafts.^[^ ^133^ ^]^ The PEMF‐stimulated group exhibited improved cartilage growth and repair and was less likely to develop osteochondral pathologies (−80% and −60% probabilities of proteoglycan and chondrocyte pathologies, respectively; −70% probability of worse Osteoarthritis Research Society International (OARSI) score). Slightly improved grafts’ integration was observed, although not statistically significant probably due to changes in PEMF orientation derived from animals’ movement.^[^ ^133^ ^]^

#### 2.4.3 IC Musculoskeletal Studies on Medium‐Sized Animals Showing No Improvement

Studies that did not report any IC‐driven improvements in dogs’ bone metabolism have tested the effects of i) a PEMF of 1.5 Hz, 0.1 mT, 0.5 or 1 h day^−1^ for 12 weeks, on spine fusion rate and bone properties,^[^ ^134^ ^]^ and ii) a PEMF of 40 kHz pulses (and 1.5 MHz bursts), 0.8 mT, 20 min day^−1^ for 2 weeks, on osseointegration of dental implants placed in the dog's mandible.^[^ ^89^ ^]^ Noteworthy, PEMF of 1.06–2.56 mT have been also applied for 24 h day^−1^ over 2 or 6 weeks to tibial osteotomies in sheep (*Ovis aries*), but no significant differences in healing time or bony callus composition were observed with these stimulation parameters.^[^ ^75^ ^]^

### 2.5 Findings from IC Pre‐Clinical Studies

**Figure 3:**
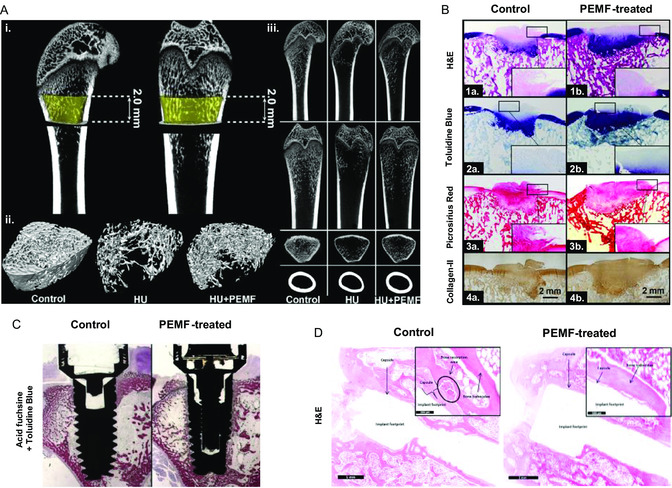
Examples of biological outcomes commonly assessed in IC stimulation studies using animal models. A) Improved trabecular bone microarchitecture and cortical bone thickness of femurs of rats subjected to hindlimb unloading (HU) when exposed to PEMF for 4 weeks: i) Volume of interest (VOI); ii) 3D μCT images of trabecular bone microarchitecture in the VOI; iii) 2D μCT images of trabecular bone microarchitecture (different observation planes). Reproduced and adapted with permission.^[^ ^120^ ^]^ Copyright 2014, Wiley. B) Histological assessment of osteochondral repair in dogs upon 3‐month PEMF treatment: general overview of the tissue with hematoxylin and eosin (H&E, 1a–1b); toluidine blue (2a–2b) for proteoglycan visualization; picrosirius red (3a–3b) for collagen staining and immunohistochemical staining of collagen‐II (4a–4b). Better tissue structure and increased content on proteoglycans and type II collagen were observed in the PEMF‐treated tissues. Reproduced and adapted with permission.^[^ ^133^ ^]^ Copyright 2020, Wiley. C) Histological evaluation of the peri‐implant bone in rabbits, 2 weeks post‐implantation surgery, using acid fuchsine and toluidine: newly formed bone is seen around the implant in both control and PEMF‐treated groups, but in this last the bone tissue is better organized and trabeculae are present on the implant's surface. Reproduced and adapted with permission.^[^ ^86^ ^]^ Copyright 2016, Wiley. D) Histological analysis with H&E of the peri‐implant bone tissue in rats, showing a better tissue organization and increased formation of bone trabeculae around the implant in PEMF‐treated tissues. Reproduced and adapted with permission.^[^ ^90^ ^]^ Copyright 2018, Elsevier.

Pre‐clinical studies in non‐human animal models have gathered evidence on the benefits of IC stimulation to treat bone and cartilage disorders, as illustrated in **Figure** **3**. These included faster callus formation, bridging and bone maturation; improved bone volume and microarchitecture (higher trabecular number, area, and/or thickness, Figure 3A); increased BMD and prevention of bone volume loss, associated to conditions like osteoporosis; increased osseointegration (enhanced peri‐implant bone volume and bone‐implant contact, Figure 3C,D). Histological techniques revealed IC‐induced increases in callus fibrous content, mineral apposition rate, faster cartilage‐to‐bone transition, and better cellular and tissue organization (Figure 3B). Bone mechanical properties, including bone bending stiffness, callus/bone elastic modulus, torque and load bearing, have also improved upon IC stimulation. At a molecular/biochemical level, IC stimulation activated osteogenic pathways as the Wnt/β‐catenin signaling, increased molecules key to bone mineralization (e.g., deposition of collagen‐I and Ca^2+^),^[^ ^135^ ^]^ as well as increased serum levels of osteogenic markers (e.g,. ALP, Ca^2+^, OC, OPG, CTXs) while lowering osteoclastogenesis ones, like RANKL and TRACP5b.

A general overview of IC stimulation efficacy in animal studies is presented in Figure 2, which presents improvements versus no improvements according to stimuli frequency, MF strength or exposure time. Overall, stimuli with pulse frequencies >7.5 Hz performed better, with success rates of 93.4% (57 out of 60 assays), against a success rate of 53.8% for pulse frequencies ≤7.5 Hz (7 out of 13 assays). Accordingly, studies comparing different frequencies inferred the same: higher frequencies usually outperformed lower ones,^[^ ^69^, ^73^ ^]^ and studies on rats and dogs using quite low frequencies were non‐effective.^[^ ^61^, ^69^, ^73^, ^134^ ^]^ The same occurred for MF strengths ≥1.2 mT (93.3% success rate, corresponding to 42 out of 45 assays) when compared to weaker fields (56.5%, 26 out of 46 assays). Exposure to stimuli for at least 3.5 h day^−1^ led to higher success rates (89.3%, 25 out of 28 assays), compared to shorter exposures (78.0%, 39 out of 50 assays). Indeed, when comparing different daily exposure times (0.5, 3, and 6 h per day), longer exposure enhanced the effects of early treatments.^[^ ^67^ ^]^ Importantly, the application of IC stimulation at earlier phases of the injury/condition may improve healing effects and prevent BMD loss.

**Figure 4:**
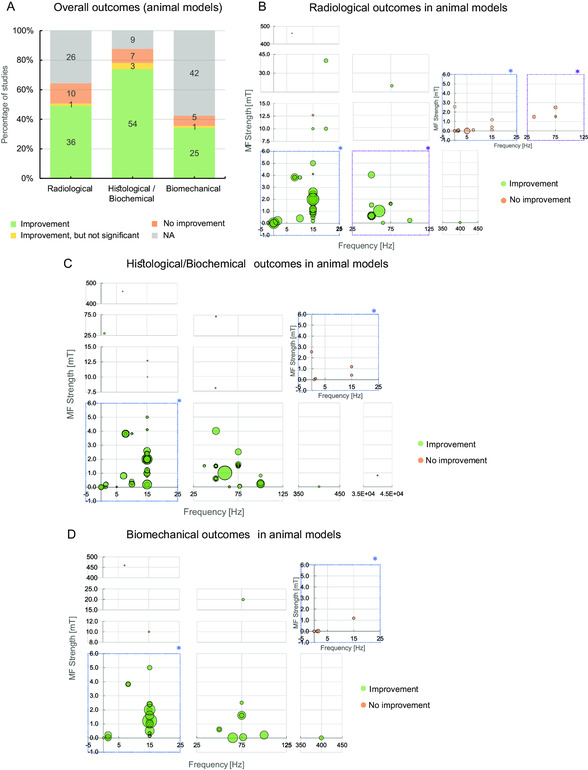
Efficacy analysis of IC stimulation parameters (magnetic field and frequency) on pre‐clinical studies with animal models, based on the bone‐associated outcomes reported. A) Overview of the IC efficacy on the three outcome categories (details in Table S1 and S2, Supporting Information). The number of studies is indicated in the graph. “NA”, not applicable (outcome not assessed). B) Radiological, C) Histological/biochemical and D) Biomechanical outcomes, according to the combination of stimulus’ frequency and MF strength used in each study (when undefined by the authors, the parameter was taken as “0”). Each circle represents one study, and its diameter is directly proportional to the amplitude of the IC stimulation effect. Figure S2–S4, Supporting Information, show these efficacy analyses considering the combination of stimulus’ frequency and MF strength per animal model. Asterisks (*) represent graph sections that show separately the studies with improvements and no improvements, for better visualization.

Overall, whenever biological outcomes were assessed, IC stimulation induced improvements in more than 75% of the assays, regardless of the assessed outcome (**Figure** **4**A). Nevertheless, radiological and biomechanical outcomes were not assessed as widely as histological and biochemical ones. A deeper analysis of the improvement effect sizes according to the combination of frequencies and MF strengths, is presented in Figure 4B–D which shows various effective pairs of frequency/MF combinations in green (e.g., 15 Hz/2.0 mT, 75 Hz/1.6 mT). However, these graphs may include some bias derived from directly comparing effect sizes of studies using different animal models, heterogenous designs and assessments. For example, tibial bone conditions seem to overall respond less to PEMF than femoral ones, in intermediate‐ and medium‐sized animal models.^[^ ^65^, ^66^, ^77^ ^]^ For comparison purposes, efficacy analyses separated per small‐sized, intermediate‐sized, and medium‐sized animal models are presented in Figure S2–S4, Supporting Information. These analyses reveal that IC stimulation induced much higher improvement rates (in 64–72% of the assays) in small and intermediate‐sized animals like rats and rabbits. The most effective (and most tested) frequency/MF combinations for these animal models were 15 Hz/1.6–2.0 mT (Figure S2–S3, Supporting Information). Conversely, IC stimulation in medium‐sized animals such as dogs and sheep, induced improvements in 44% (at most) of the biological outcomes analyzed. Surprisingly, a combination of relatively low frequency and MF (1.5 Hz, 0.2 mT) was quite effective in the medium‐sized animals (Figure S4, Supporting Information). Also to highlight, some studies did not observe any benefits of applying IC stimuli.^[^ ^59^, ^61^, ^74^, ^75^, ^89^, ^95^, ^103^, ^109^, ^134^, ^136^ ^]^ This absence of improvement upon IC stimulation may be ascribed to the use of novel IC modalities that still require optimization, like RMF, or to the relatively lower frequencies, field strengths, or exposure times used (Table S1, Supporting Information). Of note, CMF and PEMF were directly compared in some studies and presented similar efficacies,^[^ ^67^ ^]^ except when CMF was applied intra‐medullary.^[^ ^68^ ^]^ Given its invasiveness, intra‐medullar CMF may be more effective if combined with traditional orthopedic internal fixations.

In general, the studies applying IC stimulation to animal models revealed promising success rates, although radiological and biomechanical outcomes should be more thoroughly assessed. The available data reinforce the potential of IC stimulation as a safe and effective non‐invasive method to improve several musculoskeletal conditions. Notwithstanding, “size matters”, since the success rate of IC stimulation decreases with the increase of the target animal, highlighting the importance to test several stimuli parameters when scaling up an IC intervention to bigger animal models, until reaching humans.

## 3 Clinical Studies of IC Therapies

### 3.1 Overview of IC Clinical Designs

The clinical application of IC stimulation to treat multiple complications has been under development since the middle of the 20^th^ century. This therapeutic is gaining increasing popularity in clinical practice, due to its non‐invasive nature and the wide range of clinical applications of the delivered magnetic fields. So far, the only IC stimulation devices approved by the FDA are those related to bone growth stimulation, used to treat fracture non‐unions and delayed unions and to enhance bone formation after ankle, lumbar, and cervical spine fusion surgeries.^[^ ^137^, ^138^, ^139^ ^]^ Delving deeper into the clinical application of IC stimulation, our search retrieved 44 studies on the therapeutic potential of the IC stimulation to treat musculoskeletal conditions in humans (Table S3, Supporting Information).

**Figure 5:**
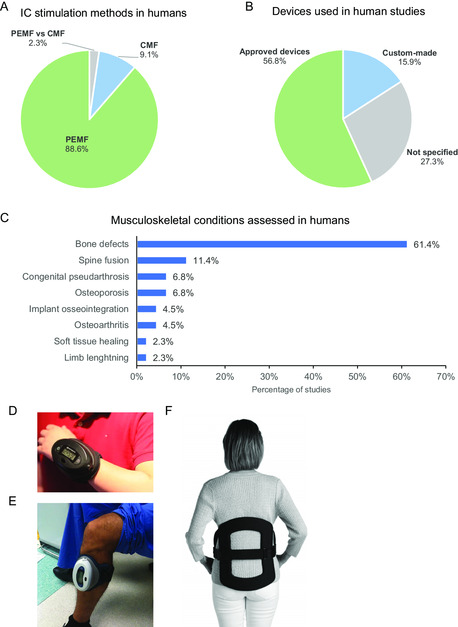
General trends of IC clinical stimulation in clinical studies. Types of A) IC stimulation applied, and B) Stimulation devices used in human patients. “Approved devices” refer to stimulators that are approved for clinical use and available in the market, while “Custom‐made” stimulators are devices developed by the research group to test new setups. C) Musculoskeletal conditions evaluated by the 44 clinical studies here reviewed (details in Table S3, Supporting Information). “Bone defects” include non‐unions and delayed unions mainly from traumatic etiology (fractures). D,E) Photographs of the FDA‐approved PEMF device Orthofix Physio‐Stim used in the clinical practice for scaphoid and tibial non‐unions, respectively. Reproduced with permission.^[^ ^168^ ^]^ Copyright 2017, Springer. Reproduced with permission.^[^ ^138^ ^]^ Copyright 2012, BioMed Central. F) Photographs of the FDA‐approved PEMF device Orthofix Spinal‐Stim used in clinical practice for spinal fusion. Taken from Orthofix Spinal‐Stim Instruction Manual (Model 5212), available online at htps://orthofix.com.

IC clinical studies mainly used devices delivering PEMF (in 88.6% of the studies) (**Figure** **5**A). FDA‐approved stimulators are the most used devices to test the efficacy of IC musculoskeletal therapy in human patients (used in 56.8% of the studies), as well as the stimuli parameters and protocols recommended by their manufacturers (Figure 5B). Most popular models include the “Biomet EBI Bone Healing System”, the “Orthofix Cervical‐Stim”, the “Orthofix Physio‐Stim” (Figure 5D,E) and the “Orthofix Spinal‐Stim” (Figure 5F). Several of these devices allow patients to wear PEMF devices over surgical dressings, placed on the skin over the fracture site. Still, 15.9% of studies used custom‐made devices, while 27.3% did not specified the device used. By far, the most frequent application of IC stimulation on human patients is to heal non‐unions and delayed unions, mainly deriving from fractures (Figure 5C “bone defects”, 61.4% of the studies). Spine fusion has been the second target application, although it only corresponds to 11.4% of the studies.

**Figure 6:**
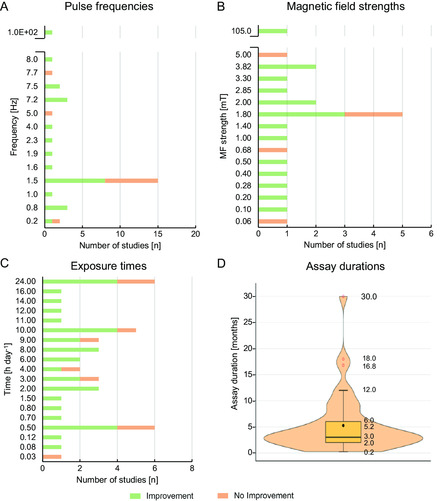
IC stimuli parameters applied in clinical studies. Types of IC stimulation parameters used on patients with musculoskeletal disorders, number of studies in which they were used, and their relative success, in the 44 clinical studies here reviewed (details in Table S3 and S4, Supporting Information). A) Pulse frequencies used (in Hz); of note, 12 studies had non‐defined pulse frequencies and 1 study tested 2 different pulse frequencies, totalizing the 33 pulse frequencies here presented. B) List of the applied magnetic field (MF) strengths (in mT); of note, 23 studies had non‐defined MF strengths and 1 study tested 2 different MF strengths, totalizing the 22 MF strengths here presented. C) Daily exposure time to the stimulus (in hours day^‐1^); of note, 4 studies had non‐defined exposure times, 1 study tested 3 different exposure times, and 1 study tested patient‐dependent exposure times, totalizing the 42 exposure times here presented. D) Box‐violin plot with the distribution and summary statistics of the total duration of the IC stimulation assays, in months.

Regarding the IC stimuli parameters applied in clinical studies (**Figure** **6**A–D), there is first to highlight that the clinical studies reported less frequently the stimuli parameters applied than the pre‐clinical ones, with only 75% of the studies having disclosed at least one parameter. Some studies only mentioned the device used, while others did not present any setup information. In the fewer clinical studies that could be analyzed here, the stimuli pulse frequencies ranged from 1.5–80 Hz, with a high prevalence of 15 Hz, the frequency used in 44.1% of the studies that have provided the stimuli specifications. A much higher frequency (1 kHz pulses; 27 MHz bursts) was also tested on soft tissue after tooth removal.^[^ ^140^ ^]^ Only half of the studies (22 out of 44) provided the values for the applied MF strengths. These highly varied from 6 μT to 5 mT (except for a higher MF strength of 105 mT), although 45.4% (10 out of 22 studies) applied MF strengths between 1.0 and 3.0 mT. The daily exposure time ranged from 2 min to a full day exposure, with an average and 3^rd^ quartile value of 7.9 and 10 h day^−1^, respectively, according to the manufacturers’ recommendations of at least 8–10 h day^−1^ exposure for FDA‐approved devices. Studies durations varied from 6 days to 18 months, with a median of 3 months and an average of 5.4 months.

### 3.2 Evidence of IC Clinical Efficacy in Humans

**Figure 7:**
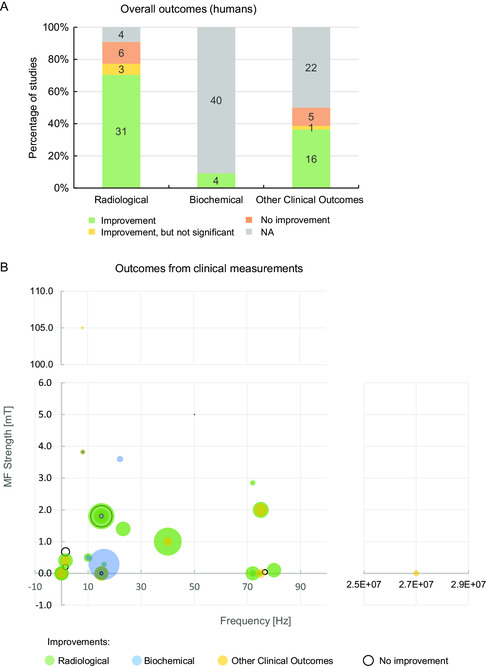
Analysis of the efficacy of the IC in vivo stimulation parameters (magnetic field and frequency) on human patients, based on the reported bone‐associated outcomes. A) Overview of the IC efficacy on the three categories of outcomes (qualitative and quantitative details in Table S3 and S4, Supporting Information, respectively). The number of studies is indicated in the graph. “NA”, not applicable (outcome not assessed). B) Radiological (green), Histological/biochemical (blue) and Biomechanical (yellow) outcomes, according to the stimulus’ pulse frequency and MF strength used in each study. Each circle represents one study, and its diameter is directly proportional to the amplitude of the effect that IC stimulation had on that outcome category. Values of ±0.1 and zero (0) were chosen to represent non‐stated outcomes and unknown pulse frequencies or MFs strengths, respectively.

Regarding the type of non‐invasive biological/clinical outcome measures used to assess the efficacy of IC stimulation, radiological imaging outcomes were the ones mainly assessed in almost all the clinical studies (40 out of 44 studies; **Figure** **7**A). In contrast, the influence of IC stimulation on serum biochemical markers was only addressed in four studies. Other clinical outcomes, namely biomechanical ones, were reported in 50% of the studies (Figure 7A). Importantly, the use of IC stimulation was not associated with any adverse effects and is worth highlighting that patients receiving IC stimulation generally reported better pain management.^[^ ^140^, ^141^, ^142^, ^143^, ^144^, ^145^, ^146^, ^147^, ^148^ ^]^

#### 3.2.1 Managing Bone Defects in Humans with IC Stimulation

Success when treating bone defects, like delayed unions and non‐unions, was obtained upon stimulation with PEMF (and CMF) of 15, 23, or 80 Hz pulses, 0.01–2 mT, applied for 8–24 h day^−1^, since radiological analyses revealed faster healing rates and unions were achieved in an average of 91% of cases.^[^ ^141^, ^144^, ^148^, ^149^, ^150^, ^151^, ^152^, ^153^, ^154^, ^155^, ^156^, ^157^, ^158^, ^159^ ^]^ Most of these bone defect studies used commercial devices like the Biomet EBI Bone Healing System, the EBI Bone Healing System Model 420, the Orthopulse I or II, and the OrthoLogic 1000 device. Overall, our analysis of IC stimulation efficacy in treating bone defects, which includes studies from 1982 to 2023, agrees with previous revision studies. According to Heckman et al.^[^ ^160^ ^]^ (1981), 64% of 149 non‐unions healed properly upon PEMF stimulation, while Bassett et al.^[^ ^161^ ^]^ (1982) calculated this stimulation was successful for 76–81% of 1007 ununited fractures. Similar success rates were obtained when comparing PEMF against surgery with bone grafts, to treat ununited tibial fractures. Aaron et al.^[^ ^162^ ^]^ (2004) concluded that both treatments performed equally, with success rates of 82% for bone grafts (569 cases from 14 studies), and 81% for PEMF treatment (1718 cases from 28 studies). This means that therapeutic intervention could have evolved more over the last decades, still highlighting the lack of setup optimization and/or personalized medicine studies.

Examples of setups with positive osteogenic effects include the EBI Bone Healing System Model 420 (15 Hz pulses, 4.5 kHz bursts, 8–10 h day^−1^) to treat scaphoid non‐unions, which resulted in better grip and wrist motion unto 83% and 89% of normal levels, respectively.^[^ ^141^ ^]^ However, in a follow‐up study 6 years later, using the same settings, results slightly decreased to 77% and 80%.^[^ ^152^ ^]^ Authors suggested that the less successful cases may derive from low compliance to the non‐weight‐bearing restriction while using the device.^[^ ^141^ ^]^ Biochemical outputs, such as significant increases in growth factors like PIGF, BDNF, BMP‐5 and ‐7, were also observed after stimulation of metatarsal non‐unions with the Biomet EBI Bone Healing System using similar parameters (15 Hz pulses, 4.5 kHz bursts, 1.8 mT, 10 h day^−1^).^[^ ^163^ ^]^ PEMF stimulation also proved effective in treating congenital tibial pseudarthrosis,^[^ ^164^, ^165^ ^]^ although still needing additional interventions in more severe cases.^[^ ^165^, ^166^ ^]^ Noticeably, less successful outcomes were also observed when using commercial devices. In another study using EBI Bone Healing System, PEMF stimulation (15 Hz, 1.8 mT) of delayed unions resultant from ankle arthrodesis, with immobilization and limited weight‐bearing, only 26% of cases achieved union.^[^ ^167^ ^]^ No improvements in the carpal scaphoid non‐union healing rate occurred using Physio‐Stim (1.5 Hz, 0.2 mT, 3 h day^−1^), with its late application (6 weeks post‐fixation surgery) being suggested as the main cause of failure.^[^ ^168^ ^]^

PEMF efficacy was also tested in acute bone fractures. PEMF of 40–72 Hz, 1 mT, applied 2–6 h day^−1^ after mandibular fracture, complemented with maxillo‐mandibular fixation, was able to return BMD to basal levels, or even increasing it by ≈16.7% after 1‐month post‐surgery.^[^ ^143^, ^146^, ^169^ ^]^ Authors also reported increased stability in mouth opening, with less pain, for the stimulated patients.^[^ ^146^ ^]^ For fractures in other bones, such as scaphoid and tibia, PEMF stimulation at a lower frequency (15 Hz, 1.8 mT) did not accelerate healing, even when applied 10–24 h day−1.^[^ ^170^, ^171^, ^172^ ^]^ Also, no significant differences in the recovery of wrist movement were observed for patients with fractured scaphoids stimulated with Orthopulse PEMF of 15 Hz pulses, for 24 h day^−1^ over 1.5^[^ ^170^, ^171^ ^]^ or 3^[^ ^170^ ^]^ months. Nevertheless, application of PEMF of 16 Hz, 0.006–0.282 mT, for 7 min day^−1^ on osteotomized tibia, increased serum ALP levels and accelerated osseous consolidation in stimulated patients, compared with the placebo group, although this later effect was only significative for patients over 50 years old.^[^ ^173^ ^]^ Also, radius fractures stimulated for 24 h day^−1^ during ≈3 months with PEMF of 10 Hz pulses/20 kHz bursts and 0.05–0.5 mT, delivered by the “Fracture Healing Patch” (Pulsar Medtech Ltd), accelerated bone healing (+29.6% union bridging, +104.3% hand grip strength) and improved wrist function (up to +1.3‐fold).^[^ ^174^ ^]^

Regarding spine fusion, some studies state that both PEMF (4–8 h day^−1^) and CMF (30 min day^−1^) can improve the surgery's success rate by up to 48.8%.^[^ ^145^, ^175^, ^176^ ^]^ In contrast, a comparative study observed that both PEMF of 15 Hz pulses, 0.68 mT, 2 min day^−1^, and CMF of 76.6 Hz, 40.0 ± 8.0 μT AC + 20.0 ± 2.0 μT DC, 30 min day^−1^, performed worse than the unstimulated group, which achieved solid fusions in 100% of the treated patients, against 68.8% for PEMF and 87.5% for CMF.^[^ ^177^ ^]^ Nevertheless, when comparing these stimuli parameters with others reviewed in this section, we can denote that PEMF/CMF daily exposure times and CMF‐induced MF strength are much lower than the average of all clinical studies (7.9 h day^−1^ and 6.3 mT; Figure 6 and Table S3, Supporting Information), what may explain the worse success rates. In line with this hypothesis, a recent multicenter study on the use of PEMF as an adjuvant therapy to lumbar spine fusion in 142 patients at risk for pseudarthrosis (Orthofix SpinalStim, 1.5 Hz pulses, 3.85 kHz bursts, 0.4 mT, 2 h day^−1^ over 6 months), reported that 88.0% of patients exhibited successful fusions and significant improvements in pain, function, and quality of life at a 12 months follow‐up appointment.^[^ ^178^ ^]^ Interestingly, a study that computationally compared the electric current densities induced by the FDA‐approved PEMF stimulator (Orthofix SpinalStim), the CMF stimulator (SpinaLogic) and the CC stimulator (Biomet SpinalPak), reported that PEMF induces the strongest maximum electric field and current density amplitudes in spinal vertebrae, besides generating local micromechanical forces that were more similar to the micromechanical oscillations naturally generated by EMF in the bone.^[^ ^179^ ^]^

#### 3.2.2 Tackling Osteoporosis in Humans Through IC Stimulation

In post‐menopausal women prone to osteoporosis and taking calcium and vitamin D supplements, a 40 min day^−1^ thrice‐a‐week treatment with PEMF of 8 Hz pulses and 3.82 mT only slightly increased vertebral, femoral neck, and hip BMD (+≈1.5–2.0%) at the end of the 6‐week treatment, and significantly reduced bone marrow fat fraction (−4.81%).^[^ ^180^ ^]^ Osteoporotic patients who suffered bone fractures and were stimulated with the same PEMF parameters showed improvements in bone microarchitecture (average of +7.2% BV/TV, +24.8% cortical thickness (Ct.Th), +19.0% Tb.Th, +13.4% Tb.N), as well as in health scores related to body functions, quality of life and back pain.^[^ ^181^ ^]^ In other study on osteoporotic patients under calcium supplementation, a 10 h day^−1^ (overnight) PEMF stimulation of the non‐dominant forearm with higher frequencies (72 Hz, 2.85 mT), resulted in increased radii BMD, that further decreased upon 36 weeks after the last stimulation. Interestingly, the radii from the unexposed forearm experienced a similar pattern, suggesting a systemic “cross‐talk” or close proximity of both radii during asleep stimulation.^[^ ^182^ ^]^ Bone‐related biochemical markers altered in osteoporotic patients treated with PEMF of lower frequency but higher MF (8 Hz, 3.82 mT), including serum ALP (+3.23%) and CTX‐I levels (−9.12%).^[^ ^180^ ^]^ Additionally, a study on women with post‐menopausal osteoporosis, testing PEMF rotating every 4 min in frequencies varying between 16, 18, 20, and 22 Hz, and MF strengths between 3.0, 3.2, 3.4, and 3.6 mT, for 0.8 h day^−1^ during 2 weeks, reported significant changes in serum markers related to the Wnt/β‐catenin and the OPG/RANKL pathways, which can explain the positive effects of this IC stimulation on bone metabolism.^[^ ^183^ ^]^

#### 3.2.3 Enhancing Implant Osseointegration in Humans via IC Stimulation

To our knowledge, PEMF efficacy on implants’ osseointegration in humans was only tested in dental and hip prosthesis implants.^[^ ^142^, ^184^ ^]^ For dental implants, PEMF was implemented through caps that delivered continuous daily stimulation (no parameters were provided). From 1.6 months onward, the active caps promoted overall higher implant stability (average +10.2% in stability scores vs. unstimulated controls).^[^ ^184^ ^]^ On hip prosthesis, BMD levels at 0 and 90 post‐operative days, were measured on different zones following stimulation with PEMF of 75 Hz, 2.0 mT (6 h day^−1^). Although no significant differences were found in the average BMD levels at both timepoints, the percentage of “responder patients” (with >3.5% increase in BMD) was bigger upon PEMF stimulation (66% and 93% in PEMF‐stimulated patients, for 2 measured zones, against 40% and 40% in the non‐stimulated group), proving that PEMF is indeed helpful in clinical recovery and in bone stock restoration.^[^ ^142^ ^]^

#### 3.2.4 Handling Osteoarthritis in Humans Through IC Stimulation

Only two studies tested the efficacy of PEMF on (knee) osteoarthritis. One study used the FDA‐approved MAGCELL ARTHRO device to deliver PEMF of 8 Hz, 105 mT for 5 min twice‐a‐day, for 15 days, and atients reported a reduction in pain, stiffness, and disability in carrying out daily activities, overall improving their health condition.^[^ ^147^ ^]^ Another study assessed the efficacy of PEMF of 50 Hz, 5 mT, for 30 min day^−1^ over 2 months, with and without combination of progressive resistance exercise (PRE), to improve function and pain in patients with knee osteoarthritis. Both PEMF with and without combination of PRE were equally effective in decreasing pain and improving function (in 60–80% of patients), suggesting that optimal PEMF parameters to improve the effects of PRE in knee OA are yet to be determined.^[^ ^185^ ^]^

#### 3.2.5 Addressing Soft Tissue Disorders in Humans with IC Stimulation

In the only study on IC clinical application for soft tissues reported in the literature, PEMF stimuli of very high frequency (1 kHz pulses, with 27 MHz bursts) could improve soft tissue healing, pain management and reduce the risk of dehiscence 1 week post oral surgery, when applied for 24 h day^−1^.^[^ ^140^ ^]^

### 3.3 Findings from IC Clinical Studies

The relative efficacy of the IC clinical studies, according to the applied stimuli parameters, is presented in Figure 6 (global overview for each parameter) and Figure 7, which associates pairs of frequencies and MFs of stimuli with their therapeutic efficacy, based on the three categories of outcome measures.

**Figure 8:**
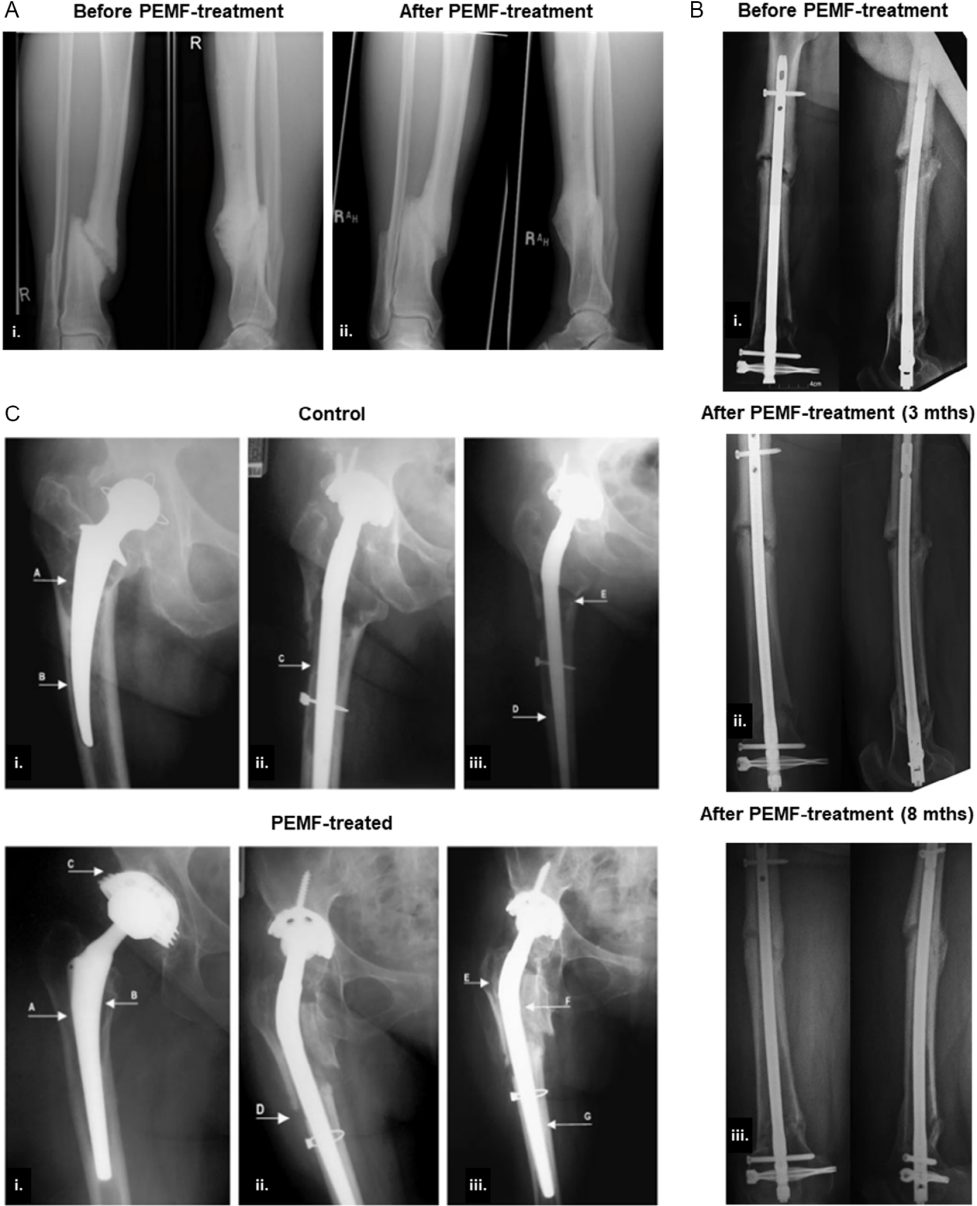
Examples of biological outcomes assessed in IC stimulation studies in humans. A) Anteroposterior and lateral radiographs of a distal tibial nonunion: i) 10 months after fracture; ii) after PEMF stimulation of the fracture site for 5 months, leading to fracture union. Reproduced and adapted with permission.^[^ ^138^ ^]^ Copyright 2012, BioMed Central. B) Delayed union of femoral fracture in a patient who received reduction and intramedullary fixation: i) before PEMF treatment; ii) upon 3 months of PEMF treatment, already showing some progress to union; iii) upon 8 months of PEMF treatment, when the fracture became united. Reproduced and adapted with permission.^[^ ^151^ ^]^ Copyright 2013, BioMed Central. C) Anteroposterior X‐rays of patients with hip prosthesis in a control group (top set of images) or a PEMF‐treated group (bottom set of images): i) pre‐operative; ii) post‐operative; iii) 90‐day‐follow‐up. Images illustrate a higher rate of implant osseointegration in the PEMF‐treated group. Reproduced and adapted with permission.^[^ ^142^ ^]^ Copyright 2009, Wiley.

First, no clinical study has reported major adverse effects from the use of IC stimulation devices. Studies assessing radiographic outcomes (examples illustrated in **Figure** **8**) have reported effects driven by IC stimulation that include faster osseous consolidation and healing of non‐unions and delayed unions (Figure 8A,B), prevention of BMD loss immediately after injury and, in some cases, increases in BMD upon prolonged treatment (Figure 8C). The few studies that measured serum markers observed increases in osteogenic‐associated markers, such as ALP and specific growth factors. Overall, human studies with IC simulation could benefit from analyzing biochemical outcomes (e.g., osteogenic markers in serum) more frequently, as such analyses may help to understand the signaling effects of IC stimulation. In general, the IC stimulation setups used had low efficacy for treating scaphoid non‐unions and delayed unions after foot and ankle arthrodesis, and effectiveness on bone fractures highly varied. Indeed, various setups did not significantly improve the bone‐associated main endpoint.^[^ ^171^, ^177^, ^186^ ^]^ This may be related to the stimuli parameters applied, but also to the tested condition, since several unstimulated control groups have achieved quasi‐optimum recoveries in the assay duration time. Nevertheless, IC stimulation improved patient condition in secondary endpoints, like recovery time, pain management or prevention of bone mass loss.^[^ ^171^, ^186^ ^]^

Many authors did not report the IC parameters used in their studies, making it difficult to draw more assertive conclusions regarding effective magnetic stimuli settings. However, most of them used clinically approved devices, mainly Biomet EBI Bone Healing System, which generally deliver PEMF of 15 Hz quasi‐rectangular pulses, followed by a sharper reverse form, generating MF strengths up to 1.8–2.0 mT. Most studies using this device and respective setups have achieved significant positive outcomes on non‐unions and delayed unions, bone fractures, and consolidation of spine fusions. With two exceptions, all setups using >15 Hz stimuli presented positive osteogenic effects, and stimuli generating MFs ≥1 mT presented improvements in 80% of cases (Figure 6A and 7B). Regarding the exposure time, 11 out of 43 studies (25.6%) applied the stimuli for the average recommended 8–10 h interval of daily stimulation, with 9 of them reporting osteogenic improvements. Figure 7B highlights 15 Hz/1.8 mT; 16 Hz/0.282 mT, and 40 Hz/1 mT as top‐performing frequency/MF combinations in human clinical studies of IC stimulation.

Altogether, the use of IC stimulation seems clinically feasible to treat non‐unions, to reduce its risk after fracture and accelerate healing when combined with standard procedures, and to increase the bone fusion success rate.^[^ ^138^, ^139^ ^]^ Further, although still few, the promising results here presented for implants’ osseointegration, stress the need to further investigate the benefits of IC stimulation on this musculoskeletal condition, a main societal challenge associated with high numbers of primary and revision surgeries. Although IC efficacy has been proved in animal models of osteoporosis, few clinical trials have tested it, most likely due to the burden associated with the prolonged use of the device. Lastly, studies on IC's potential beneficial effects on osteoarthritis are still lacking and are highly demanded.

Overall, this study indicates that it would be desirable to develop more attractive devices for daily use, with the ability to provide personalized stimuli, and highlights the need to comprehensively optimize the stimuli setups to increase IC clinical performance.

## 4 The Potential Biomechanism Behind IC Therapeutic Effects

**Figure 9:**
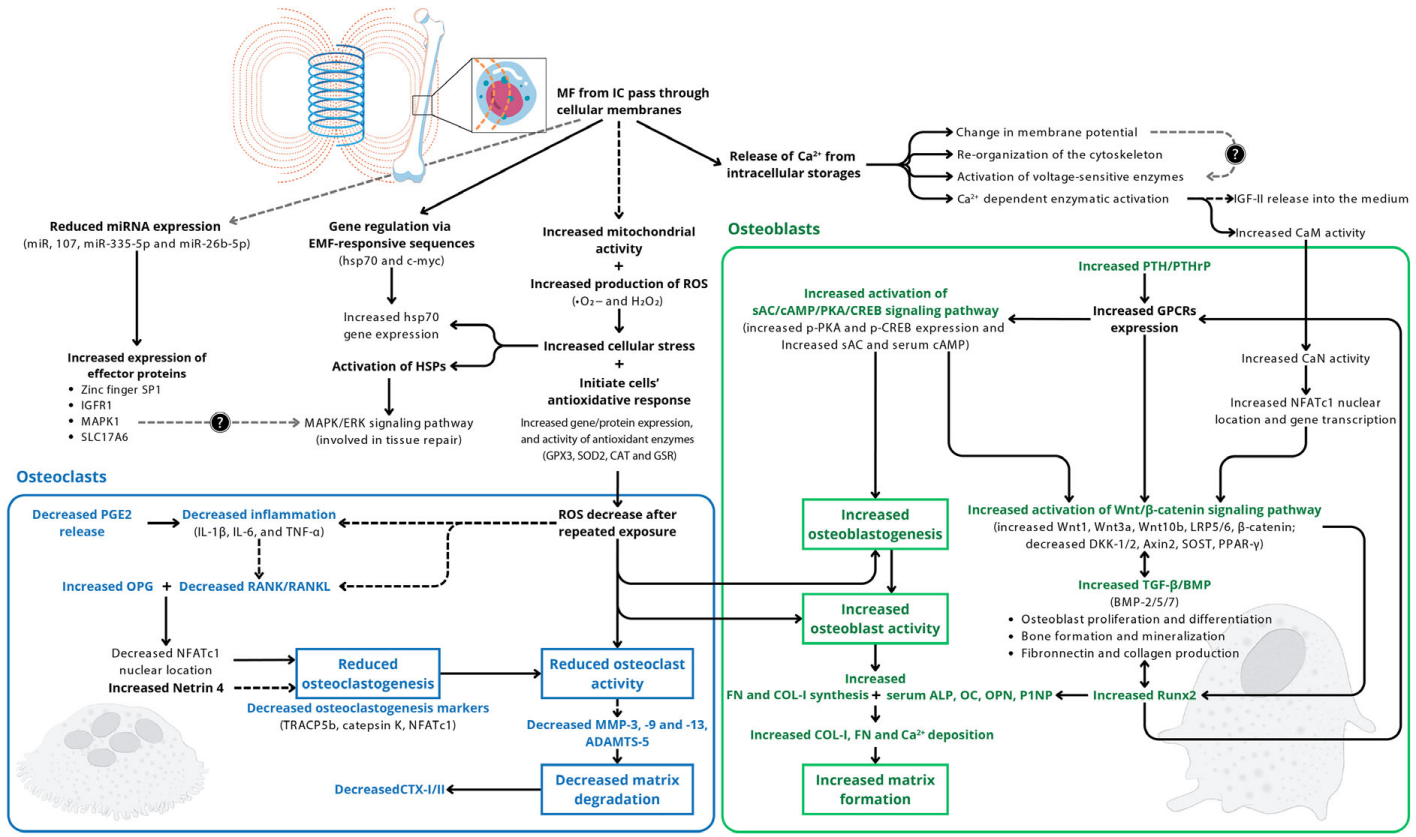
Biomechanisms that may underly the observed therapeutical bone‐associated effects of inductive coupling (IC) in vivo stimulation. The electromagnetic fields (EMF) delivered by IC pass the cytoplasmic membrane of cells, such as osteoblasts and others, to induce the release of intracellular calcium, alterations in the membranes’ potential, and elicit a series of other molecular alterations (e.g., signaling pathways, biological processes) whose interplay culminate in the promotion or repression of the expression of genes associated to increased osteoblastogenesis and osteoblast activity, and decreased osteoclastogenesis and osteoclast activity. The green squares delimit events mostly observed in osteoblasts, while blue squares delimit events mostly observed in osteoclasts. Black bold headings denote pathways and mechanisms reported only in vitro, while colored bold headings denote pathways and mechanisms already observed in vivo; headings surrounded by colored boxes represent the main cellular endpoints observed in most in vivo studies upon IC stimulation. Continuous black arrows represent associations already reported between the indicated cellular events, while dashed black arrows represent indirect associations that have other intermediary steps; dashed grey arrows represent probable associations.

At the turn of the 21^st^ century, various groups started attempting to infer the biomechanisms underlying the IC effects on the musculoskeletal system, with this line of research accelerating in the last 5 years. A sum up of these authors’ findings is illustrated in **Figure** **9**. It is currently assumed that the electromagnetic fields generated by IC can pass through the cellular membranes and elicit a release of Ca^2+^ from intracellular storages, increasing the cytosolic Ca^2+^ concentration. This potentially occurs through the activation of ryanodine‐dependent Ca^2+^ channels (RyR) or inositol 1,4,5‐triphosphate receptors (IP_3_R) of the endoplasmic reticulum and mitochondria.^[^ ^187^, ^188^, ^189^ ^]^ This first cellular event induces changes in the membrane polarization, coupled with the activation of voltage‐sensitive enzymes, the reorganization of the cytoskeleton, and the activation of Ca^2+^‐dependent enzymes, including calmodulin (CaM).^[^ ^190^ ^]^ In osteoblasts, calcineurin (CaN) becomes active in a Ca^2+^ and CaM‐dependent manner,^[^ ^191^ ^]^ and dephosphorylates Nuclear Factor of Activated T Cells 1 (NFATc1), promoting its nuclear translocation and a subsequent increase in the transcription of genes related to the Wnt/β‐catenin pathway.^[^ ^192^ ^]^ Following IC application in vivo to osteoporosis, osteoarthritis and implant osteointegration conditions, constituents of this pathway (Wnt1, Wnt3a, Wnt10b, LRP5/6, β‐catenin) were observed to be more expressed or active, both in human and non‐human studies; contrarily, endogenous inhibitors of this pathway (DKK‐1, Axin2, SOST, PPAR‐γ) had their gene expression reduced.^[^ ^87^, ^88^, ^98^, ^120^, ^122^, ^123^, ^124^, ^127^, ^183^, ^193^ ^]^ Another widely known osteogenic signaling pathway that cross‐talks with the Wnt pathway is the TGF‐β/BMP (2/5/7) signaling pathway, which is also found to increase following IC stimulation.^[^ ^111^, ^124^, ^163^, ^193^ ^]^ Wnt and BMP pathways can intricately regulate each other through synergistic signaling and feedback systems at all levels (ligand production, interaction of intermediates and effectors).^[^ ^194^, ^195^, ^196^, ^197^ ^]^ Also observed to be increased upon IC stimulation is the Runt‐related transcription factor 2 (Runx2), a target gene of these pathways that induces osteogenesis^[^ ^198^, ^199^ ^]^ and osteogenesis‐related markers (e.g., OC).^[^ ^98^, ^123^, ^124^ ^]^ Runx2 is also involved in the gene regulation of some G protein‐coupled receptors (GPCR),^[^ ^200^ ^]^ which can be activated by PTH (also increased by IC stimulation^[^ ^121^, ^193^ ^]^) to initiate the sAC/cAMP/PKA/CREB signaling cascade, leading to increased sAC, serum cAMP, p‐PKA and p‐CREB.^[^ ^102^, ^121^ ^]^ This sequence of events promotes osteoblastogenic effects, for example by allowing the accumulation of β‐catenin^[^ ^201^ ^]^ and thus cross‐talking with the Wnt signaling pathway. Increases of the aforementioned osteoblastogenesis markers, as well as of osteoblast activity ones, such as ALP, OC, OPN, and P1NP, together with the increased production and/or deposition of Ca^2+^, collagen‐I and fibronectin (in tendons), may account for the increased matrix and bone formation reported by most of the studies here disclosed.^[^ ^60^, ^65^, ^66^, ^76^, ^78^, ^80^, ^81^, ^88^, ^98^, ^102^, ^108^, ^119^, ^120^, ^121^, ^122^, ^123^, ^124^, ^126^, ^127^, ^173^, ^180^ ^]^

In contrast, IC stimulation can also regulate osteoclast differentiation and activity, by increasing the OPG/RANKL ratio. The increase in OPG expression, together with a decrease in RANKL expression, are thought to be the major events in the IC‐induced decrease of osteoclastogenesis.^[^ ^113^, ^124^, ^127^, ^183^ ^]^ Decreasing the RANK/RANKL signaling leads to a reduction of the activity and nuclear translocation of NFATc1 in osteoclasts, hampering both the NFATc1 auto‐amplification loop and the expression of osteoclast markers, such as cathepsin K and the widely measured TRACP5b.^[^ ^87^, ^88^, ^113^, ^120^, ^121^, ^122^, ^123^ ^]^ Enzymes involved in matrix degradation, such as metalloproteins (MMP‐3/9/13) and ADAMTS‐5, were observed to be decreased as well.^[^ ^125^ ^]^ Subsequently to all these osteoclast‐related molecular events, there is a reduction of bone resorption, and thus a decrease in serum CTX.^[^ ^87^, ^88^, ^102^, ^112^, ^120^, ^121^, ^126^, ^180^ ^]^ Netrin 4 may also play a role in the PEMF‐induced decrease of osteoclastogenesis,^[^ ^202^ ^]^ since PEMF increases the levels of this inhibitor of osteoclast differentiation,^[^ ^203^ ^]^ and consequently of bone resorption, as other netrin proteins (e.g., netrin 1). PEMF effects on osteoclasts may also be related to the desensitization of osteoblasts to inflammation initiators, such as IL‐1β,^[^ ^204^ ^]^ resulting in a decreased expression and secretion of IL‐6 and RANKL, among other cytokines, by these cells.^[^ ^111^, ^115^, ^125^ ^]^

In vitro experiments suggest two other parallel and interacting mechanisms of action by which IC may lead to decreased osteoclast and increased osteoblast activities. PEMF was observed to induce, at an early phase, an increase in mitochondrial activity and production of reactive oxygen species (ROS, •O_2_^−^ and H_2_O_2_), resulting in increased non‐cytotoxic oxidative stress. This event is important to trigger the cell's antioxidative defense mechanisms, through the increased gene and protein expression and activity of antioxidant enzymes, namely superoxide dismutase 2 (SOD2), catalase (CAT), glutathione peroxidase 3 (GPX3) and glutathione‐disulfide reductase (GSR), which consequentially decreases ROS.^[^ ^187^, ^205^ ^]^ The reduction of ROS may play a role in promoting osteoblastogenesis, as well as decreasing the inflammatory response associated with osteoclastogenesis and osteoclast activity.^[^ ^205^ ^]^ Potential contributors to the decreased circulating levels of inflammatory cytokines are the decreased levels of angiogenic factors like VEGF.^[^ ^115^ ^]^ PEMF can also promote other stress‐related processes and associated tissue repair cascades, by inducing the expression of heat shock proteins like hsp70, whose gene's promoters have electromagnetic fields (EMF)‐responsive sequences.^[^ ^206^, ^207^ ^]^ Noteworthy, in a peripheral blood mononuclear cells (PBMCs) model of Alzheimer's disease PEMF was reported to decrease the expression of miRNAs, namely miR‐107, miR‐335‐5p, and miR‐26b‐5p, that regulate the translation of mRNAs associated with transcription factors, growth factors, enzymes, and transporters (e.g., SP1, IGFR1 and MAPK, SLC17A6, respectively).^[^ ^208^ ^]^ Although not directly related to musculoskeletal disorders, this miRNA‐based action mechanism may likely play a role in the clinical outcomes here reported and may be worth studying in the future.

In summary, there are various possible cellular events by which IC stimuli modulate osteogenic and osteoclastic activities to increase bone formation and decrease bone resorption, underscoring IC therapeutic effects in musculoskeletal conditions. A comprehensive study of the less explored mechanisms like the regulation of oxidative and proteostasis stress, of inflammatory responses, and miRNA expression, will deeper our knowledge on IC stimulation mechanisms of action, not only on bone but also on other potential target tissues. Further, it will open new avenues for the potential use of combined therapies, such as PEMF together with a molecular therapy targeting key protein(s) of the core signaling cascades, to boost the IC therapy.

## 5 General Conclusions and Future Perspectives

This review analyzed 117 studies (73 in animal models, and 44 in human patients) using magnetic stimulation delivered by IC devices for musculoskeletal therapy purposes. Overall, from the studies here analyzed, IC stimulation is an attractive option for the clinical management of bone and cartilage disorders. IC is a non‐invasive therapy, with the MF being able to pass the cellular membranes to elicit various intracellular events culminating in tissue repair via e.g., increased bone deposition and decreased bone resorption. However, clinical evidence is still scarce for each condition tested, particularly for implants’ osseointegration, osteoporosis and osteoarthritis in humans. To achieve good clinical evidence, not only a higher number of trials is required, but also more studies comparing different stimuli parameters. The comparative analyses of pre‐clinical in vivo data here presented hold the potential to support the design of such advanced studies. Overall, stimuli with pulses’ frequencies >7.5 Hz in animal studies and >15 Hz for human patients, performed better, while in both pre‐clinical and clinical studies, MF strengths >1.0–1.2 mT were associated with higher success rates. Generally, higher frequencies and MF strengths outperformed the lower ones, as well as longer exposures outperformed shorter ones. Noteworthy, the effect sizes recorded on human patients are generally lower than in animal studies (and in medium‐sized animals lower than in small‐sized ones), possibly because the regeneration potential decreases with increased animal size and complexity, again strengthening the need for further optimization of stimuli clinical setups. Other major limitations of the current approach to IC therapeutics include the delivery of stimuli based on parameters found empirically (usually by the trial‐error method), and the fact that it is statically maintained throughout the treatment. IC stimulation will only be able to achieve its highest impact on bone bioactivity and be fully effective, if it can be used for customized therapy, according to an individual's physiological response.^[^ ^69^ ^]^ Indeed, both extra and intracorporeal electrical stimulators will most likely provide poor therapeutic effects if multiple patient‐related and externally driven factors are not considered, including a wide range of physical, behavioral, social and psychological factors. Optimal personalized stimulation parameters cannot be static: the unpredictability of disorders’ dynamics demands that the stimulus parameters dynamically change to fit the idiosyncrasies of each patient. Such advances will most likely require innovative methods and intelligent technologies, extracorporeally controlled by medical specialists using wirelessly mobile applications.^[^ ^209^ ^]^ Moreover, IC devices for bioelectronic implantable applications require electric powering characterized by very high electric currents (usually exceeding 1 A), which is an additional high‐risk scenario that can trigger post‐surgical complications. Therefore, new magnetic stimulation systems must be designed to deliver targeted magnetic stimuli when electrically supplied by very low current (lower than 100 mA).^[^ ^210^ ^]^ Another line to which IC therapeutics can evolve comprehends smart magnetic bioactive materials, which may contribute to a more localized application of EMF and ultimately improve bone (and other tissues) regeneration.^[^ ^211^ ^]^ Tissue engineering strategies applying such materials could further potentiate the effects of IC stimulation.

Hopefully, the transversal knowledge presented herein will serve as a valuable guide as we navigate the path toward optimizing clinical evidence and personalized IC‐based treatment strategies. A deepen study of the biomechanisms of IC stimulation, the comprehensive testing of various stimuli parameters in the clinical setup, and the development of innovative medical devices, will be vital for pushing forward the clinical implementation and achieve the full therapeutic potential of IC stimulation for musculoskeletal disorders, toward more effective and tailored patient care.

## Conflict of Interest

The authors declare no conflict of interest.

## Supporting information

Supplementary Material
