# Supplementary material for: Gathering Evidence to Leverage Musculoskeletal Magnetic Stimulation Towards Clinical Applicability
Source: Small Sci. 2024 Feb 26;4(5):2300303. doi: 10.1002/smsc.202300303 (PMC11935072; doi:10.1002/smsc.202300303)
Supplement: Supplementary file 1 — Supplementary Material [file SMSC-4-2300303-s001.pdf]

Supplementary Information for

Gathering Evidence to Leverage Musculoskeletal Magnetic Stimulation Towards Clinical Applicability

José G. S. Figueiredo[1,§], Bárbara M. de Sousa[1,§], Marco P. Soares dos Santos[2,3,#,*] and Sandra I. Vieira[1,#,*]

[1)] Department of Medical Sciences, Institute of Biomedicine (iBiMED), University of Aveiro, 3810-193 Aveiro, Portugal.

[2)] Department of Mechanical Engineering, Centre for Mechanical Technology & Automation (TEMA), University of Aveiro, 3810-193 Aveiro, Portugal.

3) LASI - Intelligent Systems Associate Laboratory, Portugal.

§ Equally contributing authors

# These authors have jointly supervised the work

* Corresponding authors: Sandra I. Vieira (biomedicine; sivieira@ua.pt); Marco Santos (bioengineering; marco.santos@ua.pt)

Supplementary Figures 1-4

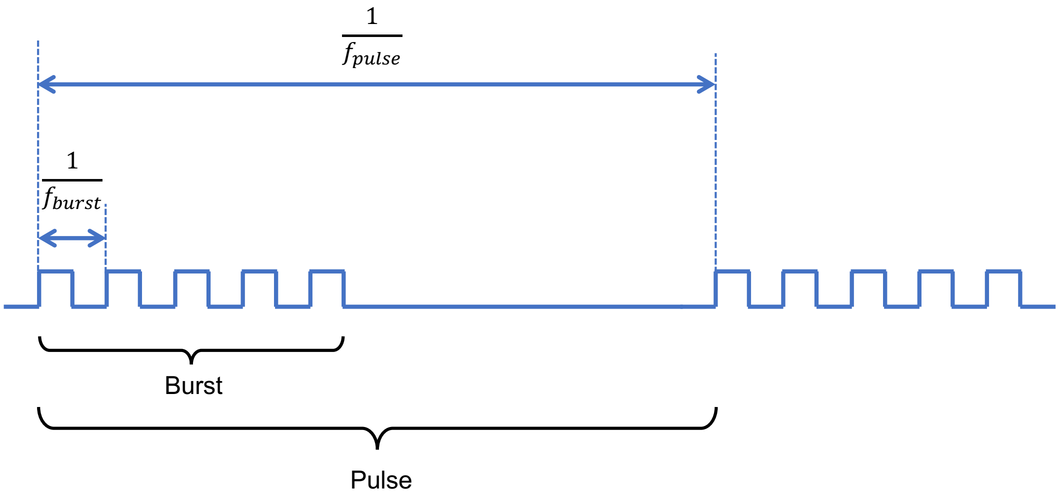

Supplementary Figure 1. Representation of the standardized signal characterization. f_{pulse} refers to the repeating element that composes the pulse and is used to calculate the pulse frequency. f_{burst} refers to the repeating element that composes the burst and is used to calculate the burst frequency.

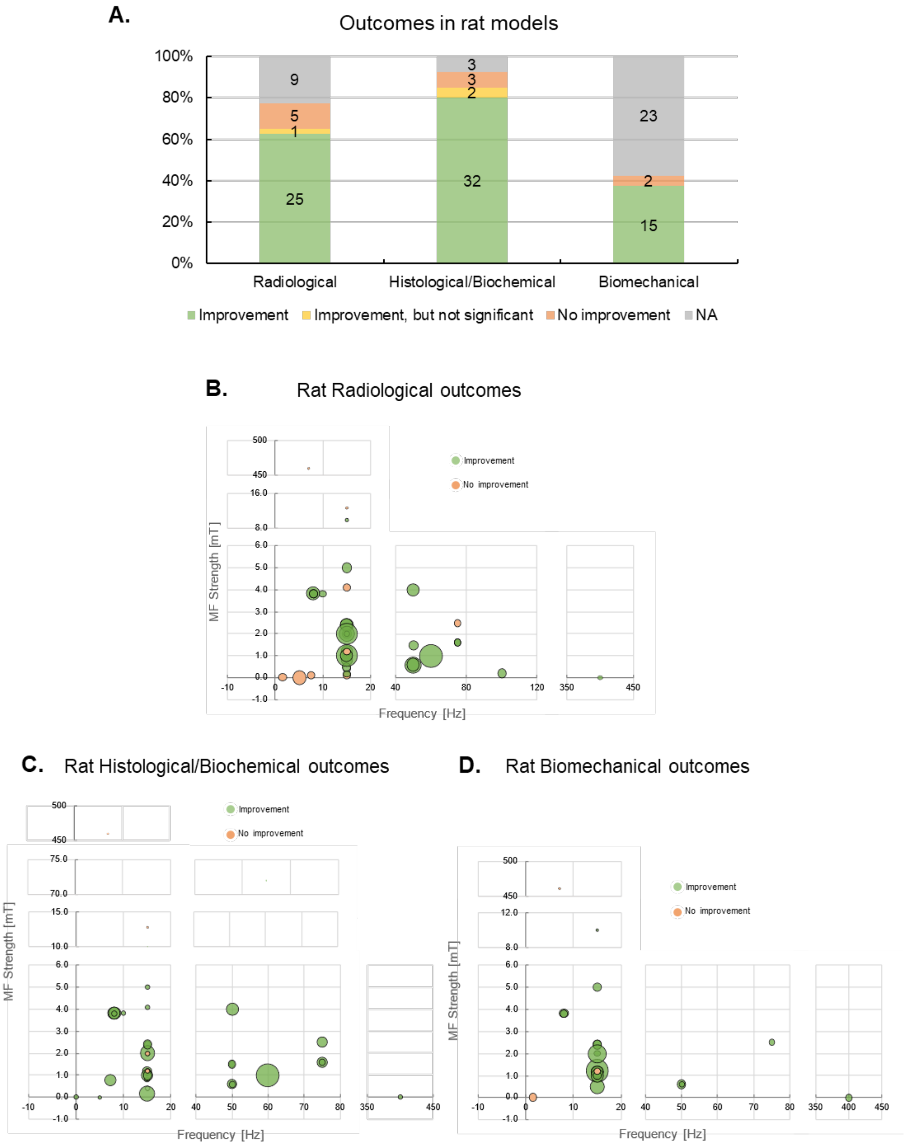

Supplementary Figure 2. Efficacy analysis of IC *in vivo* stimulation parameters (magnetic field and frequency) on rat models, based on the reported bone-associated outcomes. A. Overview of the IC efficacy on the three categories of outcomes (qualitative and quantitative details in Supplementary Tables 1 and 3, respectively). The number of studies is indicated in the graph. NA, not applicable (outcome not assessed). **B.** Radiological, **C.** Histological/biochemical and **D.** Biomechanical outcomes, according to the stimulus' frequency and MF strength used in each study. Each circle represents one study, and its diameter is directly proportional to the amplitude of the effect that IC stimulation had on that outcome category. Values of ± 0.1 and zero (0) were chosen to represent non-stated outcomes and unknown frequencies or MFs strengths, respectively.

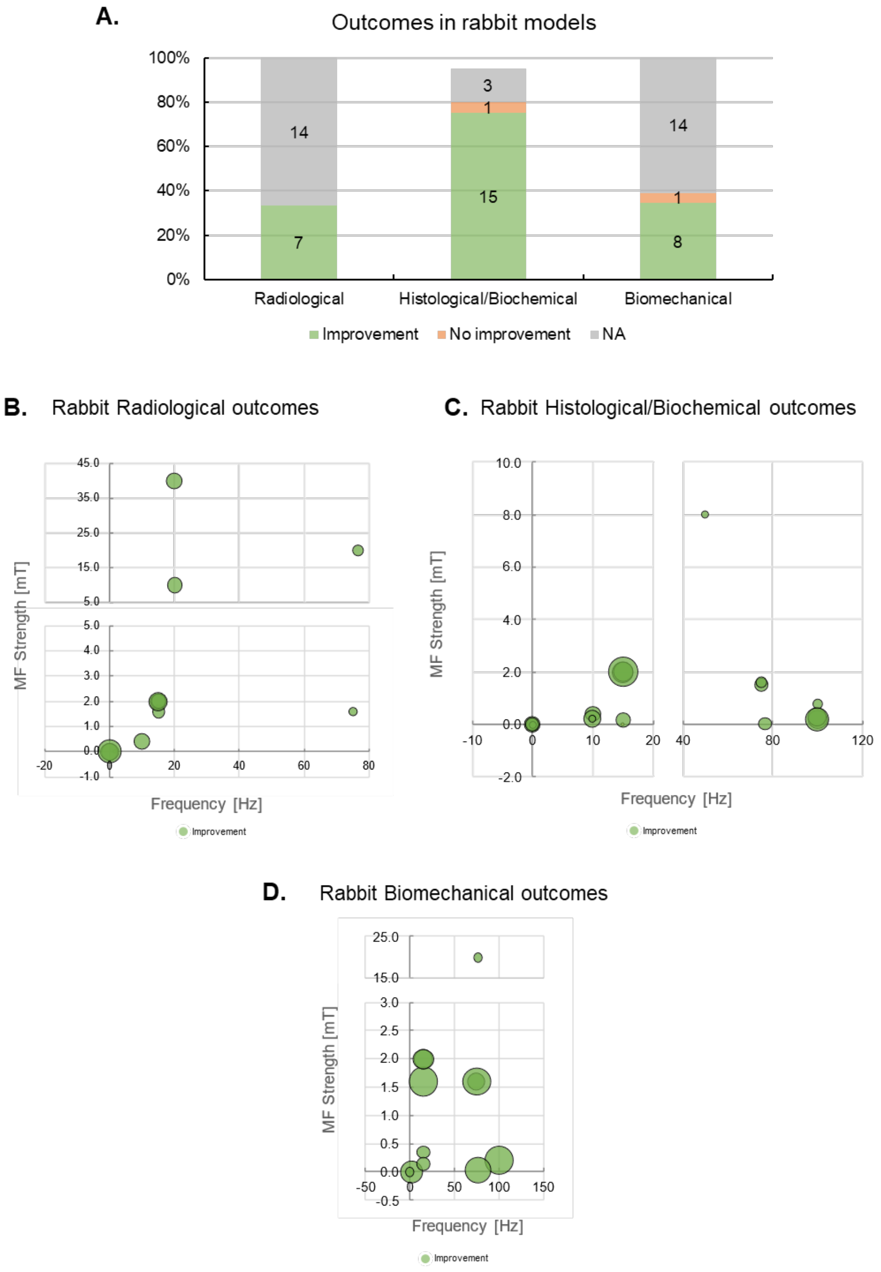

Supplementary Figure 3. Efficacy analysis of IC *in vivo* stimulation parameters (magnetic field and frequency) on rabbit models, with basis on the reported bone-associated outcomes. A. Overview of the IC efficacy on the three categories of outcomes (qualitative and quantitative details in Supplementary Tables 1 and 3, respectively). The number of studies is indicated in the graph. NA, not applicable (outcome not assessed). **B.** Radiological, **C.** Histological/biochemical and **D.** Biomechanical outcomes, according to the stimulus' frequency and MF strength used in each study. Each circle represents one study, and its diameter is directly proportional to the amplitude of the IC stimulation effect on that outcome category. Values of ± 0.1 and zero (0) were chosen to represent non-stated outcomes and unknown frequencies or MFs strengths, respectively.

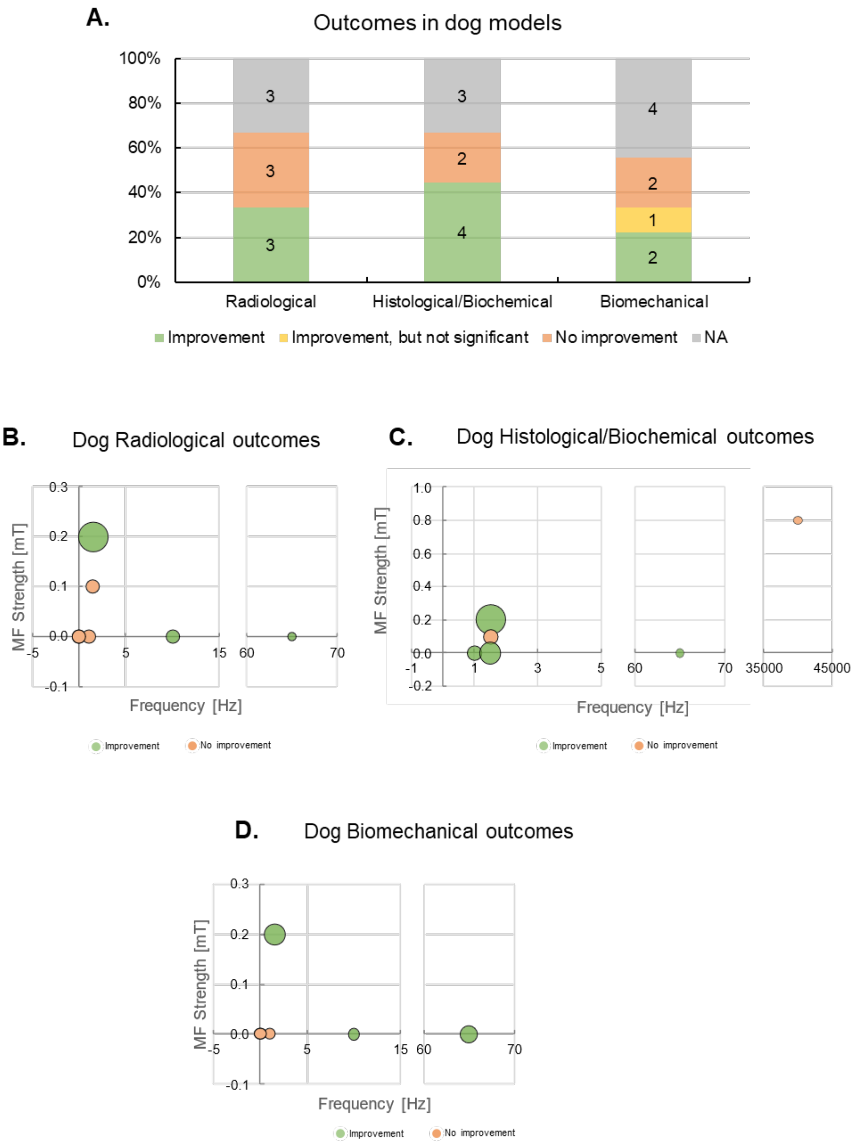

Supplementary Figure 4. Efficacy analysis of the IC in vivo stimulation parameters (magnetic field and frequency) on dog models, with basis on the reported bone-associated outcomes. A. Overview of the IC efficacy on the three categories of outcomes (qualitative and quantitative details in Supplementary Tables 1 and 3, respectively). The number of studies is indicated in the graph. NA, not applicable (outcome not assessed). **B.** Radiological, **C.** Histological/biochemical, and **D.** Biomechanical outcomes, according to the stimulus' frequency and MF strength used in each study. Each circle represents one study, and its diameter is directly proportional to the amplitude IC stimulation effect on that outcome category. Values of ± 0.1 and zero (0) were chosen to represent non-stated outcomes and unknown frequencies or MFs strengths, respectively.

Supplementary Tables 1-4

[Pre-clinical data: Suppl. Tables 1-2, pages 1-46; Clinical data, Suppl. Tables 3-4, pages 47-65]

Supplementary Table 1 - Inductive coupling stimulation characteristics and overall biological outcomes evaluated in animal models of musculoskeletal disorders. Data was collected from 66 studies and includes the evaluated musculoskeletal disorder/condition, the animal model characteristics (species, breed, and modification or procedures the animal was submitted), the stimuli characteristics (Type of stimulation, used device, stimuli waveform, frequency, periodicity, magnetic field strength, exposure time, and assay duration), and respective radiologic, histological or biochemical, and biomechanical outcomes. MF – magnetic field; AC – alternating voltage; CF - constant field; ES – electrical stimulation; BMD – bone mineral density; BV/TV – bone volume per total volume ration; Tb.Th – Trabecular thickness; Tb.N – Trabecular number; Tb.S – Trabecular separation; ALP – Alkaline phosphatase; OC – osteocalcin; Col I – collagen type I; FN – fibronectin; HA – hydroxyapatite; HU – hindlimb unloaded; OVX – Ovariectomized; P – phosphorus; PEMF – Pulsed Electromagnetic Field; CMF - Combined Magnetic Field; ND – not defined; NA – not applicable.

Ref	Condition	Animal model			Stimuli characteristics						Outcomes induced by IC stimulation		
		Species	Breed	Modification	Type of stimulation<br><br>Stimulation device<br><br>Waveform	Frequency (Hz)	Periodicity	MF strength (mT)	Exposure time (h/day)	Assay duration (weeks)	Radiologic	Histological and Biochemical	Biomechanical
1	*NA*	Rat – *Rattus norvegicus*	Wistar	Healthy rats	PEMF (Magstim™ model 220, TheMagstim Company Ltd, London, UK)	1	*ND*	30	0.5	3	*NA*	PEMF induced a significant thickness increase in cortical and trabecular *in vivo* stimulated bone tissues.	*NA*
2	*NA*	Rat – *Rattus norvegicus*	Wistar	Healthy rats	PEMF	50	50% duty ratio	0.6	1.5	8.7	PEMF treatment increased both femoral and vertebral BMD, and improved microarchitecture of cancellous bone.	PEMF significantly improved PINP levels (bone formation), but not CTX-I (bone resorption). PEMF induced changes in cAMP serum levels after 30 min of stimulation, returning to basal levels thereafter. PEMF increased expression levels of femur p-PKA and p-CREB, as well as soluble adenyl cyclase (sAC), indicating an osteogenic effect promoted by the sAC–cAMP–PKA–CREB signaling pathway in osteoblasts.	PEMF treatment increased both femoral and vertebral maximum loads, improving bone mechanical properties.

Ref	Condition	Animal model			Stimuli characteristics						Outcomes induced by IC stimulation		
		**Species**	**Breed**	**Modification**	**Type of stimulation**<br>**Stimulation device**<br>**Waveform**	**Frequency (Hz)**	**Periodicity**	**MF strength (mT)**	**Exposure time (h/day)**	**Assay duration (weeks)**	**Radiologic**	**Histological and Biochemical**	**Biomechanical**
3	Deficient endochondral ossification	Rat – *Rattus norvegicus*	Sprague Dawley	Confinement of immature rats in a box for movement restriction (deficient endochondral ossification model); decalcified bone matrix (DBM) powder implanted subcutaneously along the thoracic musculature	PEMF	15 (pulse)<br>4 444 (burst)	4.5 ms pulse bursts duration: 20 pulses sequence with 200 µs increasing phase, 20 µs decreasing phase, followed by a 5 µs pause	2	8	3	*NA*	In histomorphometric analyses, PEMF enhanced synthesis of cartilage molecules, and subsequent endochondral calcification. PEMF stimulation also promoted the maturation of bone trabeculae.	*NA*
4	Bone defect	Rat – *Rattus norvegicus*	Wistar	Pre-maxilla bone defect grafted with demineralized bone matrix (DBM)	PEMF; Quasi-rectangular pulses	15 (pulse)<br>100 (burst)	10 ms-wide pulse bursts: 100 µs wide pulses	0.15 – 0.18	12	5	More bone production with almost complete osseous bridging in the defect sites at D35	ALP activity increase from D7 to D14 and increased ^{45}Ca incorporation at D14	*NA*
5	Bone defect	Rat – *Rattus norvegicus*	Wistar	Femur incision with drill	PEMF (Physiotherapy system BTL-4000)	15	*ND*	1; 5; 10	2	1	Callus BMD increased more prominently after 5 and 10 mT PEMF therapy.	Compared to control, rats subjected to PEMF therapy had significantly higher levels of serum Ca and serum ALP at 5 and 10 mT, while the serum level of P remained unchanged.	Biomechanical measurements showed that 21 days after the therapy onset, the maximum load, fracture load, elastic load and bending energy were significantly greater in rats receiving 5 and 10 mT PEMF therapy.
6	Osteotomy	Rat – *Rattus norvegicus*	Sprague Dawley	Tibial osteotomies, stabilized with external fixators, distracted 0.375 mm twice daily for 6 days	PEMF (Orthofix Medical Inc., USA)	15	*ND*	1.2 (30 T/s converted to mT by comparison with the settings reported in Androjna *et al.*[25])	3	4	Significantly more bone volume and mineral density in the PEMF-treated group compared to the control group.	Histological results showed better quality callus formation, with less cartilage, in the PEMF-treated group.	Bones exposed to PEMF were biomechanically stronger.

Ref	Condition	Animal model			Stimuli characteristics						Outcomes induced by IC stimulation		
		Species	Breed	Modification	Type of stimulation<br><br>Stimulation device<br><br>Waveform	Frequency (Hz)	Periodicity	MF strength (mT)	Exposure time (h/day)	Assay duration (weeks)	Radiologic	Histological and Biochemical	Biomechanical
7	Bone Fracture	Rat – *Rattus norvegicus*	Sprague Dawley	Tibia fracture	PEMF	50 (pulse) 192 (burst)	10 ms pulse bursts: 0,5 ms pulse; 0,02 ms pulse interval	1.5	3.5	3	More callus formation, however, difference was not statistically significant.	Elevated serum OC levels and stereological tests also showed higher osteogenic results.	*NA*
8	Bone Fracture	Rat – *Rattus norvegicus*	Wistar	Femur fracture	PEMF	50	*ND*	1.5	6	4	*NA*	Larger increase in the cartilage and bone volumes for the PEMF and Pentoxifylline-treated groups than in control, and both treatments were equally effective on bone fracture healing.	*NA*
9	Bone defect	Rat – *Rattus norvegicus*	Sprague Dawley	Calvaria defect model to test recombinant human bone morphogenetic protein-2 (rhBMP-2) in bone regeneration	PEMF	60	12 µs pulses	1	8	0.7	PEMF stimulation alone accelerated bone regeneration, resulting in increased BV, BMD and TbTh. PEMF stimulation also increased TbN and decreased TbS when coupled with 2.5 µg rhBMP-2.	PEMF induced distinct bone regeneration of the bone defect and also more bone area and less dead space, compared with groups without stimulation. Furthermore, PEMF increased vWF (angiogenesis marker) and OPN (osteogenesis marker) expression in regenerated bone.	*NA*
10	Bone Fracture	Rat – *Rattus norvegicus*	Wistar	Femur fracture stabilized with titanium nails	HF-PEMF (Diapulse device)	400 (pulse); $27x10^6$ (burst)	400 pulses/sec; 65 µs pulse duration	*ND*	0.17	2	Higher BV/TV in the callus area.	Soft callus at a more advanced fibrocartilaginous stage and active bone marrow.	Higher mechanical strength of femurs.

Ref	Condition	Animal model			Stimuli characteristics						Outcomes induced by IC stimulation		
		Species	Breed	Modification	Type of stimulation<br>Stimulation device<br>Waveform	Frequency (Hz)	Periodicity	MF strength (mT)	Exposure time (h/day)	Assay duration (weeks)	Radiologic	Histological and Biochemical	Biomechanical
11	Non-union	Rat – *Rattus norvegicus*	Sprague Dawley	Fibular osteotomy Treatment 28 days after surgery	PEMF (Physio-Stim®, Orthofix Inc.); Triangular (sawtooth) pulses	15 (pulse); 3850 (burst)	5.56 ms pulse bursts duration and 67 ms burst on-off period	2	3	10	Reduction in the amount of time-dependent bone volume loss and smaller osteotomy gap size.	Reduction in the amount of time-dependent bone volume loss.	*NA*
12	Bone fracture	Rat – *Rattus norvegicus*	Sprague Dawley	Fibular osteotomy	PEMF (Physio-Stim®, Orthofix Inc.); Triangular (sawtooth) pulses vs PEMF (Osteo-Stim®, Orthofix Inc.)	15 (pulse); 3850 (burst) vs 1.5 (pulse); 63000 (burst)	5.56 ms pulse bursts duration and 67 ms burst on-off period vs 16 µs pulse burst duration and 670 ms burst on-off period	2.0 vs 0.02	3	3 or 5	Hard callus volumes and formation rate for Physio-Stim® consistently higher than controls. Osteo-Stim® treatment did not exhibit any effects on the longitudinal dynamics of hard callus volume and formation rate.	Physio-Stim® resulted in better cortical alignments, consolidation of the newly formed cancellous bony callus, and a better fill of the original osteotomy gap with new bone tissue (not achieved by Osteo-Stim® stimulation). Physio-Stim®-treated fibulae had mostly woven bone and marrow tissues with smaller amounts of hyaline cartilage, while Osteo-Stim®-treated fibulae contained mostly fibro-cartilage tissue with smaller amounts of hyaline cartilage, woven bone and marrow tissues.	Physio-Stim® increased relative bending strength. Osteo-Stim® treatment was not significantly different from control.
13	Bone Fracture	Rat – *Rattus norvegicus*	Sprague Dawley	Ulnar osteotomy	PEMF (PAP IMI®)	5	1 µs pulse duration	*ND*	0.08	7	Faster gradual bridging callus formation (healing process) in control group.	Higher fibrous content of the callus than the control group (less cartilage and bone; mechanical test needed).	*NA*

Ref	Condition	Animal model			Stimuli characteristics						Outcomes induced by IC stimulation		
		Species	Breed	Modification	Type of stimulation<br><br>Stimulation device<br><br>Waveform	Frequency (Hz)	Periodicity	MF strength (mT)	Exposure time (h/day)	Assay duration (weeks)	Radiologic	Histological and Biochemical	Biomechanical
14	Spine fusion	Rat – *Rattus norvegicus*	Sprague Dawley	Posterior vertebral arthrodesis in last 3 lumbar vertebrae	PEMF	*ND*	*ND*	*ND*	18	4 or 8	*NA*	Acceleration of bone callus organization after 4 weeks, and even more after 8. Apparent acceleration in bone callus growth, at least during early stages, and contribution to the transition from cartilage to bone.	*NA*
15	Soft Tissue Healing	Rat – *Rattus norvegicus*	Sprague Dawley	Rotator cuff tearing	PEMF (Physio-Stim®, Orthofix Inc.); Triangular (sawtooth) pulses	15 (pulse); 3850 (burst)	5.56 ms pulse bursts duration and 67 ms burst on-off period	2	3	4, 8 or 16	At 4 weeks under PEMF stimulation, trabecular thickness significantly decreased and connectivity density was significantly increased. At 16 weeks under PEMF stimulation, significantly increased bone volume fraction, trabecular thickness, and BMD, and a trend toward increased bone mineral content.	At the injury site, no differences were detected in cell shape and cellularity and collagen fiber organization at any time point. In the midsubstance, the PEMF group had significantly more rounded cells.	At 4 weeks, PEMF induced increased tendon modulus and stress, that ultimately did not alter joint function.
16	Soft Tissue Healing	Rat – *Rattus norvegicus*	Sprague Dawley	Rotator cuff tearing	PEMF (Physio-Stim®, Orthofix Inc.); Triangular (sawtooth) pulses<br>vs<br>HF-PEMF	15 (pulse); 3800 (burst)<br>vs<br>15 (pulse); 40850 (burst)	*ND*	1.19	1, 3, or 6	4, 8 and 16	*NA*	Improvement in Col I and FN tendon expression, upon 4 weeks of treatment. No significant differences were measured in cellularity. Some differences were noted for collagen fiber alignment.	Improvements in different mechanical properties across all time points, specially increases in tendon modulus and stiffness as early as 4 weeks with 1-hour of Physio-Stim.
17	Soft Tissue Healing	Rat – *Rattus norvegicus*	Sprague Dawley	Transection and modified Kessler repair of the Achilles' tendon + 1 week limb immobilization.	PEMF (Physio-Stim®, Orthofix Inc.); Triangular (sawtooth) pulses	15 (pulse); 3850 (burst)	*ND*	1.19	1 or 3	1, 3 or 6	No differences between groups in bone volume or BMD of heterotopic ossification at any time point.	In complete tearing, cellularity increased at 1-week with 1-hour PEMF. Cell shape was more rounded in PEMF groups compared to controls.<br>In partial tearing with immobilization, cellularity decreased at 3 weeks with 1-hour PEMF. No differences were seen in collagen alignment.	Some decreases in tendon mechanical properties and ankle function suggest PEMF may be detrimental after complete tear.

Ref	Condition	Animal model			Stimuli characteristics						Outcomes induced by IC stimulation		
		Species	Breed	Modification	Type of stimulation<br>Stimulation device<br>Waveform	Frequency (Hz)	Periodicity	MF strength (mT)	Exposure time (h/day)	Assay duration (weeks)	Radiologic	Histological and Biochemical	Biomechanical
18	Osteoporosis	Rat – *Rattus norvegicus*	Sprague Dawley	OVX	Pulsed burst PEMF (Hunan Forever Elegance Technology)	8 (pulse); 4000 (burst)	25 ms pulsed burst and 100 ms interval; 0.2 ms pulses and 0.05 ms interval	3.82	0.67	12	Improvement in BMD and bone microarchitecture	Lower levels of serum TRACP5b.<br>Higher OPG mRNA expressions while lower RANKL mRNA expression in L3 vertebral bodies, femur and tibia.	Improvement in strength of the lumbar spine
19	Osteoporosis	Rat – *Rattus norvegicus*	Sprague Dawley	OVX	Pulsed burst PEMF (Hunan Forever Elegance Technology)	8 (pulse); 4000 (burst)	25 ms pulsed burst and 100 ms interval; 0.2 ms pulses and 0.05 ms interval	3.82	0.67	12	Early PEMF prevented ovariectomy-induced deterioration in BMD and deterioration in bone micro-architecture of lumbar vertebral bodies, femurs and tibias. Late PEMF only prevented deterioration in lumbar vertebral bodies.	No significant changes in RANK mRNA expression and lower RANK protein expression compared to control.	Early PEMF prevented deterioration of mechanical properties of lumbar vertebral bodies and femur. Late PEMF only prevented mechanical deterioration in lumbar vertebral bodies.
20	Osteoporosis	Rat – *Rattus norvegicus*	Wistar	OVX	PEMF (Orthopulse®) Vs PEMF+5 min on/off or +50-150 kHz noise, or w/ 7.5 Hz	15 (pulse); 200 000 (burst) vs 7.5 (pulse)	5-ms pulse bursts with 5-μs pulses	0.1	2	6	No differences between PEMF groups with the non-stimulated group in microCT measurements (BV/TV; Tb.Th; Conn.D; SMI; Ct.V; Ct.Th)	*NA*	*NA*
21	Osteoporosis	Rat – *Rattus norvegicus*	Sprague Dawley	OVX; Fibular fracture	PEMF; Waveform made to resemble a widely used lineal waveform	15 (pulse); 3850 (burst)	*ND*	0.5	3	6	Improved bridging across fibular fractures.	*NA*	Improvement of hard callus elastic modulus.

Ref	Condition	Animal model			Stimuli characteristics						Outcomes induced by IC stimulation		
		Species	Breed	Modification	Type of stimulation<br><br>Stimulation device<br><br>Waveform	Frequency (Hz)	Periodicity	MF strength (mT)	Exposure time (h/day)	Assay duration (weeks)	Radiologic	Histological and Biochemical	Biomechanical
22	Osteoporosis	Rat – *Rattus norvegicus*	Wistar	Periodontitis (P); Periodontitis + PEMF (P + PEMF); Ovariectomy + Periodontitis (P + OVX); Ovariectomy + Periodontitis + PEMF (P + OVX + PEMF). PEMF therapy began 7 days after periodontitis induction.	PEMF (custom-made);	15	200 µs pulse width—50 V during 60 ms for a total of 25 cycles that repeat at the rate of 15 Hz	1	3	3	PEMF reverted some of the detrimental effects on bone microarchitecture of periodontitis (BMD and Tb.N) and osteoporosis (Tb.N)	PEMF attenuated bone loss. PEMF reduced IL-1β, IL-6, and TNF-α, reverting the OVX effects, as well as variations in IL-10 and VEGF. PEMF also increased the OPG/RANKL ratio in comparison with the OVX groups.	*NA*
23	Osteoporosis	Rat – *Rattus norvegicus*	Sprague Dawley	OVX	Pulsed burst PEMF	15 (pulse); 5000 (burst)	5 ms pulse bursts width and 60 ms wait; 0.2 ms pulse width; 0.02 ms pulse wait; 0.3 µs pulse rise; 2.0 µs pulse fall	2.4	8	10	PEMF improved bone mass and bone architecture, evidenced by increased BMD, Tb.N, Tb.Th and BV/TV, and lower Tb.S and SMI.	PEMF exposure significantly upregulated overall gene expressions of Wnt1, LRP5 and β-catenin in the canonical Wnt signalling, but differences were not significant on either RANKL or RANK.	PEMF attenuated the biomechanical strength deterioration of OVX, evidenced by increased maximum load and elastic modulus. Bone stiffness and energy absorption were not significantly different.
24	Osteoporosis	Mouse – *Mus musculus*	C57BL/6	OVX	PEMF; Square wave	15	*ND*	2.4 – 2.6	1	8	Micro-CT analysis showed that PEMF administration substantially countered OVX-induced bone loss as shown by greater Tb.N, Tb.Th, BMD and BV/TV and less Tb.S	Immunohistochemistry further proves the micro-CT findings. PEMF stimulation increased serum biomarkers of bone formation, including ALP and OC. Likewise, the bone resorption biomarker CTX-I and TRACP5b levels were more reduced. PEMF increased expression of Osterix transcripts.	*NA*

Ref	Condition	Animal model			Stimuli characteristics						Outcomes induced by IC stimulation		
		Species	Breed	Modification	Type of stimulation<br><br>Stimulation device<br><br>Waveform	Frequency (Hz)	Periodicity	MF strength (mT)	Exposure time (h/day)	Assay duration (weeks)	Radiologic	Histological and Biochemical	Biomechanical
25	Osteoporosis	Rat – *Rattus norvegicus*	Sprague Dawley	OVX	PEMF; sinusoid pulses	15 (pulse); 3850 (burst)	*ND*	0.41, 1.2, 4.1, 12.4	3	6	1.2 mT PEMF approached the efficacy of alendronate, a drug for osteoporosis, in reducing trabecular bone loss (microarchitecture)	1.2 and 4.1 mT PEMF treatments improved the lacunocanalicular features in cortical bone, consistent with osteocyte sensitivity to PEMF *in vivo*. Histologically, PEMF increased bone formation rate per bone surface (BFR/BS) compared to untreated OVX and alendronate.	*NA*
26	Osteoporosis	Rat – *Rattus norvegicus*	Sprague Dawley	Hindlimb unloading (HU) model via tail suspension	PEMF; Repetitive single square wave pulse	10	*ND*	3.82	0.67	12	At 12 weeks, PEMF increased both Tb.Area and Tb.N, whereas the Tb.S was reduced, compared with the HU group. Tb.Th increased slightly but the difference was not significant.	Serum OC concentration increased significantly compared with the control group.	*NA*
27	Osteoporosis	Rat – *Rattus norvegicus*	Sprague Dawley	Hindlimb unloading (HU) model via tail suspension	PEMF	15 (pulse); 5000 (burst)	5 ms pulse bursts width and 60 ms wait; 0.2 ms pulse width; 0.02 ms pulse wait; 0.3 μs pulse rise; 2.0 μs pulse fall	2.4	2	4	PEMF exposure attenuated disuse-induced decrease of bone mass and deterioration of bone microarchitecture.	PEMF stimulation significantly promoted osteoblastogenesis and exerted only a minor inhibitory action on osteoclastogenesis and increased mineral apposition and bone formation rates. Also, it increased serum levels of bone formations markers, while decreasing bone resorption markers. These changes are presumed to be promoted by the Wnt signaling pathway.	PEMF attenuated disuse-induced decrease of bone mechanical strength.

Ref	Condition	Animal model			Stimuli characteristics						Outcomes induced by IC stimulation		
		Species	Breed	Modification	Type of stimulation<br>Stimulation device<br>Waveform	Frequency (Hz)	Periodicity	MF strength (mT)	Exposure time (h/day)	Assay duration (weeks)	Radiologic	Histological and Biochemical	Biomechanical
28	Osteoporosis	Rat – *Rattus norvegicus*	Sprague Dawley	Hindlimb unloading (HU) model via tail suspension	PEMF; Repetitive single square wave pulse	15	8 ms pulse duration	0.8	2	8	Increased BMD	Increased serum TGF-β1 concentration and significantly lowered IL-6 levels after 8 weeks of treatment.<br>Efficient bone mass loss suppression and bone remodelling modulation.	*NA*
28	Osteoporosis	Rat – *Rattus norvegicus*	Wistar	Hindlimb unloading (HU) model via tail suspension	PEMF	50	50% duty cycle	0.6	1.5	4	PEMF treatment prevented the BMD loss (femoral and vertebral) by about 50%, preserved the microstructure of cancellous bone and thickness of cortical bone.	PEMF significantly prevented osteoblast reduction and inhibited increases in adipocytes in the bone marrow, blocked decreases in PTH serum levels and its downstream signal molecule cAMP. PEMF maintained the phosphorylation levels of protein kinase A (PKA) and cAMP response element-binding protein (CREB), also maintaining the expression level of soluble adenylyl cyclase (sAC). PEMF prevented decreases in serum levels of bone formation markers and increases in bone resorption markers.	PEMF stimulation prevented bone maximum load loss by about 50%.
30	Osteoporosis	Rat – *Rattus norvegicus*	Sprague Dawley	Hindlimb unloading (HU) model via tail suspension	Rotating magnetic field (RMF)	7	*ND*	380 – 600	2	4	No significant difference was observed in trabecular or cortical bone parameter between the HU and HU+RMF groups	No significant difference in serum OC, P1NP (bone formation), TRACP5b or CTX-I (bone resorption) levels was found between the HU and HU+RMF groups	RMF exposure for 4 weeks exerted no significant impacts on maximum load, stiffness, energy absorption or elastic modulus in HU rats

Ref	Condition	Animal model			Stimuli characteristics						Outcomes induced by IC stimulation		
		Species	Breed	Modification	Type of stimulation<br>Stimulation device<br>Waveform	Frequency (Hz)	Periodicity	MF strength (mT)	Exposure time (h/day)	Assay duration (weeks)	Radiologic	Histological and Biochemical	Biomechanical
31	Osteoporosis	Rat – *Rattus norvegicus*	Sprague Dawley	5 weeks heparin treatment	PEMF	7.3	*ND*	0.8 ± 0.2	1	4	*NA*	PEMF increased the amount of new bone area and lowered C-telopeptides on Day 63, on rats with heparin-induced osteoporosis.	*NA*
32	Osteoporosis	Rat – *Rattus norvegicus*	Sprague Dawley	Glucocorticoid treatment (dexamethasone)	PEMF; Square wave	50	200 μs pulse width	4	0.67	12	BMD of the whole body increased significantly and trabeculae were thicker after PEMF stimulation.	PEMF group had upregulated mRNA and protein expression of Wnt10b, LRP5, β-catenin, OPG, and Runx2 and downregulated Axin2, PPAR-γ, C/EBPα, FABP4, and Dkk-1. These results suggest that PEMF stimulation can prevent bone loss.	*NA*
33	Osteoporosis	Rat – *Rattus norvegicus*	Sprague Dawley	Aged rats (24 months old) (senile osteoporosis model)	PEMF (Hunan Forever Elegance Technology, China)	8 (pulse); 4000 (burst)	25 ms pulsed burst and 100 ms interval; 0.2 ms pulses and 0.05 ms interval	3.82	0.67	12 (5days/week)	Bone density (assessed by DEXA) and microstructure (assessed by μCT) in the aged rats treated with PEMF were significantly better than those in the aged control group. PEMF partially reversed bone deterioration related to aging.	Serum markers (bone-specific ALP and TRACP5b) and gene expression (Wnt/β-catenin pathway) related to bone turnover, bone loss and microarchitecture deterioration in the aged rats treated with PEMF were significantly better than those in the aged control group. PEMF can regulate genes and differentiation of bone marrow stem cells, altered with aging, to favor bone formation and prevent bone loss.	*NA*
34	Implant	Rat – *Rattus norvegicus*	Wistar	Tibial titanium implant	PEMF	15	25 cycles in 200 μs	±1.0	1 or 3	5 days/week (up to 45 days)	1h exposure showed better results in bone volume and BMD.<br>3h exposure showed better results in trabecular bone thickness.	1h exposure showed better results in cell viability, total protein content, and mineralization nodules.<br>3h exposure showed better results in cell proliferation, especially at osseointegration in early periods.	1h exposure showed better results in removal torque tests.

Ref	Condition	Animal model			Stimuli characteristics						Outcomes induced by IC stimulation		
		Species	Breed	Modification	Type of stimulation<br><br>Stimulation device<br><br>Waveform	Frequency (Hz)	Periodicity	MF strength (mT)	Exposure time (h/day)	Assay duration (weeks)	Radiologic	Histological and Biochemical	Biomechanical
35	Implant and fracture (Abstract only)	Rat – *Rattus norvegicus*	Wistar	Titanium implants in right tibial crest and osteotomy in left tibial crest	PEMF	50	*ND*	72	0.5 (2x a day)	5, 10 or 20 days	*NA*	Increased peri-implant ossification and osteotomy ossification. Acceleration of both bone-healing and peri-implant bone formation.	*NA*
36	Implant	Rat – *Rattus norvegicus*	Fisher Inbred	Titanium alloy pins implanted intramedullary in distal femurs followed by the injection of polyethylene particles to induce osteolysis	PEMF (Biostim, IGEA, Carpi, Italy)	75	1.3 ms	2.5	6	8.6 (60 days)	Changes could not be seen in microarchitecture measured through micro-CT between the untreated and PEMF stimulated group.	PEMF increased trabecular bone volume with less cortical bone width, increased bone-to-implant contact, while reducing fibrous capsule around implants and bone, and osteoclast number.	PEMF stimulation increased bone microhardness around implants but did not have a significant increase in bone maturation index compared to the unstimulated group.
37	Implant	Rat – *Rattus norvegicus*	Sprague Dawley	12-week-old; OVX; titanium implants in proximal metaphysis of tibia	PEMF	100	25 μs pulse duration	0.2	4	2	Higher peri-implant bone volume and trabecular-number around implants. Trabecular thickness not significant.	*NA*	*NA*
38	Osteoarthritis	Rat – *Rattus norvegicus*	Sprague Dawley	Unilateral anterior crossbite group (UAC) to model temporomandibular joint osteoarthritis (TMJOA)	PEMF; Pulsed burst	15 (pulse); 5000 (burst)	5 ms pulse bursts width and 60 ms wait; 0.2 ms pulse width; 0.02 ms pulse wait; 0.3 μs pulse rise; 2.0 μs pulse fall	2	2	3 or 6	PEMF reversed some of the microarchitecture deterioration (BV/TV, BS/BV, Tb.Th, Tb.Sp) induced by UAC, at 6 weeks.	PEMF reversed the increased osteoclast activity and expression of osteoclast-related factors (RANKL) and down-regulated expression of osteogenesis-related factors (OPG, ALP, Runx2 and OCN) induced by UAC at the early stages of this experiment.	*NA*

Ref	Condition	Animal model			Stimuli characteristics						Outcomes induced by IC stimulation		
		Species	Breed	Modification	Type of stimulation<br><br>Stimulation device<br><br>Waveform	Frequency (Hz)	Periodicity	MF strength (mT)	Exposure time (h/day)	Assay duration (weeks)	Radiologic	Histological and Biochemical	Biomechanical
39	Osteoarthritis	Rat – *Rattus norvegicus*	Sprague Dawley	Unilateral anterior crossbite group (UAC) to model temporomandibular joint osteoarthritis (TMJOA)	PEMF; Pulsed burst	15 (pulse); 5000 (burst)	5 ms burst width; 0.2 ms pulse width; 0.02 ms pulse wait; 60 ms burst wait; 0.3 ms pulse rise; 2.0 ms pulse fall	2	2	3 or 6	*NA*	PEMF reversed the decreased cartilage thickness and degraded cartilage extracellular matrix. It also partially inhibited the up regulation of pro-inflammatory, degradative, and hypertrophic factors ((TNF-α, IL-1β, MMP-13, ADAMTS-5, IL-6, MMP-3, MMP-9 and COL-X). The synovium hyperplasia was inhibited as well.	*NA*
40	Osteoarthritis	Rat – *Rattus norvegicus*	Sprague Dawley	Treated with low-dose monosodium iodoacetate (MIA) to induce knee OA	PEMF	75	10% duty ratio; 1.33 ms pulse width; 12 ms pulse wait	1.6	2	4	Preservation of subchondral trabecular bone microarchitecture and prevention of subchondral bone loss	Increase in bone and cartilage formation, decrease in bone and cartilage resorption (from serum OC, serum N-propeptide IIA of type II collagen (PIIANP), urine C-terminal telopeptide of collagen type I (CTX-I), and urine C-terminal telopeptide of collagen type II (CTX-II))	*NA*
41	Osteoarthritis	Rat – *Rattus norvegicus*	Sprague Dawley	Treated with low dose monosodium iodoacetate (MIA) to induce knee OA	PEMF	75	10% duty ratio; 1.33 ms pulse width; 12 ms pulse wait	1.6	2	4	PEMF stimulation significantly increased levels of BV/TV, Tb.Th, and Tb.N in some tibia and femur compartments, and decreased levels of BS/BV in lateral compartments of femur and Tb.S in all tibia and femur compartments as compared with the OA group.	PEMF stimulation significantly increased tibial subchondral bone's Wnt3a, β-catenin, and OPG mRNA expressions, but increases on LRP5 and RANKL mRNA expressions were not significative. Immunohistochemistry (IHC) analysis show further evidence of these findings, and LRP5 was significantly increased. Differences in RANKL were still not visible by IHC.	*NA*

Ref	Condition	Animal model			Stimuli characteristics						Outcomes induced by IC stimulation		
		Species	Breed	Modification	Type of stimulation<br><br>Stimulation device<br><br>Waveform	Frequency (Hz)	Periodicity	MF strength (mT)	Exposure time (h/day)	Assay duration (weeks)	Radiologic	Histological and Biochemical	Biomechanical
42	Osteoarthritis	Guinea Pig – *Cavia porcellus*	Dunkin Hartley	Spontaneously developed degenerative joint diseases (knee OA)	PEMF	75	duty cycle of 1.3 ms	1.6	6	13	No significant differences observed in epiphyseal trabecular bone remodelling processes between control and stimulated groups.	PEMF stimulation significantly reduced chondropathy progression in all knee examined areas, by increasing cartilage thickness while lowering cartilage histological score, fibrillation index and subchondral bone thickness.	*NA*
43	Osteoarthritis	Guinea Pig – *Cavia porcellus*	Dunkin Hartley	Spontaneously developed degenerative joint diseases (knee OA); late knee OA stage	PEMF	37 or 75	1.3 ms	1.5	6	12	Both frequencies significantly reduced Tb.N and increased Tb.Th and Tb.S.	Both frequencies significantly reduced histological cartilage score, fibrillation index (FI), subchondral bone thickness (SBT).<br>75 Hz PEMF produced more beneficial effects on histological score and FI, and higher cartilage thickness.	*NA*
44	Bone fracture	Rabbit – *Oryctolagus cuniculus*	New Zealand White	Osteotomy (tibia mid-shaft) stabilized with external fixators, distracted 0.25 mm twice daily for 21 days	time-varying PEMF with asymmetric EFs	1.5	30 ms pulso bursts	*ND*	1	9, 16, and 23 days	Significantly more callus generated (D9-D16). By 21 days, PEMF-treated tibiae showed bridging callus on both cortices, whereas most of the sham controls still had lucent fracture lines	*NA*	Stronger tibiae. By 16 days post-distraction, biomechanical strength essentially equivalent to intact bone.
45	Osteotomy (Abstract only)	Rabbit – *Oryctolagus cuniculus*	New Zealand White	Fibular osteotomy	PEMF; Symmetric quadrangular waveform; Different pulse amplitudes (10; 25; 50; 75; 100; 125; 200 mV)	15 (pulse)	**Amplitude test:**<br>5 (10 – 125 mV) and 10 (200 mV) μs pulse-width;<br><br>**Pulse width test (for 100 mV):**<br>0.5, 1, 2, 3, 4, 5 and 7 μs pulse-width;<br><br>5 ms pulsed bursts; 62 ms interval	**Amplitude test:**<br>28, 71, 141, 212, 283, 353 and 1130 μT<br>**Pulse width test (for 100 mV):**<br>28, 57, 113, 170, 226, 283, 395 μT	24	16 days	*NA*	*NA*	Significant increase of callus bending stiffness produced by pulse widths of 141 and 395 μT and pulse amplitudes of 50 to 100 mV, respectively.

Ref	Condition	Animal model			Stimuli characteristics						Outcomes induced by IC stimulation		
		**Species**	**Breed**	**Modification**	**Type of stimulation<br><br>Stimulation device<br><br>Waveform**	**Frequency (Hz)**	**Periodicity**	**MF strength (mT)**	**Exposure time (h/day)**	**Assay duration (weeks)**	**Radiologic**	**Histological and Biochemical**	**Biomechanical**
46	Osteotomy	Rabbit – *Oryctolagus cuniculus*	New Zealand White	Tibial osteotomy	PEMF<br>Vs<br>CMF<br>(Sine wave + static field)	PEMF – 15<br>CMF – 76.6	PEMF – 4.5 ms duration pulse bursts of 20 pulses	PEMF – up to 1.6 mT<br>CMF1 – 20 mT amplitude<br>CMF2 – 20 mT	0.5, 3 or 6	2 or 3	Increases in mean periosteal callus at 14 days when PEMF and CMFs were used for 3 and 6 h/days. At 21 days, increase in mean callus areas at all three daily exposure times.	*NA*	At 14 days, increase in torsional strength in the 3 and 6 hour-treated groups, significant only for the 6-hour groups. At 21 days, stronger bone on all exposure treatments.<br>6-hour exposure was more effective.
47	Bone defect	Rabbit – *Oryctolagus cuniculus*	New Zealand White	Femoral condylar defects (diameter 5 mm; depth 6 mm); intramedullary implants	PEMF<br>Vs<br>CMF;<br>(intramedullary implant)	PEMF – 20<br>CMF – 20	*ND*	PEMF – 10<br>CMF – up to 40 ± 5 mT	1	5	Better new bone volume and trabecular structure in the bone defect area. These effects were higher under CMFs than PEMF.	More new bone formed in the medullary cavity and more hypertrophic chondrocytes and new woven bone formation.	*NA*
48	Bone defect	Rabbit – *Oryctolagus cuniculus*	New Zealand White	Lateral knee arthrotomy in the loading area of both medial femoral condyles. One defect filled with a scaffold of heterologous equine collagen-I, and the other with the scaffold seeded with bone marrow concentrate (BMC).	PEMF (I-ONE, Igea SpA, Modena-Italy)	75	*ND*	1.5	4	6	*NA*	PEMF-stimulated group had total modified O'Driscoll score significantly higher than no stimulation, with low hypocellularity and better tissue morphology and bone microarchitecture parameters, except for the bone reconstruction parameters. Also, in the macroscopic evaluation the defect was mostly filled up but presented irregularities in the "well-integrated and barely noticeable surface".	*NA*

Ref	Condition	Animal model			Stimuli characteristics						Outcomes induced by IC stimulation		
		Species	Breed	Modification	Type of stimulation<br>Stimulation device<br>Waveform	Frequency (Hz)	Periodicity	MF strength (mT)	Exposure time (h/day)	Assay duration (weeks)	Radiologic	Histological and Biochemical	Biomechanical
49	Implant	Rabbit – *Oryctolagus cuniculus*	Japanese White	Rough-surfaced dental implant in femurs	PEMF	100	25 µs pulse duration	0.2; 0.3; 0.8	4 or 8	1, 2 and 4	*NA*	Bone-implant contact ratio and bone area ratio with 0.2 and 0.3 mT larger than 0.8 mT.<br>Greater amount of bone formed around the implant of the 2-week-treated femurs than the 1-week-treated femurs, and not significantly different from 4-week-treated femurs.<br>No difference between 4 h/day and 8 h/day and treatment for 2 and 4 weeks.	*NA*
50	Implant	Rabbit – *Oryctolagus cuniculus*	New Zealand White	Custom Ti-6Al-4V dental implants in mandible	PEMF	100	25 µs pulse duration	0.2	4	2	*NA*	PEMF stimulation increased osteoblast number and trabecular bone at 2 weeks and significantly upon 6 weeks of non-stimulation, thus it may positively affect the bone healing process after implant placement.	*NA*
51	Implant	Rabbit – *Oryctolagus cuniculus*	New Zealand White	Titanium implants in proximal metaphyses of tibia	PEMF	10	*ND*	0.2 to 0.4	24	2 and 4	Better bone microarchitecture, but no difference in trabecular thickness.	Higher bone-implant contact (BIC) and mature trabecular and woven bone observed in direct contact with the implant surface with no gaps or connective tissue at the bone-implant interface.	*NA*

Ref	Condition	Animal model			Stimuli characteristics						Outcomes induced by IC stimulation		
		Species	Breed	Modification	Type of stimulation<br>Stimulation device<br>Waveform	Frequency (Hz)	Periodicity	MF strength (mT)	Exposure time (h/day)	Assay duration (weeks)	Radiologic	Histological and Biochemical	Biomechanical
52	Implant	Rabbit – *Oryctolagus cuniculus*	New Zealand White	Intramedullary Kirshner wire inserted in the humerus	PEMF; Repetitive single square wave pulse	10	25 µs pulse duration	0.2	12	2	*NA*	PEMF on Kirshner wires increased ALP activity and osteoblast proliferative activity in the bone marrow 7 days after surgery and even further at 14 days after surgery, compared with Kirshner wire alone or PEMF alone. These findings revealed that the degree of osteogenesis induced by electromagnetic stimulation is influenced by tissue environment, and that osteogenesis is promoted markedly by inflammation and presence of reactive cells.	*NA*
53	Implant (Abstract only)	Rabbit – *Oryctolagus cuniculus*	Japanese White	Porous bead-covered titanium implants inserted into the humerus diaphysis	PEMF	10	25 µs pulse duration	0.2	*ND*	2	*NA*	PEMF promoted bone ingrowth into the porous implant.	*NA*
54	Implant	Rabbit – *Oryctolagus cuniculus*	New Zealand White	Intramedullary Kirshner wire implant in the medullary canal of femur and tibia	PEMF (American Medical Electronics, Dallas, TX, U.S.A.); Quasi-square (trapezoidal) pulses	15 (pulse); 4000 (burst)	5 ms pulse bursts: 35 µs burst, 220 µs wait	*ND*	4	3	*NA*	PEMF-treated movable implants in the femur induced +1.44-fold new bone formation, and a +21.5% enlargement of the area of the medullary canal, compared to untreated movable implants. In the tibia, these effects were not observed. PEMF did not induce bone at stationary implants, suggesting that it is insufficient to induce bone formation without other stimuli. There was no adverse effect with PEMF stimulation.	*NA*

Ref	Condition	Animal model			Stimuli characteristics						Outcomes induced by IC stimulation		
		Species	Breed	Modification	Type of stimulation<br><br>Stimulation device<br><br>Waveform	Frequency (Hz)	Periodicity	MF strength (mT)	Exposure time (h/day)	Assay duration (weeks)	Radiologic	Histological and Biochemical	Biomechanical
55	Implant	Rabbit – *Oryctolagus cuniculus*	New Zealand White	Femoral porous titanium (pTi) implants	Pulsed burst PEMF	15 (pulse); 5000 (burst)	5 ms pulse bursts width and 60 ms wait; 0.2 ms pulse width; 0.02 ms pulse wait; 0.3 µs pulse rise; 2.0 µs pulse fall	2	2	6 and 12	PEMF stimulation increased BV/TV, Tb.N, and Tb.Th, while BS/BV and Tb.S were significantly lower.	PEMF promoted osteogenesis, bone ingrowth and bone formation rate around pTi implants on rabbit femoral bone defects, while promoting gene expressions of Runx2, BMP2, OC and Wnt/β-catenin signalling.	*NA*
56	Implant	Rabbit – *Oryctolagus cuniculus*	New Zealand White	Tricalcium phosphate (TCP) or HA rod implants in tibia	PEMF (American Medical Electronics, Dallas, TX, U.S.A.); Quasi-square (trapezoidal) pulses	1.5 (pulse); 3846 (burst)	26 ms pulsed burst width; 100 burst pulses of 260 µs width;	0.18	8	1, 2, 3, 4, 6	*NA*	PEMF-stimulated group had significantly greater amount of bone and thicker bone trabeculae in the HA pores compared control group upon 3-4 weeks of implantation. No significant differences for these parameters in the TCP pores. Histologically, PEMF-treated animals had more bone and wider bone trabeculae in the HA implants, but not in TCP, at the early time periods when compared with control.	*NA*
57	Implant	Rabbit – *Oryctolagus cuniculus*	New Zealand White	Natural or synthetic HA rod implants in tibia	PEMF; Triangular pulse	50	2 ms pause between pulses	8	0.5x2	2 or 4	*NA*	PEMF-treatment resulted in acceleration of bone formation and HA integration at early time periods.	*NA*
58	Implant	Rabbit – *Oryctolagus cuniculus*	New Zealand White	Hydroxyapatite implant in femoral condyles	PEMF (Biostim, IGEA Srl)	75	1.35 ms	1.6	6	3	No significant changes between sacrifice timepoints (week 3 and 6).	Acceleration of HA implant osseointegration on trabecular bone.	Microhardness values measured in trabecular bone at 200 and 500 µm from the implants were significantly higher.

Ref	Condition	Animal model			Stimuli characteristics						Outcomes induced by IC stimulation		
		Species	Breed	Modification	Type of stimulation<br>Stimulation device<br>Waveform	Frequency (Hz)	Periodicity	MF strength (mT)	Exposure time (h/day)	Assay duration (weeks)	Radiologic	Histological and Biochemical	Biomechanical
59	Implant	Rabbit – *Oryctolagus cuniculus*	New Zealand White	HA rod implants in femur	PEMF (Biostim, IGEA, Carpi, Italy);	75	1.35 ms duty cycle	1.6	6	3	*NA*	PEMF stimulation improved osseointegration of HA implants compared with the nontreated group by increasing bone growth, mineral apposition rate and contact with the rods.	PEMF group had the highest values of maximum push-out force (Fmax) and ultimate shear strength (σu) after 3 weeks of treatment.
60	Implant	Rabbit – *Oryctolagus cuniculus*	New Zealand White	Pure dental titanium implant fixtures in tibiae metaphysis	PEMF	20 MHz	Pulse bursts width of 85 µs	*ND*	0.5	21 or 42 days	*NA*	Differences between test and control groups were not observed in histological morphology evaluation.	The improvement in removal torque tests was not statistically significant at the end of the experiment (42 days).
61	Implant	Rabbit – *Oryctolagus cuniculus*	New Zealand White	Porous titanium (pTi) implants in hindlimb bone defect; type 1 diabetes mellitus induced through alloxan injection	Pulsed burst PEMF	15 (pulse); 5000 (burst)	5 ms pulse bursts width and 60 ms wait; 0.2 ms pulse width; 0.02 ms pulse wait; 0.3 µs pulse rise; 2.0 µs pulse fall	2.0	2	8	Improved bone microarchitecture and pTi osseointegration in diabetic rabbits.	T1DM-associated reduction of bone formation attenuated by PEMF, but no impacts on bone resorption. Activation of osteoblastogenesis related to Wnt/β-catenin signalling in diabetic rabbits' skeleton, but no change in osteoclastogenesis-associated RANKL/RANK signalling gene expression.	Increased bone elastic modulus and bone hardness in diabetic rabbits.
62	Implant; Bone Maintenance (glucocorticoid degeneration)	Rabbit – *Oryctolagus cuniculus*	New Zealand White	Glucocorticoid treatment (dexamethasone); Porous titanium (pTi) implants in hindlimb bone defect;	Pulsed burst PEMF	15 (pulse); 5000 (burst)	5 ms pulse bursts width and 60 ms wait; 0.2 ms pulse width; 0.02 ms pulse wait; 0.3 µs pulse rise; 2.0 µs pulse fall	2.0	2	4	Ameliorated glucocorticoid-mediated deterioration of cancellous and cortical bone architecture and pTi osseointegration.	Reversal of the glucocorticoid's adverse effects on bone formation, attenuation of osteocyte apoptosis, promotion of osteoblast-related positive regulators, and inhibition of osteoblast negative regulators.<br>No effects on circulating bone-resorbing cytokines.	Increase in bone elastic modulus and bone hardness in glucocorticoid treated rabbits.

Ref	Condition	Animal model			Stimuli characteristics						Outcomes induced by IC stimulation		
		Species	Breed	Modification	Type of stimulation<br><br>Stimulation device<br><br>Waveform	Frequency (Hz)	Periodicity	MF strength (mT)	Exposure time (h/day)	Assay duration (weeks)	Radiologic	Histological and Biochemical	Biomechanical
63	Soft Tissue Healing	Rabbit – *Oryctolagus cuniculus*	New Zealand White	Patellectomy	CMF	76.6	*ND*	0.04 (AC); 0.02 (0.06 amphi-magnetic field) (DC)	0.5	8 or 16	*NA*	Increase in newly formed bone and regenerated fibrocartilage zone.	Energy to failure not significantly different between the 2 groups at week 8.<br>At week 16, load to failure, ultimate strength, and energy to failure higher than those in control animals.
64	Bone fracture	Dog – *Canis lupus familiaris*	Beagle	Fibular osteotomy	PEMF	1 vs 65	1 ms pulse or 150 μs pulse	*ND*	24	4	Increase in the tempo of the repair response.	65 Hz and 20 mV/cm (above the natural occurring biologic events of 1Hz and 2 mV/cm) lead to predominantly parallel orientation of the callus fibre bundles as well as an advance in ossification patterns, with less cartilage or chondroid material	The induced voltage field appears to increase bone strength 28 days after "fracture."
65	Late bone healing phase	Dog – *Canis lupus familiaris*	Mixed breed	Tibial osteotomy	PEMF (EBI, L.P. Parsippany); Asymmetrical pulses	1.5 (pulse)	30 ms pulse bursts	0 to 0.2	1	8	Enhancing effects on callus formation and maturation in the late-phase of bone healing	Significant increase in new bone formation and mineral apposition rate	Faster recovery of load-bearing and higher mechanical strength
66	Spine fusion	Dog – *Canis lupus familiaris*	Beagle	L5-L6 destabilization followed by posterolateral spinal fusion	PEMF (American Medical Eletronics, Inc)	1.5 (pulse); 3846 (burst)	670 ms pulse bursts of 99 pulses with 65 μs (+) and 195 μs (-)	*ND*	6	24	Increased BMD of the anterior vertebral body after posterolateral fusion surgery	*NA*	Increase in flexion and bending stiffness, although not statistically significant.

Ref	Condition	Animal model			Stimuli characteristics						Outcomes induced by IC stimulation		
		Species	Breed	Modification	Type of stimulation<br><br>Stimulation device<br><br>Waveform	Frequency (Hz)	Periodicity	MF strength (mT)	Exposure time (h/day)	Assay duration (weeks)	Radiologic	Histological and Biochemical	Biomechanical
67	Osteoporosis	Dog – *Canis lupus familiaris*	Beagle	OVX	PEMF (EBI, L.P. Parsippany); Assymetrical pulses	1.5 (pulse)	30 ms pulse bursts	*ND*	1	12	*NA*	PEMF stimulation reduced bone loss significantly from 23.1% to 9.5%. However, there was no significant difference in new bone formation on the periosteal surface of prepared fibulae nor change in number of osteons formed within the bones' cortex, their radial closure rate, or their degree of closure between stimulated and non-stimulated groups. Therefore, PEMF reduced bone resorption, with no effect on remodelling within the cortex.	*NA*
68	Osteoarthritis	Dog – *Canis lupus familiaris*	ND	Three cartilage defects on the stifle joint (equivalent to knee), followed by tissue-engineered (TE) repair using osteochondral grafts	PEMF (Custom-made; similar to IGEA Clinical Biophysics)	75	1.3 ms pulse duration; 0.10 duty cycle	1.5	6	13	*NA*	Overall, PEMF improved *in vivo* cartilage growth and repair. PEMF-stimulated TE repairs were significantly less likely than non-stimulated to have cartilage pathology (-70%). TE repair integration was improved by PEMF, although not significantly probably due to changes in PEMF orientation from animals' movement. Both groups had similar recovery of functional limitations.	*NA*
69	Spine fusion	Dog – *Canis lupus familiaris*	Mixed breed	L1-2 and L4-5 facet fusion	PEMF	1.5 (pulse); 3846 (burst)	30 ms pulse bursts: 260 μs burst	0.1	0.5 or 1	12	No significant differences in callus formation and bony union.	No significant differences in bony union and fibrocartilage/bone ratio.	*NA*
70	Implant	Dog – *Canis lupus familiaris*	Mixed breed	Dental implants placed in the mandible	Constant electromagnetic field (CEF)	1.5×10^6	25 μs pulse width	0.8	0.33	2	*NA*	CEF stimulation did not improve the bone-healing process around dental implants.	*NA*

Ref	Condition	Animal model			Stimuli characteristics						Outcomes induced by IC stimulation		
		Species	Breed	Modification	Type of stimulation<br><br>Stimulation device<br><br>Waveform	Frequency (Hz)	Periodicity	MF strength (mT)	Exposure time (h/day)	Assay duration (weeks)	Radiologic	Histological and Biochemical	Biomechanical
71	Non-union (Abstract only)	Dog – *Canis lupus familiaris*	Beagle	Osteotomy	PEMF	*ND*	*ND*	*ND*	*ND*	4	Not superior than control	*NA*	Not stronger than control
72	Osteotomy (Abstract only)	Dog – *Canis lupus familiaris*	Mixed breed	*ND*	PEMF	*ND*	*ND*	*ND*	*ND*	26	No significant effect on time to union with either two months or six months.	*NA*	No significant effect on the biomechanical strength.
73	Osteotomy	Sheep – *Ovis aries*	Black face	Tibial osteotomy	PEMF (Bio-Osteogen); trapezoidal waveform	*ND*	*ND*	1.06 – 2.56	24	2 or 6	All the bones healed and there were no statistically significant differences between the treated animals and the controls except in the uptake of bone-seeking mineral at 2 weeks.	Histological examination revealed no qualitative nor quantitative (cortical bone, mineralized callus and fibrocallus area) differences between control and stimulated group.	*NA*

Supplementary Table 2 – Biological outcomes quantified during IC stimulation studies in animal models of musculoskeletal disorders. Data was collected from 66 studies and includes the evaluated musculoskeletal disorder/condition, the animal model characteristics (species, breed, and modification or procedures the animal was submitted), and quantitative outcomes (radiologic, histological or biochemical, and biomechanical) measured in each study. Each measured outcome is presented as control vs stimulation (when available), at each recorded timepoint (indicated in bold), followed by the gain/loss percentage. AC – alternating voltage; CV – constant voltage; ES – electrical stimulation; BMD – bone mineral density; BV/TV – bone volume per total volume ration; Tb.Th – Trabecular thickness; Tb.N – Trabecular number; Tb.S – Trabecular separation; ALP – Alkaline phosphatase; OC – osteocalcin; Col I – collagen type I; FN – fibronectin; HA – hydroxyapatite; HU – hindlimb unloaded; OVX – Ovariectomized; PEMF – Pulsed Electromagnetic Field; CMF – Combined Magnetic Field; ND – not defined; NA – not applicable (not assessed by authors).

Ref.	Condition	Animal model			Outcomes Quantification: control vs stimulation (% of increase or decrease) at each recorded timepoint		
		Specie	Breed	Modification	Radiologic	Histological and Biochemical	Biomechanical
1	*NA*	Rat – *Rattus norvegicus*	Wistar	Healthy rats	*NA*	**In anterior and posterior cortical bone, and trabecular bone:**<br>Bone thickness: 260 vs 398 (+53.1%); 252 vs 380 (+50.8%); 112 vs 168 (+50.0%)	*NA*
2	*NA*	Rat – *Rattus norvegicus*	Wistar	Healthy rats	BMD (g/cm^2): 0.115 vs 0.126 (+9.6%) [femur]; 0.118 vs 0.130 (+10.2%) [L5 vertebral body]<br>BV/TV: 42.5% vs 72.0% (+1.69-fold)<br>Tb.Th (µm): 8.2 vs 11.4 (+39.0%)<br>Tb.N (1/mm): 0.052 vs 0.063 (+21.2%)<br>Tb.S (µm): 13.1 vs 7.8 (-40.5%)	PINP (ng/mL): 240 vs 276 (+15.0%)<br>CTX-I (ng/mL): 408 vs 330 (-19.1%)<br>sAC (relative optical density): 0.80 vs 1.19 (+48.8%)<br>PKA and p-PKA (relative optical density): 1.01 vs 1.02 (+0.9%) and 0.86 vs 1.19 (+38.4%)<br>CREB and p-CREB (relative optical density): 1.12 vs 1.10 (-1.7%) and 0.77 vs 1.09 (+41.5%)<br>**At 0, 15, 30-, 60-, 90- and 120-min PEMF:**<br>Serum cAMP (pg/mL): 100.5; 109; 116.5; 108.5; 102.5; 105.5	Maximum load (N): 284 vs 310 (+9.2%) [femur]; 105 vs 120 (+14.3%) [L5 vertebral body]
3	Deficient endochondral ossification	Rat – *Rattus norvegicus*	Sprague Dawley	Confinement of immature rats in a box for movement restriction (deficient endochondral ossification model); decalcified bone matrix (DBM) powder implanted subcutaneously along the thoracic musculature	*NA*	*NA*	*NA*
4	Bone defect	Rat – *Rattus norvegicus*	Wistar	Pre-maxilla bone defect grafted with demineralized bone matrix (DBM)	*NA*	Serum ALP activity: +2-fold<br>^{45}Ca incorporation: +3-fold	*NA*

Ref.	Condition	Animal model			Outcomes Quantification: control vs stimulation (% of increase or decrease) at each recorded timepoint		
		Specie	Breed	Modification	Radiologic	Histological and Biochemical	Biomechanical
5	Bone defect	Rat – *Rattus norvegicus*	Wistar	Femur incision with drill	(assessed at D21 upon therapy onset)<br>**For 1, 5 and 10 mT:**<br>BMD (g/cm^2): 0.189 vs 0.207 (+9.5%), 0.189 vs 0.226 (+19.6%), 0.189 vs 0.221 (+16.9%)	(assessed at D21 upon therapy onset)<br>**For 1, 5 and 10 mT:**<br>Serum Ca^{2+} (mmol/L): 2.14 vs 2.12 (-0.9%), 2.14 vs 2.46 (+15.0%), 2.14 vs 2.2 (+2.8%)<br>Serum P^{3-} (mmol/L): 2.29 vs 2.19 (-4.4%), 2.29 vs 2.28 (-0.4%), 2.29 vs 2.20 (-3.9%)<br>Serum ALP (u/L): 162.88 vs 177.15 (+8.8%), 162.88 vs 199.63 (+22.6%), 162.88 vs 182.59 (+12.1%)	(assessed at D21 upon therapy onset)<br>**For 1, 5 and 10 mT:**<br>Maximum load (N): 187.81 vs 181.00 (-3.6%), 187.81 vs 199.25 (+6.1%), 187.81 vs 197.21 (+5.0%)<br>Fracture Load (N): 155.14 vs 160.88 (+3.7%), 155.14 vs 185.2 (+19.4%), 155.14 vs 183.05 (+18.0%)<br>Elastic Load (N): 119.14 vs 110.09 (-7.6%), 119.14 vs 128.77 (+8.1%), 119.14 vs 130.03 (+9.1%)<br>Elastic radius (mm): 0.251 vs 0.269 (+7.2%), 0.251 vs 0.327 (+30.3%), 0.251 vs 0.312 (+24.3%)<br>Bending energy (N.mm): 14.51 vs 14.10 (-2.8%), 14.51 vs 16.82 (+15.9%), 14.51 vs 17.20 (+18.5%)
6	Osteotomy	Rat – *Rattus norvegicus*	Sprague Dawley	Tibial osteotomies, stabilized with external fixators, distracted 0.375 mm twice daily for 6 days	BV/TV: 0.315 vs 0.425 (+34.9%)<br>BMD (g/cm^2): 336 vs 403 (+19.9%)	BV/TV (from histological sections): 15.0 vs 21.3 (+42.0%)<br>Mineralized cartilage volume/TV: 21.3 vs 28.1 (+31.9%)<br>Unmineralized tissue volume/TV: -17.9 vs -4.8 (-73.2%)<br>Col I area: 53% vs 73% (+1.38-fold)<br>OCN area: 21% vs 36% (+1.71-fold)	Ultimate load: 0.155 vs 0.420 (+171.0%)<br>Energy to failure: 0.14 vs 0.47 (+235.7%)<br>Elastic modulus: 0.215 vs 0.420 (+95.3%)
7	Bone Fracture	Rat – *Rattus norvegicus*	Sprague Dawley	Tibia fracture	Total summed new bone area (mm^3): 1.77 vs 2.61 (+47.5%);<br>Total summed tibia bone area (mm^3): 7.78 vs 7.98 (+2.6%);<br>Total summed trabecular area (mm^3): 0.78 vs 0.59 (-24.4%),<br>Total summed cartilage area (mm^3): 3.08 vs 1.6 (-48.1%)	Serum OC (ng/mL): 12.45 vs 17.03 (+36.8%)	*NA*
8	Bone Fracture	Rat – *Rattus norvegicus*	Wistar	Femur fracture	*NA*	**At days 21 and 30:**<br>Histopathological score (1-10): 6.3 vs 7.6 (+20.6%), 6.5 vs 7.8 (+20.0%)	*NA*
9	Bone defect	Rat – *Rattus norvegicus*	Sprague Dawley	Calvaria defect model to test recombinant human bone morphogenetic protein-2 (rhBMP-2) in bone regeneration	BV (mm^3): 2.676 vs 12.048 (+350.2%)<br>BMD (mg/cm^3): 0.962 vs 1.023 (+6.3%)<br>Tb.Th (mm): 0.154 vs 0.39 (+153.2%)<br>Tb.N (1/mm): 0.104 vs 0.178 (+71.2%)<br>Tb.S (µm): 2.698 vs 2.158 (-20.0%)	Bone area: 0.892% vs 10.053% (+11.3-fold)<br>Dead space: 99.108% vs 89.947% (+0.907-fold)<br>vWF+ cells (n): 5.2 vs 7.6 (+46.2%)<br>OPN+ cells (n): 0.8 vs 14.8 (+1750.0%)<br>TRAP+ cells (n): 0 vs 3	*NA*

Ref.	Condition	Animal model			Outcomes Quantification: control vs stimulation (% of increase or decrease) at each recorded timepoint		
		Specie	Breed	Modification	Radiologic	Histological and Biochemical	Biomechanical
10	Bone Fracture	Rat – *Rattus norvegicus*	Wistar	Femur fracture stabilized with titanium nails	BV/TV: 29.87% vs. 34.22% (+1.14-fold); Tb.Th (mm): 0.49 vs 0.58 (+18.4%); Tb.N (n/mm^2): 0.72 vs. 0.72 (+0.0%); Tb.S (mm): 1.03 vs. 0.86 (-16.5%)	Serum ALP (U/L): 638.6 vs. 777.1 (+21.7%); Serum OC (pg/ml): 32.27 vs. 39.21 (+21.5%)	Mechanical strength (N): 21.83 vs. 35.1 (+60.8%); Elastic deformation (mm): 1.03 vs. 0.7 (-32.0%)
11	Non-union	Rat – *Rattus norvegicus*	Sprague Dawley	Fibular osteotomy Treatment 28 days after surgery	Change in distal fibula bone volume: -38.5% vs -12.0% (+0.31-fold)	*NA*	*NA*
12	Bone fracture	Rat – *Rattus norvegicus*	Sprague Dawley	Fibular osteotomy	**PEMF 1.5 Hz, 0.02 mT (OsteoStim®):** Callus formation rate: 2 vs 1.75 (-12.5%) **PEMF 15 Hz, 2.0 mT (PhysioStim®):** Callus formation rate: 2.5 vs 5 (+100.0%)	*NA*	**PEMF 1.5 Hz, 0.02 mT (OsteoStim®):** Bending test (apparent modulus/stiffness) (Mpa): 3.75 vs 4 (+6.7%) **PEMF 15 Hz, 2.0 mT (PhysioStim®):** Bending test (apparent modulus/stiffness) (Mpa): 3.5 vs 7 (+100.0%)
13	Bone Fracture	Rat – *Rattus norvegicus*	Sprague Dawley	Ulnar osteotomy	**Callus quality mean score (week 2-8; 1 measurement/week):** 0.13 vs 0.06 (-53.8%), 0.83 vs 0.42 (-49.4%), 1.44 vs 0.39 (-72.9%), 1.50 vs 1.17 (-22.0%), 1.69 vs 1.18 (-30.2%), 1.69 vs 1.12 (-33.7%), 1.38 vs 1.24 (-10.1%)	**Mean Histological Score:** Bone: 24.03 vs 24.1 (+0.3%), Cartilage: 35.34 vs 17.95 (-49.2%), Fibrous Tissue: 35.32 vs 57.95 (+64.1%)	*NA*
14	Spine fusion	Rat – *Rattus norvegicus*	Sprague Dawley	Posterior vertebral arthrodesis in last 3 lumbar vertebrae	*NA*	*NA*	*NA*
15	Soft Tissue Healing	Rat – *Rattus norvegicus*	Sprague Dawley	Rotator cuff tearing	**At weeks 4, 8, 16:** BMD (mg.HA/cm^3): 455 vs 440 (-3.3%), 545 vs 570 (+4.6%), 580 vs 635 (+9.5%); BV/TV: 0.44 vs 0.43 (-2.3%), 0.515 vs 0.54 (+4.9%), 0.555 vs 0.6 (+8.1%); Tb.Th (mm): 0.205 vs 0.177 (-13.7%), 0.245 vs 0.243 (-0.8%), 0.270 vs 0.289 (+7.0%); Tb.N (1/mm): 2.93 vs 3.27 (+11.6%), 2.67 vs 2.89 (+8.2%), 2.74 vs 2.83 (+3.3%); Tb.S (mm): 0.385 vs 0.335 (-13.0%), 0.465 vs 0.385 (-17.2%), 0.425 vs 0.400 (-5.9%)	*NA*	**At weeks 4, 8, 16:** Cross-sectional area (mm^2): 3.8 vs 2.7 (-28.9%), 4.4 vs 4.1 (-6.8%), 3.6 vs 3.2 (-11.1%); Modulus (Mpa): 11 vs 22 (+100%), 15 vs 24 (+60.0%), 46 vs 29 (-37.0%); Stiffness (N/mm): 6.3 vs 7.9 (+25.4%), 12.7 vs 12.6 (-0.8%), 13.6 vs 12.8 (-5.9%); Max stress (Mpa): 2.3 vs 2.9 (+26.1%), 4.3 vs 3.8 (-11.6%), 5.2 vs 4.8 (-7.7%); Max load (N): 9.6 vs 7.4 (-22.9%), 17.2 vs 15.0 (-12.8%), 16.6 vs 16.4 (-1.2%)

Ref.	Condition	Animal model			Outcomes Quantification: control vs stimulation (% of increase or decrease) at each recorded timepoint		
		Specie	Breed	Modification	Radiologic	Histological and Biochemical	Biomechanical
16	Soft Tissue Healing	Rat – *Rattus norvegicus*	Sprague Dawley	Rotator cuff tearing	*NA*	**Collagen fiber alignment** (significant results) (circular standard deviation; in degrees):<br>**At tendon insertion:**<br>HF-PEMF (3h/day, 16 weeks):<br>20.0 vs 13.2 (-34.0%)<br><br>**At tendon midsubstance:**<br>PEMF (1h/day, 16 weeks):<br>18.0 vs 15.2 (-15.6%)<br>HF-PEMF (1h/day, 16 weeks):<br>18.0 vs 6.9 (-61.7%)<br>HF-PEMF (3h/day, 8 weeks):<br>14.0 vs 7.0 (-50.0%)	**Stiffness** (N/mm):<br>Week 4:<br>5.5 vs 11.2 (+103.0%) [PEMF 1 h/day];<br>5.5 vs 8.2 (+49.1%) [PEMF 3 h/day];<br>5.5 vs 12.7 (+130.9%) [HF-PEMF 3 h/day];<br>5.5 vs 9.3 (+69.1%) [HF-PEMF 6 h/day]<br><br>Week 8:<br>9.7 vs 14.7 (+51.5%) [PEMF 1h/day];<br>9.7 vs 15.3 (+57.7%) [PEMF 6 h/day];<br>9.7 vs 17.8 (+83.5%) [HF-PEMF 1 h/day];<br>9.7 vs 16.7 (+72.2%) [HF-PEMF 3 h/day];<br>9.7 vs 14.3 (+47.4%) [HF-PEMF 6 h/day]<br><br>Week 16:<br>12.3 vs 20.3 (+65.0%) [PEMF 1 h/day];<br>12.3 vs 18.7 (+52.0%) [HF-PEMF 1 h/day];<br><br>**Modulus** (Mpa):<br>Week 4:<br>10 vs 22 (+120.0%) [PEMF 1 h/day];<br>10 vs 19 (+90.0%) [PEMF 3 h/day];<br>10 vs 32 (+220.0%) [PEMF 6 h/day]<br><br>Week 8:<br>15 vs 24 (+60.0%) [PEMF 3 h/day];<br>15 vs 22 (46.7%) [PEMF 6 h/day];<br>15 vs 22 (+46.7%) [HF-PEMF 1 h/day];<br>15 vs 45 (+200.0%) [HF-PEMF 6 h/day]
17	Soft Tissue Healing	Rat – *Rattus norvegicus*	Sprague Dawley	Transection and modified Kessler repair of the Achilles' tendon<br>+ 1 week limb immobilization.	*NA*	**Complete tear model with immobilization at 1 week:**<br>Cellularity: 1.8 vs 2.4 (+33.3%) [1 h/day];<br>1.8 vs 1.68 (-6.7%) [3 h/day];<br>Cell Shape: 1.5 vs 2.1 (+40.0%) [1 h/day];<br>1.5 vs 2.1 (+40.0%) [3 h/day];<br><br>**Partial tear model with immobilization at 3 weeks:**<br>Cellularity: 2.7 vs 1.8 (-33.3%) [1h/day];<br>2.7 vs 2.1 (-22.2%)	**Complete tear model with immobilization at 1 week:**<br>Cross-sectional area (mm^2):<br>7.2 vs 9.2 (+27.8%) [1 h/day];<br>7.2 vs 9.7 (+34.7%) [3 h/day];<br>Stiffness (N/mm): 2.3 vs 0.3 (-86.9%) [1 h/day];<br>Modulus (Mpa): 9.0 vs 1.0 (-88.9%) [1 h/day];<br>9.0 vs 4.0 (-55.5%) [3 h/day];<br>Load to Failure (N): 8.7 vs 0.7 (-91.9%) [1h/day].<br><br>**Complete tear model with immobilization at 3 weeks:**<br>Stiffness (N/mm): 25.0 vs 15.3 (-38.8%) [1h/day];<br>25.0 vs 17.7 (-29.2%) [3h/day];
18	Osteoporosis	Rat – *Rattus norvegicus*	Sprague Dawley	OVX	(Values for L4/L5 vertebral bodies)<br>BMD: +19.5%<br>BV/TV: +16.4%;<br>Tb.Th: +9.1%;<br>Tb.N: +16.4%;<br>Tb.S: -9.5%	Serum ALP (U/L): 91 vs 94 (+3.3%);<br>Serum TRACP5b (U/L): 17.6 vs 13.0 (-26.1%);<br><br>(Values for L3 vertebral bodies)<br>OPG: 0.31 vs 1.18 (+3.8-fold);<br>RANKL: 2.22 vs 1.31 (-41.0%)	(Values for L5 vertebral bodies)<br>Load-bearing (N): +12.7%;<br>Energy to failure (mJ): +29.2%

Ref.	Condition	Animal model			Outcomes Quantification: control vs stimulation (% of increase or decrease) at each recorded timepoint		
		Specie	Breed	Modification	Radiologic	Histological and Biochemical	Biomechanical
19	Osteoporosis	Rat – *Rattus norvegicus*	Sprague Dawley	OVX	**At early and late phase:** BMD: +32.78%, +29.51% (L5 vertebral body); +11.4%, +4.55% (Femur); BV/TV [L4 vertebral body]: 32.5% vs 36.5% (+1.12-fold), 31% vs 38% (+1.23-fold); Tb.Th (μm) [L4 vertebral body]: 79 vs 88 (+11.4%), 76 vs 87 (+14.5%); Tb.N (1/mm) [L4 vertebral body]: 4.05 vs 4.55 (+12.3%), 3.95 vs 4.75 (+20.3%); Tb.S (μm) [L4 vertebral body]: 168 vs 140 (-16.7%), 173 vs 144 (-16.8%)	**At early and late phase:** (values from femur tissues) RANK/β-actin ratio (relative mRNA): 2.31 vs 2.19 (-5.2%), 3.05 vs 2.93 (-3.9%); RANK/β-actin ratio (relative protein quantification): 0.336 vs 0.220 (-34.5%), 0.399 vs 0.330 (-17.3%)	**At early and late phase:** (values from L5 vertebral body) Load-bearing (N): 353 vs 386 (+9.3%), 313 vs 361 (+15.3%); Energy to failure (mJ): 40.5 vs 53 (+30.9%), 42 vs 49 (+16.7%); Stiffness (N/mm): 572 vs 616 (+7.7%), 562 vs 628 (+11.7%)
20	Osteoporosis	Rat – *Rattus norvegicus*	Wistar	OVX	*NA*	*NA*	*NA*
21	Osteoporosis	Rat – *Rattus norvegicus*	Sprague Dawley	OVX; Fibular fracture	BV/TV not different (data not shown); Bone repair response: 6 vs 6.7 (+11.7%); Fracture bridging: 2.3 vs 2.7 (+17.4%)	*NA*	Elastic modulus (MPa): 368 vs 621 (+68.8%)
22	Osteoporosis	Rat – *Rattus norvegicus*	Wistar	Periodontitis (P); Periodontitis + PEMF (P+PEMF); Ovariectomy + Periodontitis (P+OVX); Ovariectomy + Periodontitis + PEMF (P+OVX+PEMF). PEMF therapy began 7 days after periodontitis induction.	**P vs P+PEMF and P+OVX vs P+OVX+PEMF:** BV/TV (%): 47.0 vs 56.5 (+20.2%); 23.0 vs 31.5 (+37.0%) BMD (mg/cm^3): 0.53 vs 0.72 (+35.2%); 0.34 vs 0.41 (+20.4%) Tb.N (1/mm): 1.15 vs 1.26 (+9.6%); 0.90 vs 1.02 (+13.3%) Tb.S (μm): 0.38 vs 0.35 (-7.1%); 0.43 vs 0.39 (-8.9%)	**P vs P+PEMF and P+OVX vs P+OVX+PEMF:** Bone Loss (BL, mm^2): 1.64 vs 1.34 (-18.3%); 1.73 vs 1.39 (-19.7%) Connective Tissue Attachment Loss (CTAL, mm): 1.19 vs 1.29 (+8.4%); 1.45 vs 1.20 (-17.2%) Alveolar Bone Loss (ABL, mm): 1.59 vs 1.71 (+7.5%); 1.97 vs 1.63 (-17.3%)<br><br>IL-1β (pg/mL): 980 vs 60 (-93.9%); 340 vs 80 (-76.5%) IL-6 (pg/mL): 400 vs 40 (-90.0%); 896 vs 96 (-89.3%) IL-10 (pg/mL): 360 vs 60 (-83.3%); 980 vs 120 (-87.8%) VEGF (pg/mL): 205 vs 40 (-80.5%); 535 vs 80 (-85%) TNF-α (pg/mL): 116 vs 28 (-75.9%); 400 vs 44 (-89.0%)	*NA*
23	Osteoporosis	Rat – *Rattus norvegicus*	Sprague Dawley	OVX	BV/TV: 15.3% vs 28.0% (+1.83-fold) BMD (mg/cm^3): 196 vs 278 (+41.8%) Tb.Th (μm): 68 vs 80 (+17.6%) Tb.N (1/mm): 1.90 vs 2.94 (+54.7%) Tb.S (μm): 354 vs 288 (-18.6%) SMI (#): 2.90 vs 1.98 (-31.7%)	Wnt1 (mRNA): 1.22 vs 1.68 (+37.7%) LRP5 (mRNA): 1.29 vs 1.82 (+41.1%) β-catenin (mRNA): 1.48 vs 1.74 (+17.6%) RANKL (mRNA): 1.28 vs 1.81 (+41.4%) RANK (mRNA): 1.29 vs 1.18 (-8.5%)	Maximum Load (N): 151 vs 174 (+15.2%) Energy Absorption (N.mm): 78 vs 95 (+21.8%) Stiffness (N/mm): 158 vs 162 (+2.5%) Elastic Modulus (GPa): 5.8 vs 8.5 (+46.6%)

Ref.	Condition	Animal model			Outcomes Quantification: control vs stimulation (% of increase or decrease) at each recorded timepoint		
		Specie	Breed	Modification	Radiologic	Histological and Biochemical	Biomechanical
24	Osteoporosis	Mouse – *Mus musculus*	C57BL/6	OVX	BV/TV: 1.9% vs 3.4% (+1.5%)<br>BMD (g/cm^3): 1.34 vs 1.58 (+17.9%)<br>Tb.Th (µm): 34 vs 50 (+47.1%)<br>Tb.N (1/mm): 0.33 vs 0.69 (+109.1%)<br>Tb.S (mm): 0.66 vs 0.47 (-28.8%)	Serum ALP (ng/mL): 5 vs 5.6 (+12.0%)<br>Serum OC (ng/mL): 3.4 vs 3.8 (+11.8%)<br>Serum CTX-I (ng/mL): 121 vs 95 (-21.5%)<br>Serum TRACP5b (ng/mL): 10.4 vs 7.8 (-25.0%)<br>Osterix (mRNA): 0.57 vs 1.58 (+177.2%)	*NA*
25	Osteoporosis	Rat – *Rattus norvegicus*	Sprague Dawley	OVX	**At week 10 (stimulation until week 6) with 1.2 mT):**<br>BV/TV loss: -19% vs -12%<br>Trabecular BMC loss: -40% vs -25% (+0.63-fold)<br>Trabecular BMD loss: -19% vs -12% (+0.63-fold)	**At week 10 (stimulation until week 6) with 1.2 mT):**<br>Lacunar area (µm^2): 751 vs 787 (+4.8%)<br>Lacunar size (µm^2): 50.2 vs 51.4 (+2.4%)<br>Lacunae area: 5.0% vs 5.2% (+1.04-fold)<br>Canalicular Length (µm): 109 vs 130 (+19.3%)<br>Osteoclast number (cells/mm): 0.93 vs 0.26 (-72.0%)<br><br>**At week 10 (stimulation until week 6) with 4.1 mT):**<br>Lacunar area (µm^2): 751 vs 891 (+18.6%)<br>Lacunar size (µm^2): 50.2 vs 49.2 (-2.0%)<br>Lacunae area: 5.0% vs 5.9% (+1.18-fold)<br>Canalicular Length (µm): 109 vs 106 (-2.8%)<br>Osteoclast number (cells/mm): 0.93 vs 1.26 (+35.5%)	*NA*
26	Osteoporosis	Rat – *Rattus norvegicus*	Sprague Dawley	Hindlimb unloading (HU) model via tail suspension	**At week 12:**<br>Tb.Area: 8.6% vs 22.0% (+2.56-fold)<br>Tb.N (1/mm): 1.3 vs 2.1 (+61.5%)<br>Tb.Th (µm): 45.6 vs 48.4 (+6.1%)<br>Tb.S (µm): 228 vs 216 (-5.3%)	**At week 12:**<br>Serum OC: 2.7 vs 3.1 (+14.8%)	*NA*

Ref.	Condition	Animal model			Outcomes Quantification: control vs stimulation (% of increase or decrease) at each recorded timepoint		
		Specie	Breed	Modification	Radiologic	Histological and Biochemical	Biomechanical
27	Osteoporosis	Rat –<br>*Rattus norvegicus*	Sprague Dawley	Hindlimb unloading (HU) model via tail suspension	BV/TV: 10.2% vs 18.6% (+1.82-fold)<br>BMD (mg/cm^3): 186 vs 252 (+35.5%)<br>Tb.Th (µm): 47.5 vs 60.0 (+26.3%)<br>Tb.N (1/mm): 2.70 vs 1.76 (-34.8%)<br>Tb.S (µm): 440 vs 296 (-32.7%)<br>SMI (#): 2.62 vs 1.84 (-39.7%)<br>Conn.D (1/mm^3): 24.0 vs 42.4 (+76.7%)<br>BS/BV (1/mm): 39.0 vs 31.6 (-18.9%)<br>Ct.Ar (mm^2): 4.36 vs 4.96 (+13.8%)<br>Ct.Th (µm): 472 vs 516 (+9.3%)<br>Tt.Ar (mm^2): 10.1 vs 10.3 (+2.0%)<br>Ct.Ar/Tt.Ar: 43.5% vs 48.9% (+1.12-fold)	N.Ob/BS (1/µm): +53.3%<br>N.Oc/BS (1/µm): -12.4%<br>MAR (µm/d): +119.1%<br>BFR.BS (µm^3/µ^2/d): +118.8%<br>PINP (ng/mL): +59.2%<br>OC (ng/mL): +47.0%<br>CTX-I (ng/mL): -16.4%<br>TRACP5b (u/L): -15.2%<br><br>mRNA expressions:<br>Wnt1: 66% vs 122% (+1.85-fold)<br>LRP5: 64% vs 122% (+1.91-fold)<br>β-catenin: 60% vs 103% (+1.72-fold)<br>OPG: 50% vs 80% (+1.6-fold)<br>OC: 62% vs 102% (+1.65-fold)<br>RANKL: 99% vs 94% (+0.95-fold)<br>RANK: 113% vs 107% (+0.95-fold)<br>OPG/RANKL: 42% vs 80% (+1.90-fold)	Maximum Load (N): +22.7%<br>Yield Load (N): +40.0%<br>Ultimate Displacement (µm): +16.9%<br>Yield Displacement (µm): +18.6%<br>Stiffness (N/mm): +18.9%
28	Osteoporosis	Rat –<br>*Rattus norvegicus*	Sprague Dawley	Hindlimb unloading (HU) model via tail suspension	**At 1, 2, 4, 8 weeks:**<br>Proximal femur BMD (g/cm^2): 0.122 vs 0.123 (+0.8%), 0.109 vs 0.115 (+5.5%), 0.102 vs 0.120 (+17.6%), 0.098 vs 0.130 (+32.7%)	**At 1, 2, 4, 8 weeks:**<br>Serum IL-6 (ng/mL): 0.194 vs 0.189 (-2.6%), 0.216 vs 0.185 (-14.4%), 0.210 vs 0.175 (-16.7%), 0.207 vs 0.162 (-21.7%);<br>Serum TGF-β1 (ng/mL): 3.24 vs 2.95 (-9.0%), 1.96 vs 2.10 (+7.1%), 1.32 vs 1.66 (+25.8%), 1.26 vs 1.79 (+42.1%)	*NA*
28	Osteoporosis	Rat –<br>*Rattus norvegicus*	Wistar	Hindlimb unloading (HU) model via tail suspension	BV/TV: 11% vs 30% (+2.72-fold)<br>Tb.Th (µm): 138 vs 178 (+29.0%)<br>Tb.N (1/mm): 0.58 vs 1.08 (+86.2%)<br>Tb.S (µm): 738 vs 495 (-32.9%)<br>Ct.Th (µm): 435 vs 540 (+24.1%)<br>Ct.Ar (mm^2): 4.1 vs 5.3 (+29.3%)<br><br>**In femur and vertebral body:**<br>BMD (g/cm^2):<br>[healthy vs HU]<br>0.145 vs 0.107 (-26.2%),<br>0.125 vs 0.114 (-8.8%);<br>[healthy vs stimulated]<br>0.145 vs 0.126 (-13.1%),<br>0.125 vs 0.121 (-3.2%);<br>[HU vs stimulated]<br>0.107 vs 0.126 (+17.8%),<br>0.114 vs 0.121 (+6.1%)	Serum OC (ng/mL): 320 vs 760 (+137.5%)<br>Serum PINP (ng/mL): 300 vs 417 (+39.0%)<br>Serum PTH (ng/mL): 11.7 vs 18.3 (+56.4%)<br>Serum CTX-I (ng/mL): 480 vs 390 (-18.8%)<br>Serum TRACP5b (ng/mL): 3.4 vs 2.2 (-35.3%)<br>cAMP (ng/mL): 67 vs 138 (+106.0%)<br>Osteoblasts (endosteum)(n/mm^2): 29 vs 45 (+55.2%)<br>Osteoblasts (trabeculae)(n/mm^2): 60 vs 97 (+61.7%)<br>Adipocytes (n/mm^2): 202 vs 98 (-51.5%)	**In femur and vertebral body:**<br>Maximum Load (N): 108 vs 125 (+15.7%), 195 vs 255 (+30.8%)

Ref.	Condition	Animal model			Outcomes Quantification: control vs stimulation (% of increase or decrease) at each recorded timepoint		
		Specie	Breed	Modification	Radiologic	Histological and Biochemical	Biomechanical
30	Osteoporosis	Rat –<br>*Rattus norvegicus*	Sprague Dawley	Hindlimb unloading (HU) model via tail suspension	BV/TV: 10.2% vs 12% (+1.17-fold)<br>Tb.Th (µm):47.5 vs 49.0 (+3.2%)<br>Tb.N (1/mm):1.78 vs 1.90 (+6.7%)<br>Tb.S (µm): 444 vs 456 (+2.7%)<br>Ct.Th (µm): 472 vs 482 (+2.1%)<br>Ct.Ar (mm^2): 4.35 vs 4.18 (-3.9%)<br>BMD (mg/cm^3): 216 vs 184 (-14.8%)<br>SMI: 2.60 vs 2.38 (-8.5%)	Serum OC (ng/mL): 7.8 vs 8.2 (+5.1%);<br>P1NP (ng/mL): 21.8 vs 20.0 (-8.2%);<br>TRACP5b (U/L): 7.6 vs 7.0 (-7.9%);<br>CTX-1 (ng/mL): 37.0 vs 32.0 (-13.5%)	Maximum Load (N): 145 vs 134 (-7.6%);<br>Stiffness (N/mm): 300 vs 292 (-2.7%)<br>Energy Absorption (N.mm): 52 vs 43 (-17.3%)<br>Elastic Modulus (GPa): 8.4 vs 8.0 (-4.8%)
31	Osteoporosis	Rat –<br>*Rattus norvegicus*	Sprague Dawley	5 weeks heparin treatment	*NA*	New bone area: +4-fold;<br>Serum Ca2+ (mg/dL): 8.9 vs 9.1 (+2.2%);<br>Serum CTX (ng/mL):<br>248.9 vs 197.27 (-20.7%);<br>Serum PTH (pg/mL):<br>164.45 vs 172.8 (+5.1%)	*NA*
32	Osteoporosis	Rat –<br>*Rattus norvegicus*	Sprague Dawley	Glucocorticoid treatment (dexamethasone)	BMD (g/cm^2): +2.8%<br>BMC (g): +12.2%<br>Tb.N (1/mm): 5.25 vs 7.84 (+49.3%)<br>Tb.Ar: 26.95% vs 48.16% (+1.78-fold)<br>Tb.Wi (µm): 52.70 vs 61.42 (+16.5%)<br>Tb.S (µm): 139.8 vs 66.55 (-52.4%)	Serum ALP (u/L): 72 vs 114 (+58.3%)<br>Serum TRACP5b (u/L): 1.98 vs 1.77 (-10.6%)<br><br>**Quantification of mRNA and protein:**<br>Wnt10b: 0.55 vs 2.60 (+372.7%),<br>0.94 vs 1.38 (+46.8%)<br>LRP5: 0.82 vs 1.54 (+87.8%),<br>0.88 vs 1.20 (+36.4%)<br>β-catenin: 0.95 vs 1.34 (+41.1%),<br>0.92 vs 1.16 (+26.1%)<br>Runx2: 0.87 vs 1.06 (+21.8%),<br>0.92 vs 1.24 (+34.8%)<br>PPAR-γ: 1.22 vs 0.5 (-59.0%),<br>1.20 vs 0.84 (-30.0%)<br>C/EBPα: 1.18 vs 0.62 (-47.5%),<br>1.20 vs 0.52 (-56.7%)<br>FABP4: 1.54 vs 0.44 (-71.4%),<br>1.30 vs 0.34 (-73.8%)<br>Axin2: 1.34 vs 0.86 (-35.8%),<br>1.18 vs 0.70 (-40.7%)<br>Dkk-1: 1.06 vs 0.62 (-41.5%),<br>1.12 vs 0.90 (-19.6%)<br>SOST: 1.10 vs 0.92 (-16.4%),<br>1.06 vs 0.92 (-13.2%)<br>OPG: 0.78 vs 1.80 (+130.8%),<br>0.98 vs 1.16 (+18.4%)<br>RANKL: 1.36 vs 0.64 (-52.9%),<br>1.16 vs 0.48 (-58.6%)<br>OPG/RANKL: 0.52 vs 2.84 (+446.2%),<br>0.40 vs 1.54 (+285.0%)	*NA*

Ref.	Condition	Animal model			Outcomes Quantification: control vs stimulation (% of increase or decrease) at each recorded timepoint		
		Specie	Breed	Modification	Radiologic	Histological and Biochemical	Biomechanical
33	Osteoporosis	Rat – *Rattus norvegicus*	Sprague Dawley	Aged rats (24 months old) (senile osteoporosis model)	**In femur/tibia and vertebral body:**<br>BMD (g/cm^2): 0.27 vs 0.31 (+14.8%); 0.11 vs 0.14 (+27.3%)<br>BV/TV: 7.2% vs 12.2% (+1.69-fold); 10.8% vs 17.6% (+1.63-fold)<br>Tb.N (1/mm): 0.68 vs 1.23 (+80.9%); 1.24 vs 1.88 (+51.6%)<br>Tb.Th (µm): 0.102 vs 0.097 (-4.9%); 0.083 vs 0.087 (+4.8%)<br>Tb.S (µm): 0.97 vs 0.59 (-39.1); 0.59 vs 0.42 (-28.8%)	Serum bone-specific ALP (u/L): 6.4 vs 11.4 (+78.1%)<br>Serum TRACP5b (u/L): 0.82 vs 0.59 (-28.0%)<br><br>**Quantification of mRNA and protein:**<br>Wnt3a: 0.14 vs 0.44 (+214.3%), 0.39 vs 0.67 (+71.8%)<br>LRP5: 0.13 vs 0.42 (+223.0%), 0.52 vs 0.75 (+44.2%)<br>β-catenin: 0.29 vs 0.66 (+127.6%), 0.41 vs 0.72 (+75.6%)<br>Runx2: 3.70 vs 1.76 (-52.4%), 0.46 vs 0.79 (+71.7%)<br>PPAR-γ: 0.18 vs 0.56 (+211.1%), 1.62 vs 1.34 (-17.3%)	*NA*
34	Implant	Rat – *Rattus norvegicus*	Wistar	Tibial titanium implant	**With 1 h/day at days 3, 7, 21, 45:**<br>BV/TV: 2% vs 26.5% (+13.3-fold), 18% vs 47% (+2.61-fold), 25.5% vs 47% (+1.84-fold), 23% vs 28.5% (+1.24-fold); average (+4.7-fold)<br>Tb.Th (mm): 0.42 vs 0.125 (-70.2%), 0.47 vs 0.185 (-60.6%), 0.47 vs 0.245 (-47.9%), 0.46 vs 0.27 (-41.3%); average (-55%)<br>Tb.N (1/mm): 0.97 vs 0.96 (-1.0%), 0.52 vs 0.93 (+78.8%), 0.55 vs 0.90 (+63.6%), 0.52 vs 0.78 (+50.0%); average (+47.9%)<br>BMD (g/cm^2): 17.7 vs 17.1 (-3.4%), 17.9 vs 31.2 (+74.3%), 18.5 vs 29.7 (+60.5%), 17.1 vs 32.0 (+87.1%); average (+54.6%)<br><br>**With 3 h/day at days 3, 7, 21, 45:**<br>BV/TV: 2% vs 15% (+7.5-fold), 18% vs 11% (0.61-fold), 25.5% vs 17% (0.67-fold), 23% vs 10% (0.43-fold); average (2.30-fold)<br>Tb.Th (mm): 0.42 vs 0.34 (-19.0%), 0.47 vs 0.275 (-41.5%), 0.47 vs 0.315 (-33.0%), 0.46 vs 0.205 (-55.4%); average (-37.2%)<br>Tb.N (1/mm): 0.97 vs 0.92 (-5.2%), 0.52 vs 1.08 (+107.7%), 0.55 vs 0.73 (+32.7%), 0.52 vs 0.87 (+67.3%); average (+50.6%)<br>BMD (g/cm^2): 17.7 vs 17.0 (-4.0%), 17.9 vs 13.5 (-24.6%), 18.5 vs 17.3 (-6.5%), 17.1 vs 18.1 (+5.8%); average (-7.3%)	**With 1 h/day at days 3, 7, 21, 45:**<br>BIC: 12.5% vs 44% (+3.52-fold), 23% vs 62% (+2.70-fold), 44% vs 47.5% (+1.08-fold), 55% vs 76.5% (+1.39-fold); average (+2.17-fold)<br><br>Bone area fraction occupied (BAFO): 24% vs 53% (+2.20-fold), 46% vs 77% (+1.67-fold), 53% vs 51% (0.96-fold), 83% vs 84% (+1.01-fold); average (+1.46-fold)<br><br>**With 3 h/day at days 3, 7, 21, 45:**<br>BIC: 12.5% vs 39% (+3.12-fold), 23% vs 40% (+1.73-fold), 44% vs 52.5% (+1.19-fold), 55% vs 64% (+1.16-fold); average (+1.80-fold)<br><br>Bone area fraction occupied (BAFO): 24% vs 43% (+1.79-fold), 46% vs 63% (+1.37-fold), 53% vs 47% (0.88-fold), 83 vs 64 (0.77-fold); average (+1.20-fold)	**With 1 h/day at days 3, 7, 21, 45:**<br>Removal torque (N.cm): 1.7 vs 3.8 (+123.5%), 4.6 vs 5.5 (+19.6%), 5.0 vs 6.4 (+28.0%), 8.3 vs 9.7 (+16.9%); average (+47%)<br><br>**With 3 h/day at days 3, 7, 21, 45:**<br>Removal torque (N.cm): 1.7 vs 3.0 (+76.5%), 4.6 vs 2.9 (-37.0%), 5.0 vs 5.6 (+12.0%), 8.3 vs 9.8 (+18.1%); average (+17.4%)
35	Implant and fracture (Abstract only)	Rat – *Rattus norvegicus*	Wistar	Titanium implants in right tibial crest and osteotomy in left tibial crest	*NA*	*NA*	*NA*

Ref.	Condition	Animal model			Outcomes Quantification: control vs stimulation (% of increase or decrease) at each recorded timepoint		
		Specie	Breed	Modification	Radiologic	Histological and Biochemical	Biomechanical
36	Implant	Rat – *Rattus norvegicus*	Fisher Inbred	Titanium alloy pins implanted intramedullary in distal femurs followed by the injection of polyethylene particles to induce osteolysis	*NA*	BV/TV: 30.5% vs 46.5% (+1.52-fold)<br>BIC: 10.4% vs 25.0% (+2.40-fold)<br>Cortical width (Ct.Wi)(mm): 164 vs 111 (-32.3%)<br>Capsule thickness (mm): 53 vs 25 (-52.8%)<br>Osteoclast number: 166 vs 45 (-72.9%)	Hardness value (HV): 46.5 vs 55.5 (+19.4%)<br>Bone maturation index (BMI): 87.8 vs 88.5 (+0.8%)
37	Implant	Rat – *Rattus norvegicus*	Sprague Dawley	12-week-old; OVX; titanium implants in proximal metaphyses of tibia	**In 3 volumes of interest (VOI):**<br>BV/TV: 4.0% vs 7.0% (+1.75-fold), 22.4% vs 34.3% (+1.53-fold), 17% vs 26.5% (+1.56-fold);<br>Tb.Th (mm): 0.140 vs 0.142 (+1.4%), 0.130 vs 0.133 (+2.3%), 0.188 vs 0.180 (-4.3%);<br>Tb.N (1/mm): 2.027 vs 2.09 (+3.1%), 2.214 vs 2.170 (-2.0%), 2.051 vs 2.166 (+5.6%);<br>Tb.S (mm): 0.546 vs 0.718 (+31.5%), 0.572 vs 0.725 (+26.7%), 0.573 vs 0.634 (+10.6%)	*NA*	*NA*
38	Osteoarthritis	Rat – *Rattus norvegicus*	Sprague Dawley	Unilateral anterior crossbite group (UAC) to model temporomandibular joint osteoarthritis (TMJOA)	**At weeks 3 and 6:**<br>BV/TV: 45.6% vs 50.8% (+1.11-fold); 42.0% vs 58.8% (+1.40-fold)<br>BS/BV: 40.0% vs 33.0% (0.83-fold); 35.0% vs 25.0% (0.71-fold)<br>Tb.N (1/mm): 9.2 vs 8.6 (-6.5%); 7.7 vs 7.4 (-3.9%)<br>Tb.Th (mm): 0.049 vs 0.058 (+18.4%); 0.057 vs 0.078 (+36.8%)<br>Tb.S (mm): 0.057 vs 0.056 (-1.7%); 0.076 vs 0.054 (-29%)	**At weeks 3 and 6:**<br>TRACP5b+ cells (#): 8.4 vs 6.0 (-28.6%); 8.7 vs 3.0 (-65.5%)<br>OPG: 1.58 vs 2.74 (+73.4%); 0.48 vs 2.78 (+5.8-fold)<br>COL1A1: 1.04 vs 2.00 (+92.3%); 1.24 vs 4.40 (+3.5-fold)<br>ALP: 0.72 vs 1.45 (+101.4%); 0.73 vs 1.51 (+106.8%)<br>Runx2: 1.32 vs 1.20 (-9.1%); 0.64 vs 1.16 (+81.3%)<br>LRP5: 1.73 vs 4.47 (+2.6-fold); 2.67 vs 8.17 (+3.06-fold)<br>OCN: 1.32 vs 0.91 (-31.1%); 1.48 vs 0.99 (-33.1%)<br>RANKL: 1.32 vs 0.91 (-31.1%); 1.48 vs 0.98 (-33.8%)<br>BMP-2: 1.20 vs 1.60 (+33.3%); 0.64 vs 2.48 (+3.9-fold)<br>Wnt1: 0.75 vs 2.50 (+3.3-fold); 0.70 vs 5.80 (+8.3-fold)<br>β-catenin: 0.92 vs 1.14 (+23.9%); 1.12 vs 2.05 (+83.0%)	*NA*

Ref.	Condition	Animal model			Outcomes Quantification: control vs stimulation (% of increase or decrease) at each recorded timepoint		
		Specie	Breed	Modification	Radiologic	Histological and Biochemical	Biomechanical
39	Osteoarthritis	Rat – *Rattus norvegicus*	Sprague Dawley	Unilateral anterior crossbite group (UAC) to model temporomandibular joint osteoarthritis (TMJOA)	*NA*	**At weeks 3 and 6:**<br>Central Third Cartilage Thickness (μm): 212 vs 280 (+32.1%); 186 vs 203 (+9.1%)<br>Proteoglycan positive area (%): 31.5 vs 27.5 (-12.7%); 31.0 vs 52.0 (+67.7%)<br>Type II Collagen-positive area (%): 53 vs 41 (-22.6%); 41 vs 56(%)<br>TNF-α positive cells (%): 50. vs 42.6 (-14.9%); 49.4 vs 34.0 (-31.2%)<br>IL-1β-positive cells (%): 68.0 vs 45.6 (-32.9%); 68.4 vs 50.0 (-26.9%)<br>MMP-13-positive cells (%): 53.8 vs 40.4 (-24.9%); 59.8 vs 55.8 (-6.7%)<br>ADAMTS-5-positive cells (%): 57.6 vs 54.0 (-6.3%); 52.6 vs 43.4 (-17.5%)<br><br>TNF-α (mRNA): 2.4 vs 0.9 (-64.2%); 1.4 vs 0.6 (-54.9%)<br>IL-1β (mRNA): 2.9 vs 1.6 (-44.2%); 1.6 vs 0.8 (-50.3%)<br>IL-6 (mRNA): 2.5 vs 2.1 (-15.0%); 1.6 vs 1.1 (-31.6%)<br>MMP-3 (mRNA): 1.5 vs 1.2 (-20.1%); 2.6 vs 1.7 (-34.1%)<br>MMP-9 (mRNA): 1.1 vs 0.8 (-27.0%); 1.4 vs 0.8 (-41.0%)<br>MMP-13 (mRNA): 1.3 vs 1.2 (-7.5%); 1.5 vs 1.1 (-24.7%)<br>ADAMTS-5 (mRNA): 1.8 vs 1.6 (-12.4%); 1.2 vs 0.8 (-31.1%)<br>COL-X (mRNA): 1.3 vs 0.5 (-64.6%); 2.7 vs 1.1 (-58.6%)<br><br>TNF-α (protein): 1.24 vs 1.00 (-19.4%); 1.16 vs 0.98 (-15.6%)<br>IL-1β (protein): 0.51 vs 0.49 (-3.9%); 0.55 vs 0.53 (-2.6%)<br>MMP-13 (protein): 1.26 vs 1.26 (0.0%); 1.33 vs 1.14 (-14.0%)<br>ADAMTS-5 (protein): 1.27 vs 1.29 (+1.6%); 1.12 vs 1.17 (+4.5%)	*NA*

Ref.	Condition	Animal model			Outcomes Quantification: control vs stimulation (% of increase or decrease) at each recorded timepoint		
		Specie	Breed	Modification	Radiologic	Histological and Biochemical	Biomechanical
40	Osteoarthritis	Rat – *Rattus norvegicus*	Sprague Dawley	Treated with low-dose monosodium iodoacetate (MIA) to induce knee OA	**At week 4:** BMD (mg/cm^3): 687.96 vs 656.98 (-4.5%); BV/TV: 40.39% vs 47.63% (+1.18-fold); BS/BV: 22.22% vs 20.38% (0.92-fold); Tb.Th (μm): 85.08 vs 100.85 (+18.5%); Tb.N (1/mm): 4.57 vs 4.88 (+6.8%); Tb.S (μm): 127.42 vs 110.10 (-13.6%)	**At weeks 4, 8, 12:** Serum OC (ng/mL): 7.4 vs 9.2 (+24.3%), 6.6 vs 10.5 (+59.1%), 8.0 vs 12.6 (+57.5%), average (+47%) PIIANP (ng/mL): 158 vs 208 (+31.6%), 121 vs 196 (+62.0%), 154 vs 164 (+6.5%), average (+33.4%); CTX-I (ng/mL): 8.6 vs 7.6 (-11.6%), 4.6 vs 4.2 (-8.7%), 3.1 vs 3.0 (-3.2%), average (-7.8%); CTX-II (ng/mL): 8.4 vs 5.8 (-31.0%), 6.5 vs 5.0 (-23.1%), 5.4 vs 5.1 (-5.6%), average (-19.9%)	*NA*
41	Osteoarthritis	Rat – *Rattus norvegicus*	Sprague Dawley	Treated with low dose monosodium iodoacetate (MIA) to induce knee OA	**In medial and lateral tibia, and medial and lateral femur:** BV/TV: 36.0% vs 49.6% (+1.37-fold); 34.0% vs 38.8% (+1.14-fold); 45.6% vs 52.0% (+1.14-fold); 45.2% vs 48.0% (+1.06-fold); BS/BV (1/mm): 20.6 vs 18.2 (-11.7%); 27.4 vs 25.2 (-8.0%); 22.2 vs 20.2 (-9.0%); 22.4 vs 21.2 (-5.4%); Tb.Th (μm): 88 vs 120 (+36.4%); 78 vs 83 (+6.4%); 86 vs 104 (+20.9%); 87 vs 98 (+12.6%); Tb.N (1/mm): 4.2 vs 4.4 (+4.7%); 4.4 vs 4.9 (+11.4%); 5.0 vs 5.1 (+1.02%); 4.8 vs 5.3 (+10.4%); Tb.S (μm): 145 vs 112 (-22.8%); 152 vs 123 (-19.1%); 120 vs 94 (-21.7%); 118 vs 94 (-20.3%)	Wnt3a (mRNA): 0.74 vs 1.64 (+121.6%) LRP5 (mRNA): 0.86 vs 1.21 (+40.7%) β-catenin (mRNA): 0.88 vs 1.14 (+29.5%) RANKL (mRNA): 1.34 vs 1.32 (-1.5%) OPG (mRNA): 0.63 vs 1.18 (+87.3%) OPG/RANKL: 0.54 vs 1.01 (+87.0%)	*NA*
42	Osteoarthritis	Guinea Pig – *Cavia porcellus*	Dunkin Hartley	Spontaneously developed degenerative joint diseases (knee OA)	**In medial and lateral tibia plateau, and medial and lateral femoral condyle:** BV/TV: 75.63% vs 74.97% (0.99-fold); 66.69% vs 65.71% (0.98-fold); 78.23% vs 77.13% (0.99-fold); 72.32% vs 68.52% (0.95-fold) Tb.Th (μm): 186.75 vs 185.10 (-0.9%); 174.19 vs 170.36 (-2.2%); 196.63 vs 191.52 (-2.6%); 179.86 vs 168.96 (-6.1%) Tb.N (1/mm): 4.12 vs 4.06 (-1.5%); 3.87 vs 3.90 (+0.8%); 4.12 vs 3.97 (-3.6%); 4.07 vs 4.08 (+0.2%) Tb.S (μm): 62.59 vs 64.55 (+3.1%); 92.40 vs 92.73 (+0.4%); 53.84 vs 58.75 (+9.1%); 71.55 vs 79.51 (+11.1%)	**In medial and lateral tibia plateau, and medial and lateral femoral condyle:** Cartilage histological score (CHS): 10.9 vs 3.9 (-64.2%), 6.4 vs 1.6 (-75.0%), 7.4 vs 2.5 (-66.2%), 5.9 vs 1.8 (-69.5%) Cartilage surface fibrillation index (FI): 127 vs 104 (-18.1%), 104 vs 101 (-2.9%), 108 vs 102 (-5.6%), 103 vs 103 (0%); average (-6.65%) Subchondral bone thickness (SBT): 284.0 vs 242.2 (-14.7%), 304.3 vs 265.4 (-12.8%), 325.0 vs 271.0 (-16.6%), 306.5 vs 261.7 (-14.6%); average (-14.7%) Cartilage thickness: 167.4 vs 265.6 (+58.7%), 153.5 vs 162.9 (+6.1%), 161.4 vs 155.4 (-3.7%), 148.0 vs 147.7 (-0.2%); average (+15.2%)	*NA*

Ref.	Condition	Animal model			Outcomes Quantification: control vs stimulation (% of increase or decrease) at each recorded timepoint		
		Specie	Breed	Modification	Radiologic	Histological and Biochemical	Biomechanical
43	Osteoarthritis	Guinea Pig – *Cavia porcellus*	Dunkin Hartley	Spontaneously developed degenerative joint diseases (knee OA); late knee OA stage	**PEMF 37 Hz, 1.5 mT:**<br>BV/TV: 52.71% vs 50.60% (0.96-fold);<br>Tb.Th (µm): 59.68 vs 68.35 (+14.5%);<br>Tb.N (1/mm): 9.27 vs 7.55 (-18.6%);<br>Tb.S (µm): 54.86 vs 70.41 (+28.3%)<br><br>**PEMF 75 Hz, 1.5 mT:**<br>BV/TV: 52.71% vs 53.76% (+1.01-fold);<br>Tb.Th (µm): 59.68 vs 77.18 (+29.3%);<br>Tb.N (1/mm): 9.27 vs 7.06 (-23.8%);<br>Tb.S (µm): 54.86 vs 68.39 (+24.7%)	**PEMF 37 Hz, 1.5 mT:**<br>CHS: 8.775 vs 6.9 (-21.4%);<br>FI: 143% vs 133% (0.93-fold);<br>SBT (µm): 457.5 vs 344 (-24.8%);<br>CT (µm): 197.5 vs 205.75 (+4.2%)<br><br>**PEMF 75 Hz, 1.5 mT:**<br>CHS: 8.775 vs 5.6 (-36.2%);<br>FI: 143% vs 116.5% (0.81-fold);<br>SBT (µm): 457.5 vs 298 (-34.9%);<br>CT (µm): 197.5 vs 238.25 (+20.6%)	*NA*
44	Bone fracture	Rabbit – *Oryctolagus cuniculus*	New Zealand White	Osteotomy (tibia mid-shaft) stabilized with external fixators, distracted 0.25 mm twice daily for 21 days	**At days 9, 16, 23:**<br>Length of distraction gaps (mm):<br>9.92 vs 9.88 (-0.4%), 9.61 vs 9.43 (-1.9%), 9.86 vs 9.78 (-0.8%)	*NA*	**At days 9, 16, 23:**<br>Torque (Nm): 1.74 vs 2.65 (+52.3%), 2.56 vs 3.70 (+44.5%), 3.39 vs 4.50 (+32.7%)<br>[intact bone torque 4.10]<br>Bone stiffness (Nm/Deg): 0.16 vs 0.21 (+31.3%), 0.2 vs 0.27 (+35.0%), 0.24 vs 0.32 (+33.3%)<br>[intact bone stiffness 0.29]
45	Osteotomy (Abstract only)	Rabbit – *Oryctolagus cuniculus*	New Zealand White	Fibular osteotomy	*NA*	*NA*	*NA*
46	Osteotomy	Rabbit – *Oryctolagus cuniculus*	New Zealand White	Tibial osteotomy	**At days 14 and 21: (exposure 3 h/day)**<br>**PEMF** - callous area (mm^2):<br>0.68 vs 0.79 (+16.1%), 0.72 vs 0.93 (+29.1%)<br>**CMF** - callous area (mm^2)<br>0.68 vs 0.75 (+10.3%), 0.72 vs 0.93 (+29.1%)<br><br>**At days 14 and 21: (exposure 6 h/day)**<br>**PEMF** - callous area (mm^2):<br>0.65 vs 0.82 (+26.2%), 0.72 vs 0.84 (+16.7%)<br>**CMF** - callous area (mm^2):<br>0.65 vs 0.81 (+24.6%), 0.72 vs 0.81 (+12.5%)	*NA*	**At days 14 and 21: (exposure 6 h/day)**<br>**PEMF** - Torque (Nm): 0.93 vs 1.77 (+90.3%), 2.10 vs 3.14 (+49.5%); average (+69.9%)<br><br>Stiffness (Nm/Deg): 0.10 vs 0.16 (+60.0%), 0.18 vs 0.24 (+33.3%); average (+46.7%)<br><br>**CMF -** Torque (Nm): 0.93 vs 1.58 (+69.9%), 2.10 vs 2.96 (+41.0%); average (+55.5%)<br><br>Stiffness (Nm/Deg): 0.10 vs 0.15 (+50.0%), 0.18 vs 0.20 (+11.1%); average (30.6%)
47	Bone defect	Rabbit – *Oryctolagus cuniculus*	New Zealand White	Femoral condylar defects (diameter 5 mm; depth 6 mm); intramedullary implants	**PEMF:**<br>BV/TV: 0.21% vs 0.3% (+1.43-fold);<br>Tb.Th (mm): 0.15 vs 0.25 (+66.7%);<br>Tb.N (n/mm^2): 1.6 vs 1.75 (+9.4%);<br>Tb.S (µm): 500 vs 475 (-5.0%)<br>**CMF:**<br>BV/TV: 0.21 vs 0.39 (+1.86-fold);<br>Tb.Th (mm): 0.15 vs 0.175 (+16.7%);<br>Tb.N (n/mm^2): 1.6 vs 2 (+25.0%);<br>Tb.S (µm): 500 vs 400 (-20.0%)	*NA*	*NA*

Ref.	Condition	Animal model			Outcomes Quantification: control vs stimulation (% of increase or decrease) at each recorded timepoint		
		Specie	Breed	Modification	Radiologic	Histological and Biochemical	Biomechanical
48	Bone defect	Rabbit – *Oryctolagus cuniculus*	New Zealand White	Lateral knee arthrotomy in the loading area of both medial femoral condyles. One defect filled with a scaffold of heterologous equine collagen-I, and the other with the scaffold seeded with bone marrow concentrate (BMC).	*NA*	Niederauer score: 5 vs 6 (+20%)<br>Modified O'Driscoll score (cartilage/bone): 17.8 vs 21 (+18%), 5 vs 9 (+80%)	*NA*
49	Implant	Rabbit – *Oryctolagus cuniculus*	Japanese White	Rough-surfaced dental implant in femurs	*NA*	**At week 2 (8 h/day):**<br>**For 0.2 mT:**<br>BIC: 28.1% vs 53.8% (+1.92-fold);<br>Bone area: 33.4% vs 56.7% (+1.70-fold)<br>**For 0.3 mT:**<br>BIC: 28.1% vs 53.4% (+1.90-fold);<br>Bone area: 33.4% vs 51.9% (+1.55-fold)<br>**For 0.8 mT:**<br>BIC: 28.1% vs 37.7% (+1.34-fold);<br>Bone area: 33.4% vs 34.5% (+1.03-fold)	*NA*
50	Implant	Rabbit – *Oryctolagus cuniculus*	New Zealand White	Custom Ti-6Al-4V dental implants in mandible	*NA*	**In labial surfaces of the mandibular bone, at weeks 2 and 8:**<br>Osteoblast number: 9.85 vs 11.42 (+15.9%), 12.57 vs 30.85 (+145.4%),<br>Trabecular bone: 6.42 vs 9.85 (+53.4%), 6.42 vs 32.28 (+402.8%),<br>Fibrous Tissue: 3.28 vs 3.28 (+0.0%), 1.20 vs 2.71 (+125.8%)<br><br>**In lingual surfaces of the mandibular bone, at weeks 2 and 8:**<br>Osteoblast number: 9.57 vs 11.0 (+14.9%), 9.14 vs 30.28 (+231.3%)<br>Trabecular bone: 5.85 vs 7.71 (+31.8%), 5.85 vs 31.28 (+434.7%)<br>Fibrous Tissue: 2.71 vs 3.14 (+15.9%), 2.71 vs 2.00 (-26.2%)	*NA*
51	Implant	Rabbit – *Oryctolagus cuniculus*	New Zealand White	Titanium implants in proximal metaphyses of tibia	**At weeks 2 and 4:**<br>BV/TV: +56%, +69%; average (+62.5%)<br>Tb.Th: +7.69%, +16.78%; average (+12.2%)<br>Tb.N: +37%, +34%; average (+35.5%)<br>Tb.S: -29.16%, -28.94%; average (-29.1%)<br>Connectivity density (Conn.D): +73%, +13.23%; average (+43.1%)	**At weeks 2 and 4:**<br>BIC: 54.2% vs 74.3% (+1.37-fold); 70% vs 85.3% (+1.22-fold); average (+1.30-fold)	*NA*

Ref.	Condition	Animal model			Outcomes Quantification: control vs stimulation (% of increase or decrease) at each recorded timepoint		
		Specie	Breed	Modification	Radiologic	Histological and Biochemical	Biomechanical
52	Implant	Rabbit – *Oryctolagus cuniculus*	New Zealand White	Intramedullary Kirshner wire inserted in the humerus	*NA*	Bone formation rate (x10^{-2} cm^2): 3.9 vs 8.9 (+128.2%)<br><br>**At days 1, 3, 5, 7 and 14:**<br>ALP activity (BLU/mg): 21.0 vs 22.0 (+4.8%), 22.5 vs 21.0 (-6.7%),23.5 vs 22.0 (-6.4%), 30.0 vs 33.0 (+10.0%), 30.0 vs 87.0 (+190.0%)<br>Osteoblast proliferation activity (AgNOR counts): 1.56 vs 1.38 (-11.5%), 1.66 vs 1.88 (+13.3%), 1.90 vs 2.18 (+14.7%), 1.90 vs 3.16 (+66.3%), 2.08 vs 3.38 (+62.5%)	*NA*
53	Implant (Abstract only)	Rabbit – *Oryctolagus cuniculus*	Japanese White	Porous bead-covered titanium implants inserted into the humerus diaphysis	*NA*	*NA*	*NA*
54	Implant	Rabbit – *Oryctolagus cuniculus*	New Zealand White	Intramedullary Kirshner wire implant in the medullary canal of femur and tibia	*NA*	**Implants on femur (wo/ motion; w/ motion):**<br>New bone area (mm^2): 0.01 vs 0.01 (+0.0%), 0.58 vs 1.08 (+86.2%),<br>New bone area: 0.07% vs 0.04% (0.57-fold), 4.81% vs 6.94% (+1.44-fold),<br>New bone surface (mm): 0.53 vs 0.38 (-28.3%), 23.4 vs 41.5 (+77.4%),<br>Canal area (mm^2): 13.1 vs 14.8 (+13.0%), 13.5 vs 16.4 (+21.5%),<br>Whole bone area (mm^2): 32.4 vs 32.9 (+1.5%), 34.2 vs 36.3 (+6.1%),<br>Relative canal size: 40.7% vs 45.0% (+1.1-fold), 39.4% vs 45.1% (+1.14-fold),<br>Cortex area (mm^2): 19.3 vs 18.1 (-6.2%), 20.7 vs 19.8 (-4.3%),<br><br>**Implants on tibia (wo/ motion; w/ motion):**<br>New bone area (mm^2): 0.10 vs 0.01 (-90.0%), 0.33 vs 0.38 (+15.2%)<br>New bone area:1.43% vs 0.11% (0.08-fold), 4.80% vs 5.44% (+1.13-fold)<br>New bone surface (mm): 3.98 vs 0.37 (-90.7%), 13.9 vs 14.0 (+0.7%)<br>Canal area (mm^2): 6.29 vs 7.17 (+14.0%), 6.95 vs 7.01 (+0.9%)<br>Whole bone area (mm^2): 21.7 vs 22.8 (+5.1%), 24.0 vs 23.0 (-4.2%)<br>Relative canal size: 29.1% vs 31.4% (+1.08-fold), 28.9 vs 30.9 (+1.07-fold)<br>Cortex area (mm^2): 15.4 vs 15.6 (+1.3%), 17.1 vs 16.0 (-6.4%)	*NA*

Ref.	Condition	Animal model			Outcomes Quantification: control vs stimulation (% of increase or decrease) at each recorded timepoint		
		Specie	Breed	Modification	Radiologic	Histological and Biochemical	Biomechanical
55	Implant	Rabbit – *Oryctolagus cuniculus*	New Zealand White	Femoral porous titanium (pTi) implants	**At weeks 6 and 12:**<br>BS/BV: -36.2%, -43.6%; average (-39.9%)<br>BV/TV: +78.5%, +88.0%; average (+83.3%)<br>Tb.N: +21.7%, +16.3%; average (+19%)<br>Tb.Th: +41.8%, +49.6%; average (+45.7%)<br>Tb.S: -30.7%, -42.8%; average (-36.8%)	**At weeks 6 and 12:**<br>Bone area fraction: +143.1%, +169.8%; average (+156.5%)<br>Mineral apposition rate: +62.7%, +96.3%; average (+79.5%)<br>Mineralizing surface per bone surface (MS/BS): +85.6%, +85.6%; average (+85.6%)<br>Bone formation rate per bone surface (BFR/BS): +209.3%, +239.9%; average (+224.6%)<br><br>Runx2 (mRNA): +48%, +160%; average (+104%)<br>BMP2 (mRNA): +270%, +168%; average (+219%)<br>OC (mRNA): +129%, +112%; average (+120.5%)<br>Wnt1 (mRNA): +74%, +386%; average (+230%)<br>Lrp6 (mRNA): +382%, +176%; average (+279%)<br>β-catenin (mRNA): +104%, +876%; average (+490%)	*NA*
56	Implant	Rabbit – *Oryctolagus cuniculus*	New Zealand White	Tricalcium phosphate (TCP) or HA rod implants in tibia	*NA*	**Assessments in cortical window area and medullary cavity area: (week 3)**<br><br>Bone fraction: [HA] 12.7% vs 23.2% (+1.83-fold), 5.1% vs 7.8% (+1.53-fold);<br>[TCP] 9.5% vs 10.4% (+1.09-fold), 8.0% vs 12.1% (+1.51-fold)<br>Implant fraction: [HA] 32.2% vs 31.5% (0.98-fold), 41.0% vs 29.1% (0.70-fold);<br>[TCP] 49.7% vs 44.7% (0.90-fold), 49.0% vs 48.6% (0.99-fold)<br>Soft tissue fraction: [HA] 55.1% vs 45.3% (0.82-fold), 53.9% vs 63.1% (+1.17-fold);<br>[TCP] 40.8% vs 48.8% (+1.20-fold), 43.0% vs 49.2% (+1.14-fold)<br>Boundary fraction: [HA] 28.1% vs 59.0% (+2.10-fold). 21.6% vs 34.1% (+1.58-fold);<br>[TCP] 31.4% vs 30.2% (0.96-fold), 29.0% vs 32.9% (+1.13-fold)<br>Tb.Th (μm): [HA] 28.5 vs 42.2 (+48.1%), 19.7 vs 28.4 (+44.2%);<br>[TCP] 20.1 vs 23.5 (+16.9%), 21.6 vs 22.2 (+2.8%)	*NA*
57	Implant	Rabbit – *Oryctolagus cuniculus*	New Zealand White	Natural or synthetic HA rod implants in tibia	*NA*	*NA*	*NA*

Ref.	Condition	Animal model			Outcomes Quantification: control vs stimulation (% of increase or decrease) at each recorded timepoint		
		Specie	Breed	Modification	Radiologic	Histological and Biochemical	Biomechanical
58	Implant	Rabbit – *Oryctolagus cuniculus*	New Zealand White	Hydroxyapatite implant in femoral condyles	**Week 3:**<br>BV/TV: 40.68% vs 31.82% (0.78-fold)<br>BS/TV: 2.55% vs 2.31% (0.91-fold)<br>Tb.Th (μm): 38.96 vs 35.05 (-10.0%)<br>Tb.N (1/mm) 10.62 vs 9.65 (-9.1%)<br>Tb.S (μm^2): 57.37 vs 89.38 (+55.8%)<br><br>**Week 6:**<br>BV/TV: 33.94% vs 34.29% (+1.01-fold)<br>BS/TV: 2.35% vs 1.48% (0.63-fold)<br>Tb.Th (μm): 40.48 vs 55.25 (+36.5%)<br>Tb.N (1/mm) 9.80 vs 6.17 (-37.0%)<br>Tb.S (μm^2): 77.12 vs 123.92 (+60.7%)	**At weeks 3 and 6:**<br>Affinity index: 60.0% vs 88.3% (+1.47-fold); 75.0% vs 87.3% (+1.16-fold)	**Microhardness (0.2, 0.5, 1.0, 2.0 mm from implant interface):**<br>**Week 3:** 46.33 vs 69.18 (+49.3%),<br>46.15 vs 66.47 (+44.0%),<br>60.72 vs 61.50 (+1.3%),<br>65.55 vs 77.65 (+18.5%);<br><br>**Week 6:** 51.83 vs 61.75 (+19.1%),<br>54.20 vs 62.25 (+14.9%),<br>61.33 vs 61.90 (+0.9%),<br>63.28 vs 65.03 (+2.8%)
59	Implant	Rabbit – *Oryctolagus cuniculus*	New Zealand White	HA rod implants in femur	*NA*	**At weeks 3 and 6:**<br>Affinity index: 54.9% vs 61.0% (+1.11-fold),<br>53.5% vs 63.6% (+1.19-fold)<br>MAR (μm/day): 3.39 vs 5.64 (+66.4%),<br>4.24 vs 4.36 (+2.8%)<br>BFR/BS ($\mu m^2/\mu m$/day): 1.48 vs 1.54 (+4.1%),<br>1.15 vs 1.46 (+27.0%)	**At weeks 3 and 6:**<br>Microhardness (0.2 mm from implant interface; HV_{200}): 49.7 vs 67.6 (+36.0%),<br>55.6 vs 54.2 (-2.5%)<br>BMI: 54% vs 77% (+1.43-fold),<br>58% vs 70% (+1.21-fold)<br>Maximum push-out force (Fmax)(N):<br>87 vs 234 (+169.0%), 132 vs 167 (+26.5%)<br>Ultimate shear strength (σu)(MPa):<br>6.3 vs 14.6 (+131.7%), 7.9 vs 8.9 (+12.7%)
60	Implant	Rabbit – *Oryctolagus cuniculus*	New Zealand White	Pure dental titanium implant fixtures in tibiae metaphysis	*NA*	*NA*	**At days 21 and 42:**<br>Removal Torque: 8.87 vs 9.42 (+6.2%), 14.00 vs 14.38 (+2.7%)
61	Implant	Rabbit – *Oryctolagus cuniculus*	New Zealand White	Porous titanium (pTi) implants in hindlimb bone defect; type 1 diabetes mellitus induced through alloxan injection	BV/TV: +70.3%;<br>Tb.Th: +33.2%;<br>Tb.N: +54.7%;<br>Tb.S: -16.6%;<br>Ct.Th: +15.2%;<br>Ct.Ar: +14.9%;<br>Ct.Po: -25.2%	Peri-implant bone area: +3.71-fold;<br>Trabecular bone area: +85.6%;<br>Cancellous bone MAR: +57.8%;<br>N.Ob/BS: +87.3%;<br>Ob.S/BS: +98.8%,	Tibial maximum load (N): 247 vs 290 (+17.4%);<br>Stiffness (N/mm): 361 vs 416 (+15.2%);<br>Peri-implant bone modulus: +25.7%;<br>Peri-implant bone hardness: +24.0%;<br>Cortical bone modulus: +26.3%;<br>Cortical bone hardness: +39.6%

Ref.	Condition	Animal model			Outcomes Quantification: control vs stimulation (% of increase or decrease) at each recorded timepoint		
		Specie	Breed	Modification	Radiologic	Histological and Biochemical	Biomechanical
62	Implant; Bone Maintenance (glucocorticoid degeneration)	Rabbit – *Oryctolagus cuniculus*	New Zealand White	Glucocorticoid treatment (dexamethasone); Porous titanium (pTi) implants in hindlimb bone defect;	BMD: +32.1%; BV/TV: +68.3%; Tb.N: +32.0%; Tb.Sp: -22.9%; Ct.Th: +19.2%; Ct.Ar: +28.2%; Conn.D: +28.2%; SMI: -18.8%; BS/BV: -19.8%	Peri-implant bone: +3.52-fold; Cancellous bone: +68.8% Peri-implant bone MAR: +66.4%; Peri-implant bone BFR/BS: +126.4%; Trabecular bone MAR: +46.3%; Trabecular bone BFR/BS +114.9%; Serum OC: +39.6%; P1NP: +40.7%; TRACP5b: -4.3%; CTX-1: -3.9%; Empty lacunae: -44.2%; TUNEL-positive osteocytes: -52.4%	Peri-implant bone modulus: +29.9%; Peri-implant bone hardness: +22.0%; Cortical bone modulus: +22.9%; Cortical bone hardness: +28.7%
63	Soft Tissue Healing	Rabbit – *Oryctolagus cuniculus*	New Zealand White	Patellectomy	*NA*	**At week 8 and 16:** Newly formed bone: +99.2%, +97.8%; Regenerated fibrocartilage zone: +41.9%, +22.8%; Proteoglycans: +36.9% (week 16); Fibrocartilage cells: -28.6% (week 16)	**At week 8 and 16:** Load to failure (J): 131.4 vs 182.6 (+39.0%), 247.1 vs 311.0 (+25.9%); Ultimate strength (MPa): 4.35 vs 6.83 (+57.0%), 6.84 vs 8.46 (+23.7%); Energy to failure (N): 0.22 vs 0.34 (+54.5%), 0.52 vs 0.87 (+67.3%)
64	Bone fracture	Dog – *Canis lupus familiaris*	Beagle	Fibular osteotomy	*NA*	*NA*	**For deformations of 0.025, 0.050 and 0.075 inches (65 Hz stimuli):** Load values (g): 21 vs 29 (+38.1%), 42 vs 64 (52.4%), 63 vs 135 (+114.3%)
65	Late bone healing phase	Dog – *Canis lupus familiaris*	Mixed breed	Tibial osteotomy	**At weeks 2, 4, 6, 8, 10, 12:** Callus area (mm^2): 7.3 vs 6.2 (-15.1%), 15.5 vs 26.9 (+73.5%), 34.1 vs 51.7 (+51.6%), 42.3 vs 67.0 (+58.4%), 43.0 vs 69.9 (+62.6%), 36.7 vs 68.1 (+85.6%)	**Histological Score:** Bone: 30.1% vs 49.2% (+1.63-fold.5%); Cartilage: 0.7% vs 0.5% (0.71-fold); Fibrous Tissue: 0.3% vs 0.4% (+1.33-fold)	Load-Bearing (4, 8, 12 weeks): 28.7% vs 41.9% (+1.46-fold), 31.9% vs 59.8% (+1.87-fold), 77.8% vs 71.9% (0.92-fold); Torque (N.m) (12 weeks): 18.6 vs 22.4 (+20.4%); Torsional stiffness (N/rad) (12 weeks): 89.8 vs 138.8 (+54.6%)
66	Spine fusion	Dog – *Canis lupus familiaris*	Beagle	L5-L6 destabilization followed by posterolateral spinal fusion	**At L6 vertebral body and posterior fusion mass:** BMD (mg/cm^3): 391.04 vs 438.48 (+12.0%), 695.53 vs 744.60 (+7.0%)	*NA*	**Stiffness assessments:** Compression (KN/mm): 3.2 vs 2.6 (-18.8%); Flexion (KNmm/degree): 36.7 vs 39.2 (+6.8%); Lateral bend (KNmm/degree): 183.3 vs 138.9 (-24.2%); Torsion (Nm/degree): 1.2 vs 1.1 (-8.3%)

Ref.	Condition	Animal model			Outcomes Quantification: control vs stimulation (% of increase or decrease) at each recorded timepoint		
		Specie	Breed	Modification	Radiologic	Histological and Biochemical	Biomechanical
67	Osteoporosis	Dog – *Canis lupus familiaris*	Beagle	OVX	*NA*	Bone loss (%):<br>23.1% vs 9.5% (0.41-fold)<br>Cross-sectional area (mm^2):<br>5.72 vs 6.31 (+10.3%)<br>Residual diameter osteonal canal:<br>19.7 vs 21.7 (+10.2%)<br>Osteonal radial closure rate (μm/day):<br>1.7 vs 1.8 (+5.9%)<br><br>**At week 0 and 7:**<br>Active osteons (n/mm^2):<br>1.1 vs 1.3 (+18.2%), 3.6 vs 4.7 (+30.6%)	*NA*
68	Osteoarthritis	Dog –	ND	Three cartilage defects on the stifle joint (equivalent to knee), followed by tissue-engineered (TE) repair using osteochondral grafts	*NA*	OARSI cartilage score: 19.0 vs 18.5 (-2.6%; PEMF-stimulated repairs -70% likely to have worse score)<br>Structure: 4.0 vs 4.0 (0.0%)<br>Collagen: 3.0 vs 3.0 (0.0%)<br>Tidemark: 2.0 vs 2.0 (0.0%)<br>Bone: 3.0 vs 3.0 (0.0%)<br>Chondrocytes: 4.0 vs 3.5 (-12.5%; PEMF-stimulated repairs -60% likely to have chondrocyte pathology)<br>Proteoglycans: 3.0 vs 3.0 (0.0%; PEMF-stimulated repairs -80% likely to have proteoglycan pathology)<br><br>Osteochondral Allograph integration:<br>7.0 vs 6.0 (-14.3%)<br>Fill: 2.0 vs 2.0 (0.0%)<br>Edge integrity: 1.0 vs 1.0 (0.0%)<br>Surface congruity: 2.0 vs 1.0 (-50%)<br>Fibrosis: 1.0 vs 1.0 (0.0)<br>Inflammation: 1.0 vs 1.0 (0.0%)<br><br>**Functional scores change from baseline:**<br>Clinical lameness: 1.3 vs 1.3 (0.0)<br>Functional gait: -1.5 vs -1.7 (+13.3%)<br>Comfortable range of motion:<br>-7.5 vs -10.8 (+44.0%)<br>Pain: 1.3 vs 1.1 (-15.4%)<br>Effusion: 1.3 vs 1.7 (+30.8%)<br>Total pressure index: -2.8 vs -2.1 (-25.0%)	*NA*

Ref.	Condition	Animal model			Outcomes Quantification: control vs stimulation (% of increase or decrease) at each recorded timepoint		
		Specie	Breed	Modification	Radiologic	Histological and Biochemical	Biomechanical
69	Spine fusion	Dog – *Canis lupus familiaris*	Mixed breed	L1-2 and L4-5 facet fusion	*NA*	*NA*	*NA*
70	Implant	Dog – *Canis lupus familiaris*	Mixed breed	Dental implants placed in the mandible	*NA*	*NA*	*NA*
71	Non-union (Abstract only)	Dog – *Canis lupus familiaris*	Beagle	Osteotomy	*NA*	*NA*	*NA*
72	Osteotomy (Abstract only)	Dog – *Canis lupus familiaris*	Mixed breed	*ND*	*NA*	*NA*	*NA*
73	Osteotomy	Sheep – *Ovis aries*	Black face	Tibial osteotomy	*NA*	*NA*	*NA*

Supplementary Table 3 - Inductive coupling stimulation characteristics and biological outcomes evaluated in human patients suffering from musculoskeletal disorders or submitted to orthopedic surgeries. Data was collected from 40 studies and includes the evaluated musculoskeletal disorder/condition, applied complementary procedures, the stimuli characteristics (Type of stimulation, used device, stimuli waveform, frequency, periodicity, magnetic field strength, exposure time, and assay duration), and respective radiologic, histological or biochemical, and biomechanical outcomes. The evaluated disorders include bone defects (delayed and non-unions, and bone fractures), congenital pseudarthrosis, osteoporosis, osteoarthritis and soft tissue damage, and the evaluated orthopedic surgeries were spine fusions, implant osseointegration and limb lengthening. ND – not defined stimuli characteristic; NA – not applicable; AC – alternating voltage; CV - constant voltage; BMD – bone mineral density; ALP – Alkaline phosphatase; HA – hydroxyapatite; PEMF – Pulsed Electromagnetic Field; CMF - Combined Magnetic Field.

Ref	Condition	Procedure	Stimuli characteristics						Outcomes		
			Type of stimulation<br><br>Stimulation device<br><br>Waveform	Frequency (Hz)	Periodicity	MF strength (mT)	Exposure time (h)	Assay duration (months)	Radiologic	Histological and Biochemical	Other Clinical Outcomes
74	Non-union	All patients treated with CMF until union	CMF (OrthoLogic 1000™ (OL 1000))	*ND*	*ND*	*ND*	0.5	9	CMF technology healed 78% of tibial fracture non-union and 45% of all fracture non-union.	*NA*	Pain at rest, with stress, and on weight bearing decreased following treatment with CMF, with no adverse events reported.
75	Non-union	All patients treated with CMF until union	CMF (OrthoLogic 1000™ (OL 1000))	*ND*	*ND*	*ND*	0.5	9	Patients treated earlier than 6 months post-injuty with CMF demonstrated significantly greater healing rates and shorter mean times to heal than patients treated 6 months or more post-injury.	*NA*	*NA*
76	Non-union	All patients treated with CMF until union	CMF (DJO Global, UK)	*ND*	*ND*	*ND*	0.5	30	CMF was successful to treat non-unions, with an overall success rate of 84% (n=21), and average time to union of 6.62 months.	*NA*	*NA*
77	Non-union (scaphoid)	Thumb spica cast immobilization; PEMF treatment until healing	PEMF (EBI Bone Healing System® Model 420); Pulsed quasi-rectangular electrical signal	15	*ND*	*ND*	8 - 10	2.5 – 9 (avg. 4.3)	The fractures healed in 35 (80%) of the 44 patients. PEMF is a reliable alternative method to treat non-united scaphoid fractures.	*NA*	No pain, no surgical risk, and no known complications. Grip and motion near normal levels (83% and 89%, respectively), comparable with other invasive methods.

Ref	Condition	Procedure	Stimuli characteristics						Outcomes		
			Type of stimulation<br>Stimulation device<br>Waveform	Frequency (Hz)	Periodicity	MF strength (mT)	Exposure time (h)	Assay duration (months)	Radiologic	Histological and Biochemical	Other Clinical Outcomes
78	Non-union	Fixation or immobilization applied when required; treatment until fracture union	PEMF (Orthopulse® I and Orthopulse® II; Ossatec)	15 (pulse)	5 ms pulse bursts: 5 µs pulses every 62 ms	*ND*	24	4.9 – 36.6 weeks (median12.9 weeks)	The success rate of PEMF treatment on non-unions was 76% for the Long Bone Fracture (LBF) group and 79% for the non-LBF group. PEMF stimulation showed to be an effective treatment.	*NA*	PEMF reduced the pain during treatment.
79	Delayed union (long bone)	Treatment until fracture union	PEMF (Orthopulse® II; Ossatec)	15 (pulse)	5 ms pulse bursts: 5 µs pulses every 62 ms	*ND*	8	2 – 12 (avg 4.8)	PEMF treatment for an average of 4.8 months led to a higher healing success rate (77.4%), than the control (48.1%). Early application of PEMF increased rate of union.	*NA*	*NA*
80	Non-union (scaphoid)	Thumb spica cast immobilization; PEMF treatment until healing	PEMF (EBI Bone Healing System® Model 420); Pulsed quasi-rectangular electrical signal	15	*ND*	*ND*	8 - 10	2.5 – 9 (avg. 4)	The overall healing success rate has decreased since the previous review from 80% to 69%. PEMF treatment should be a secondary alternative to bone-grafting procedures.	*NA*	*NA*
81	Non-union	*ND*	PEMF; Quasi-rectangular asymmetrical pulse	15 (pulse)	5 ms pulse bursts	*ND*	12 to 16	3 6 (avg.6)	Union was achieved in 38 out of 53 cases (71.7%)	*NA*	*NA*
82	Non-union	PEMF + anterior iliac crest bone marrow injection or autologous iliac crest bone graft	PEMF	*ND*	*ND*	*ND*	10	avg 4.3 (osteotomy) or 3 (fracture)	All patients in the osteotomy group treated with PEMF alone had their non-union healed in an average of 18.8 weeks. In the fracture group, 57% had non-union healed with PEMF and other treatments in an average of 13.2 weeks.	*NA*	The use of PEMF is a good option for the initial treatment of pediatric non-unions, especially when secondary to osteotomies.

Ref	Condition	Procedure	Stimuli characteristics						Outcomes		
			Type of stimulation<br><br>Stimulation device<br><br>Waveform	Frequency (Hz)	Periodicity	MF strength (mT)	Exposure time (h)	Assay duration (months)	Radiologic	Histological and Biochemical	Other Clinical Outcomes
83	Non-union	*ND*	PEMF (Biomet® EBI Bone Healing System)	15 (pulse)<br>4 444 (burst)	4,5 ms pulse bursts of 225 µs pulses	1.8	patient dependent	patient dependent	Patients treated with the PEMF device for >9 h/day had a significant reduction in time to heal, achieving successful fracture repair an average of 76 days earlier.	*NA*	Median heal time reduced by 35%–60%, depending on fracture characteristics when complying the recommended daily use of 10 h/day.
84	Non-union (metatarsal)	Treatment until union	PEMF (Biomet® EBI Bone Healing System)	15 (pulse)<br>4 444 (burst)	4.5 ms pulse bursts of 225 µs pulses	1.8	10	1.5 – 3.8<br>(avg 2)	PEMF had faster average time to radiographic union compared to unstimulated controls (avg 8.9 vs avg 14.7 weeks).	The adjunctive use of PEMF for fifth metatarsal fracture non-union produced a significant increase in local placental growth factor and multiple other factors, including BDNF and BMP-5 and BMP-7.	*NA*
85	Non-union (tibia)	Long-leg plaster cast immobilization	PEMF (Biomet® EBI Bone Healing System) Quasi-rectangular pulses followed by a sharper reverse form	15 (pulse)<br>4 444 (burst)	15 pulse bursts/s, 20 pulses/burst; 200 µs pulse width and 25 µs pulse interval;	*ND*	12	2.8	Radiological union in 5 fractures, progress to union in 5 but no progress in 10. In the control there was union in 1 fracture and progress towards union in 1 but no progress in 23.	*NA*	*NA*
86	Delayed & Non-union (metatarsi)	Non-weightbearing cast, short leg weightbearing cast, or weightbearing postoperative shoe; PEMF treatment until healing	PEMF	15 (pulse)<br>4 444 (burst)	4,5 ms pulse bursts of 20 pulses with 200 µs and 5 µs pause	0 to 2.0	8 - 10	2 – 4<br>(avg. 3)	All fractures healed in a mean time of 4 months (range 2–8 months). Fractures treated with both PEMF and a non-weightbearing cast healed in a mean time of 3 months (range 2–4 months), which may be an effective alternative treatment.	*NA*	*NA*
87	Non-union (Long bone; tibia)	Application of a Denham external fixator; PEMF treatment until healing	PEMF (St Thomas' electrical bone stimulator); High mark-space ratio rectangular voltage waveform	23.2 (pulse);<br>3700 (burst)	3 ms pulse burst duration and 40 ms interval;14 µs pulse width; 256 µs pulse interval	1.4	12 - 16	3 – 8<br>(avg. 4)	All hypertrophic non-unions united. Two atrophic non-unions failed, but six atrophic non-unions were also treated successfully.	*NA*	*NA*

Ref	Condition	Procedure	Stimuli characteristics						Outcomes		
			Type of stimulation<br><br>Stimulation device<br><br>Waveform	Frequency (Hz)	Periodicity	MF strength (mT)	Exposure time (h)	Assay duration (months)	Radiologic	Histological and Biochemical	Other Clinical Outcomes
88	Non-union (Long bones) (Abstract only)	*ND*	Single pulse PEMF	80	0.3 ms pulse repeating every 12 ms	0.01 to 0.1	*ND*	avg 14 weeks	Within an average treatment period of 14 weeks, 11 of the 13 patients had successful bone healing.	*NA*	*NA*
89	Congenital pseudarthrosis (tibia)	Intramedullary nailing.	PEMF (Howmedica)	75	1.3 us pulse duration	*ND*	>8	3 – 10 (avg 5.6)	91% union in pseudoarthrosis patients treated with PEMF, compared with 83% in unstimulated patients. The average time union of the fractures was 3.3 months (range 2–7 months) with PEMF and 4.9 months (range 3–9 months) without stimulation	*NA*	*NA*
90	Congenital pseudarthrosis (tibia)	Immobilization during the first 6 months	Repetitive single pulse PEMF (ElectroBiology, Inc. (EBI))	*ND*	*ND*	*ND*	10 to 12	4 – 49.2 (avg 16.8)	Episodic use of PEMF proved effective in controlling stress fractures in several patients until puberty.	*NA*	PEMF appears to be an effective, conservative adjunct in the management of congenital lesions.
91	Congenital pseudarthrosis (Tibia)	Boyd's dual onlay grafts and PEMF (Case report; 17-year follow-up)	PEMF	*ND*	*ND*	*ND*	10	44	Bone union achieved with Boyd's dual onlay grafts and PEMF. A radiograph of 7 years after operation indicated rigid bone union and remodelling of the grafted tibial span. An unacceptable degree of leg shortening had been avoided.	*NA*	*NA*

Ref	Condition	Procedure	Stimuli characteristics						Outcomes		
			Type of stimulation<br>Stimulation device<br>Waveform	Frequency (Hz)	Periodicity	MF strength (mT)	Exposure time (h)	Assay duration (months)	Radiologic	Histological and Biochemical	Other Clinical Outcomes
92	Delayed union	Delayed unions after foot and ankle arthrodesis treated with immobilization, limited weightbearing, and PEMF stimulation	PEMF (EBI Bone Healing System®)	15 (pulse)<br>4 444 (burst)	Pulse Bursts: 20 pulses with 200 µs and 25 µs pause	*ND*	*ND*	5 – 27 (median 7)	The protocol of PEMF, immobilization, and limited weightbearing had a relatively low success rate. Successful in 5 of 19 (26%) patients.	*NA*	*NA*
93	Non-union (scaphoid)	Retrograde percutaneous screw fixation	PEMF (Physio-Stim® (Orthofix Inc.))	*ND*	*ND*	0.2	3	4	The results of the study did not show improvements in carpal scaphoid non-union healing rate with PEMF, but PEMF was only applied 6 weeks post-operation, which is considered late.	*NA*	*NA*
94	Bone Fracture (Mandibular)	Maxillo-mandibular fixation (MMF)	PEMF	40	*ND*	1.0	6 (D1);<br>3 (D2-7);<br>1.5 (D8-13)	13 days	There was no significant difference in the mean bone density values. However, the percentage change in bone density revealed that the PEMF group had insignificant decreases at post-surgery day 14 and a significant increase at post-surgery day 28.	*NA*	Mouth opening significantly more stable than the control group with decreased pain.
95	Bone Fracture (Mandibular)	Maxillo-mandibular fixation (MMF)	PEMF (EM-probe Solo device)	72	200 ns pulse duration	*ND*	2	12 days	At 15 days postoperatively, the mean density in the fracture sites decreased by 2.3% on PEMF and by 6% on control. At 30 days, there was a 10.2% increase in PEMF and 1.9% in control, when compared with day 15.	*NA*	Treatment with simultaneous PEMF stimulation and MMF were clinically stable. Pain reduction in comparison with MMF-only group.

Ref	Condition	Procedure	Stimuli characteristics						Outcomes		
			Type of stimulation<br><br>Stimulation device<br><br>Waveform	Frequency (Hz)	Periodicity	MF strength (mT)	Exposure time (h)	Assay duration (months)	Radiologic	Histological and Biochemical	Other Clinical Outcomes
96	Bone Fracture (Mandibular)	Maxillo-mandibular fixation (MMF)	PEMF (EM-probe Solo) vs LILI (low intensity laser irradiation)	72	200 ns pulse duration	*ND*	2	12 days	Significant increase in bone density at the 2nd and 4th postoperative weeks and better bone healing time. Short period MMF supplemented with PEMF is recommended.	*NA*	Insignificant difference was found between the means of bone densities of group A (PEMF) and B (LILI) throughout the study intervals.
97	Bone Fracture (Scaphoid)	Forearm cast immobilization	PEMF (Orthopulse®, Ossatec)	15	5 ms pulse bursts of 5 µs pulses every 62 ms	*ND*	24	1.5 and 3	Time to radiological union did not differ significantly between groups.	*NA*	Functional and clinical outcome did not differ significantly between groups.
98	Bone Fracture (Scaphoid)	Forearm cast immobilization	PEMF (Orthopulse®, Ossatec)	15	5 ms pulse bursts of 5 µs pulses every 62 ms	*ND*	24	1.5	Consolidation assessed in the three different stages, revealed no significant differences between the intervention and the placebo group at any of the stages	*NA*	No significant differences regarding wrist movement, except at 24 weeks, when wrist movement in control was slightly higher
99	Bone fracture (tibia)	*ND*	PEMF (Biomet® EBI Bone Healing System)	15 (pulse)<br>4 444 (burst)	4.5 ms pulse bursts of 225 µs pulses	1.8	10	2.75	PEMF did not improve radiographic union of acute tibial shaft fractures.	*NA*	Adjuvant PEMF stimulation did not prevent secondary surgical interventions for delayed union or non-union or patient-reported functional outcomes.
100	Bone Healing	High Tibial Osteotomy	ELF-PEMF (Extremely low-frequence; Somagen®)	16	---	6 to 282 µT	7 minutes	1	Faster osseous consolidation for ELF-PEMF compared to placebo treatment, but not statistically significative (ELF-PEMF group 4.64±0.19%/week vs placebo group 4.18±0.13%/week)	ALP serum levels (osteoblast function) significantly increased and TRAP5b serum levels (osteoclast function), were not significantly different, in the ELF-PEMF.	ELF-PEMF treatment may support osseous consolidation in elder patients.

Ref	Condition	Procedure	Stimuli characteristics						Outcomes		
			Type of stimulation<br><br>Stimulation device<br><br>Waveform	Frequency (Hz)	Periodicity	MF strength (mT)	Exposure time (h)	Assay duration (months)	Radiologic	Histological and Biochemical	Other Clinical Outcomes
101	Bone Fracture (distal radius)	Prospective, double-blind, randomized, and sham-controlled study using PEMF applied at the fracture site immediately after open reduction and internal fixation (ORIF) surgery.	PEMF (Pulsar Medtech Ltd., Israel)	10 (pulse); 20000 (burst)	*ND*	0.05 to 0.5	24	2.75	Patients treated with active PEMF demonstrated a significantly higher extent of union at 4 weeks, assessed by CT	*NA*	PEMF accelerated bone healing (union bridging and hand grip strength). Mean early hand grip strength in the active group was significantly higher than the control group.<br>PEMF resulted in improvement of the PRWE function subscale.<br>No differences in functionality or mental health, nor adverse effects related directly to the device were reported.
102	Spine fusion (cervical; non-union)	Anterior cervical discectomy and fusion (ACDF)	PEMF (CervicalStim™)	*ND*	*ND*	*ND*	4	3	At 6 months, PEMF group had a significantly higher fusion rate than the control group. At 12 months, there were no significant differences.	*NA*	PEMF stimulation is safe in this clinical setting (no adverse effects).
103	Spine fusion (lumbar)	*ND*	PEMF	*ND*	*ND*	*ND*	8	12	In the active group there was a 92% success rate, while the control group had a 65% success rate.	*NA*	*NA*
104	Spine fusion (lumbar/sacral) (Abstract only)	One-level or two-level fusions (between L3 and S1) without instrumentation	CMF	*ND*	*ND*	*ND*	0.5	9	CMF led to 64% healed at 9 months compared with 43% of patients with placebo devices. CMF treatment of 30 min/day increases the probability of successful spine fusion, accelerating the healing process.	*NA*	*NA*

Ref	Condition	Procedure	Stimuli characteristics						Outcomes		
			Type of stimulation<br><br>Stimulation device<br><br>Waveform	Frequency (Hz)	Periodicity	MF strength (mT)	Exposure time (h)	Assay duration (months)	Radiologic	Histological and Biochemical	Other Clinical Outcomes
105	Spine fusion (Thoraco-lumbar)	*ND*	PEMF vs CMF	PMF – 1.5 Hz (pulse); 3.8 kHz (burst) CMF – 76.6 Hz	PEMF – 25.6 pulse burst duration; 670 ms burst period	PEMF – 0.68 mT;<br><br>CMF – 40.0 ± 8.0 μT (AC) + 20.0 ± 2.0 μT (DC)	PEMF – min. 2; CMF – 0.5	12 (avg time to heal lower in CMF<PEMF<NS)	Solid fusion achieved in 11/16 (68.8%) PEMF, 21/24 (87.5%) CMF, and 20/20 (100.0%) NS patients. Stable non-union in 2/24 (8.3%) CMF, and zero PEMF and NS patients. There were 5/16 (31.3%) PEMF, 1/24 (4.2%) CMF, and zero NS patients with radiologic pseudarthrosis.	*NA*	The addition of these bone growth stimulators does not improve fusion outcomes, although CMF appears superior to PEMF.
106	Spine fusion (Thoraco-lumbar)	Patients with at least 1 of the following risk factors: prior failed fusion, multilevel fusion, nicotine use, osteoporosis, or diabetes. PEMF treatment initiated within 14 days of surgery.	PEMF (SpinalStim, Orthofix US LLC); rectangular pulse	1.5 (pulse); 3850 (burst)	98 pulses of 0,26 ms	0.4	2	6	Of the 142 patients evaluated at the 12-month follow-up, 88.0% were graded as fused, with no risk factor being significantly associated with a difference in the number of patients who had successful fusion vs those who had failed. Fusion success was 88.5% with a single risk factor, 87.5% ≥2 risk factors, and 82.3% ≥3 risk factors.	*NA*	Significant improvements in patient-reported outcomes' scores that measure disability, function, pain, quality of life, and overall well-being. From the reported adverse effects, 1.1% were related to the use of the PEMF device and included: pain from screws, increase in diarrhea, postoperative ankle dorsiflexion weakness, increased pain when wearing device, mild low back pain with device use, and increased back pain with some radicular pain through hips and groin.
107	Non-union (cervical spinal fusion) (Abstract only)	(Case report)	PEMF	*ND*	*ND*	*ND*	3	10	X-rays obtained after 15 weeks of stimulation showed improvement in bone fusion, and X-rays obtained at 31 weeks after stimulation showed even bone density around the C7 screws. PEMF has clinical potential in healing non-union of anterior cervical spine fusion.	*NA*	*NA*

Ref	Condition	Procedure	Stimuli characteristics						Outcomes		
			Type of stimulation<br><br>Stimulation device<br><br>Waveform	Frequency (Hz)	Periodicity	MF strength (mT)	Exposure time (h)	Assay duration (months)	Radiologic	Histological and Biochemical	Other Clinical Outcomes
108	Osteoporosis (post-menopausal women)	Daily calcium and vitamin D supplementation	PEMF (XT-2000B); bursts of asymmetric pulses	8 (pulse)	0.2 ms pulse bursts	3.82	0.7 (3x a week)	6	PEMF stimulation increased lumbar spine and hip BMD, and significantly reduced bone marrow fat fraction. There were no significant improvements in the control group after 6 months.	In the PEMF group, there was an increase in serum bone-specific alkaline phosphatase, and decreased C-terminal telopeptides of type 1 collagen. There were no significant improvements in the control group after 6 months.	*NA*
109	Bone Fracture (Vertebra)	Osteoporosis (post-menopausal women). Daily calcium and vitamin D supplementation	PEMF (XT-2000B); bursts of asymmetric pulses	8 (pulse)	0.2 ms pulse bursts	3.82	*ND*	3	PEMF can significantly improve the bone microstructure of the radius and tibia 3 months after vertebral fractures. Changes in hip bone density were not significant.	*NA*	PEMF treatment can significantly improve health-related quality of life scores, back pain, and body functions.
110	Osteoporosis (post-menopausal women)	Calcium dosages at 1 g/day during the entire study	PEMF; Quasi-rectangular pulse followed by quasi-triangular pulse	72	380 µs quasi-rectangular pulse followed by 6 ms quasi-triangular pulse	2.85	10	3	Bone mineral densities increased significantly during PEMF treatment and decreased the following 36 weeks after stopping the treatment. Similar but weaker response in the opposite arm, suggesting a “cross-talk” effect from either possible arm proximity during sleep or very weak general field effects.	*NA*	*NA*

Ref	Condition	Procedure	Stimuli characteristics						Outcomes		
			Type of stimulation<br><br>Stimulation device<br><br>Waveform	Frequency (Hz)	Periodicity	MF strength (mT)	Exposure time (h)	Assay duration (months)	Radiologic	Histological and Biochemical	Other Clinical Outcomes
111	Osteoporosis (post-menopausal women)	BMD T-score -2.5 or less at lumbar spine and/or femoral neck, no active treatment for osteoporosis (exception of calcium and vitamin D supplements)	PEMF	16, 18, 20, 22 (rotating every 4 min)	*ND*	3.0, 3.2, 3.4, 3.6 (rotating every 4 min)	0.8	2	*NA*	PEMF group had some significant changes in serum markers related to the Wnt/β-Catenin and the RANKL/OPG signaling pathways, which can explain some of the effects of PEMF stimulation on bone tissue metabolism. There is evidence of favourable effects of PEMF on bone tissue, and it is a safe alternative that can be an effective treatment option for osteoporosis with further research.	*NA*
112	Limb lengthening (bone formation and disuse osteoporosis)	Limbs lengthened at a rate of 1 mm/day with external fixator	PEMF (Electro Biology Inc); Quasi-rectangular pulses followed by a sharper reverse form	15 (pulse)<br>4 444 (burst)	Pulse bursts of 20 pulses with 200 μs and 25 μs pause	*ND*	4	18	Stimulation with PEMF had no effect on the new bone formation in the distraction gap but prevented bone loss adjacent to the distraction gap and increased bone density.	*NA*	*NA*
113	Implant	Hip prosthesis	PEMF (Biostim, IGEA Srl)	75	1,3 ms pulse duration	2.0 ± 0.2	6	3	In Gruen zones 5 and 6, there were 6 responders to treatment (40%) in both areas in the control group, while in the PEMF group there were 14 (93%) and 10 (66%) responders, respectively.<br>No significant differences in the average BMD values.	*NA*	Subject improvement according to Merle D'Aubigné (pain while walking) scale was higher in subjects undergoing active stimulation.
114	Implant	Dental implant with Magdent healing caps (MED)	PEMF (Magdent Ltd, Tel Aviv, Israel)	*ND*	*ND*	*ND*	24	1.8	Maxillary implants stability was significantly higher with MED healing caps compared with controls at 15, 30 and 50 days post-implantation.	*NA*	*NA*

Ref	Condition	Procedure	Stimuli characteristics						Outcomes		
			Type of stimulation<br>Stimulation device<br>Waveform	Frequency (Hz)	Periodicity	MF strength (mT)	Exposure time (h)	Assay duration (months)	Radiologic	Histological and Biochemical	Other Clinical Outcomes
115	Osteoarthritis	*ND*	PEMF (MAGCELL® ARTHRO; PHYSIOMED AG)	8	*ND*	105	0.08*2	0.5	*NA*	*NA*	PEMF stimulated group had highly significant reduction in pain, stiffness and disability in daily activities according to the Western Ontario and McMaster Universities Osteoarthritis Index (WOMAC) scales. Further, in patient assessments of the "effectiveness", it was rated by 29.5% of the participants as very good and 27.3% as good, compared to 0.0% and 15.4% in controls.
116	Osteoarthritis	Single-blind randomized control study with patients with knee OA, assigned to receive 24 sessions of either progressive resistance exercise (PRE) only (control group) or combined PEMF and PRE (treatment group)	PEMF (Pagani magnetic therapy table (PMT), Electronica Pagani, Italy)	50	90 s interval	5	0.5	2	*NA*	*NA*	No significant difference between groups in Knee Injury and Osteoarthritis Outcome Score (KOOS), Numeric Pain Rating Scale (NPRS); walking speed and 5-times chair stand test assessed at pre-treatment, post-treatment (2 months), and at follow-up 3 and 6-months. However, both treatments were equally effective in decreasing pain and improving physical function in patients with knee OA.
117	Soft Tissue Healing	Tooth extraction	HF-PEMF	1000 (pulse); $27x10^6$ (burst)	1000 pulses/s; 100 ns burst width	*ND*	24	7 days	*NA*	*NA*	Improved soft tissue healing. PEMF may be a useful adjunct for pain management after oral surgery, with significantly fewer cases of dehiscence than placebo patients.

Supplementary Table 4 – Biological outcomes quantified during IC stimulation studies in human patients suffering from musculoskeletal disorders or submitted to orthopedic surgeries. Data was collected from 40 studies and includes the evaluated musculoskeletal disorder/condition, applied complementary procedures, and quantitative outcomes (radiologic, histological or biochemical, and biomechanical) measured in each study. Each measured outcome is presented as control vs stimulation (when available), at each recorded timepoint (indicated in bold), followed by the gain/loss percentage. AC – alternating voltage; CV – constant voltage; BMD – bone mineral density; BV/TV – bone volume per total volume ration; Tb.Th – Trabecular thickness; Tb.N – Trabecular number; Tb.S – Trabecular separation; ALP – Alkaline phosphatase; HA – hydroxyapatite; PEMF – Pulsed Electromagnetic Field; CMF – Combined Magnetic Field; ND – not defined; NA – not applicable (not performed by the authors).

Ref.	Condition	Procedure	Outcomes Quantification: control vs stimulation (% of increase or decrease) at each recorded timepoint		
			Radiographic	Histological and Biochemical	Other Clinical Outcomes
74	Non-union	All patients treated with CMF until union	44.8% diagnosed non-unions healed	*NA*	*NA*
75	Non-union	All patients treated with CMF until union	75.1% diagnosed non-unions healed	*NA*	*NA*
76	Non-union	All patients treated with CMF until union	84% diagnosed non-unions healed	*NA*	*NA*
77	Non-union (scaphoid)	Thumb spica cast immobilization; PEMF treatment until healing	80% diagnosed non-unions healed	*NA*	Grip: 83% of normal levels;<br>Wrist extension: 85.3% of normal levels;<br>Wrist flexion: 91.3% of normal levels;<br>Wrist radial deviation: 85.0% of normal levels;<br>Wrist ulnar deviation: 91.1% of normal levels;<br>Average motion: 88.6% of normal levels
78	Non-union	Fixation or immobilization applied when required; treatment until fracture union	**Healing success rate (%):**<br>Long-bones: 76%<br>Non-long bones: 79%	*NA*	*NA*
79	Delayed union (long bone)	Treatment until fracture union	Healing success rate:<br>48.1% vs 77.4% (+1.61-fold)	*NA*	*NA*

Ref.	Condition	Procedure	Outcomes Quantification: control vs stimulation (% of increase or decrease) at each recorded timepoint		
			**Radiographic**	**Histological and Biochemical**	**Other Clinical Outcomes**
80	Non-union (scaphoid)	Thumb spica cast immobilization; PEMF treatment until healing	69.0% diagnosed non-unions healed	*NA*	Grip: 77% of normal levels;<br>Wrist extension: 78% of normal levels;<br>Wrist flexion: 90% of normal levels;<br>Wrist radial deviation: 79% of normal levels;<br>Wrist ulnar deviation: 72% of normal levels;<br>Average motion: 79.8% of normal levels
81	Non-union	*ND*	71.7% diagnosed non unions healed	*NA*	*NA*
82	Non-union	PEMF + anterior iliac crest bone marrow injection or autologous iliac crest bone graft	68% diagnosed non unions healed	*NA*	*NA*
83	Non-union	*ND*	89.6% diagnosed non unions healed<br>Time to heal: -40 to -60% for > 9 h/day exposures	*NA*	*NA*
84	Non-union (metatarsal)	Treatment until union	Average time to heal (weeks):<br>14.7 vs 8.9 (-39.5%)	**Protein concentration (pg/mL):**<br>BMP-5: 115 vs 200 (+73.9%);<br>BMP-7: 670 vs 1930 (188.1%);<br>EGF R: 355 vs 705 (+98.6%);<br>VEGF R2: 10 vs 55 (+5.5-fold);<br>PIGF: 25 vs 80 (+3.2-fold)<br>*(Data from BDNF not obtainable)*	*NA*
85	Non-union (tibia)	Long-leg plaster cast immobilization	Healing success rate:<br>13.6% vs 45.0% (+3.75-fold)	*NA*	*NA*
86	Delayed & Non-union (metatarsi)	Non-weightbearing cast, short leg weightbearing cast, or weightbearing postoperative shoe; PEMF treatment until healing	Average time to heal (months):<br>4.5 vs 3.0 (-33.3%)	*NA*	*NA*

Ref.	Condition	Procedure	Outcomes Quantification: control vs stimulation (% of increase or decrease) at each recorded timepoint		
			**Radiographic**	**Histological and Biochemical**	**Other Clinical Outcomes**
87	Non-union (Long bone; tibia)	Application of a Denham external fixator; PEMF treatment until healing	86.7% diagnosed non-unions healed	*NA*	*NA*
88	Non-union (Long bones*) (abstract only)*	*ND*	84.6% diagnosed non-unions healed	*NA*	*NA*
89	Congenital pseudarthrosis (tibia)	Intramedullary nailing	Healing success rate (%): 83 vs 91 (+9.6%)<br>Average time to heal (months):<br>4.9 vs 3.3 (-32.7%)	*NA*	*NA*
90	Congenital pseudarthrosis (tibia)	Immobilization during the first 6 months	54% healed lesions	*NA*	*NA*
91	Congenital pseudarthrosis (Tibia)	Boyd's dual onlay grafts and PEMF (*Case report; 17-year follow-up*)	*NA*	*NA*	*NA*
92	Delayed union	Delayed unions after foot and ankle arthrodesis treated with immobilization, limited weightbearing, and PEMF stimulation	26% diagnosed delayed unions healed;<br>Nr of patients to have satisfactory healing (revision surgery + PEMF vs PEMF): 1.3 vs 3.84 (+195.4%)	*NA*	*NA*
93	Non-union (scaphoid)	Scaphoid non-union treated by retrograde percutaneous screw fixation and PEMF	Healing success rate: 77.7% vs 66.6% (0.86-fold)	*NA*	*NA*

Ref.	Condition	Procedure	Outcomes Quantification: control vs stimulation (% of increase or decrease) at each recorded timepoint		
			Radiographic	Histological and Biochemical	Other Clinical Outcomes
94	Bone Fracture (Mandibular)	Maxillo-mandibular fixation (MMF)	**At weeks 0, 2 and 4:**<br>BMD: 145.6 vs 130.6 (-10.3%), 128.4 vs 124.1 (-3.3%), 131.0 vs 144.6 (+10.4%)<br>**BMD change:**<br>Baseline to Week 2: -17.2 vs -6.5 (-62.2%)<br>Week 2 to Week 4: +2.6 vs +20.5 (+7.9-fold)<br>Baseline to Week 4: -14.6 vs +14.0 (+195.9%)	*NA*	**At day 0, 1, 7 and 14:**<br>Pain scale score: 7.5 vs 6.7 (-10.7%); 5.6 vs 2.8 (-50.0%); 3.9 vs 1.8 (-53.8%); 2.4 vs 0.8 (-66.7%)<br>**Pain reduction from baseline to day 14:**<br>68.0% vs 87.9% (+1.29-fold)
95	Bone Fracture (Mandibular)	Maxillo-mandibular fixation (MMF)	**At days 0, 15, 30:**<br>Mean bone density: 124.5 vs 138.5 (+11.2%), 115.7 vs 135.4 (+17.0%), 118.5 vs 147.3 (+24.3%)	*NA*	*NA*
96	Bone Fracture (Mandibular)	Maxillo-mandibular fixation (MMF)	**At weeks 0, 2 and 4:**<br>BMD: 162.5 vs 159.9 (-1.6%); 117.17 vs 152.32 (+30.0%), 133.71 vs 154.59 (+15.6%)<br>**BMD change:**<br>Baseline to Week 2: -27.89 vs -4.74 (-83.0%)<br>Week 2 to Week 4: +14.12 vs +1.49 (-89.4%)<br>Baseline to Week 4: -17.71 vs -3.32 (-81.3%)	*NA*	*NA*
97	Bone Fracture (Scaphoid)	Forearm cast immobilization	Median time to heal (months): 9 vs 9 (+0.0%)	*NA*	Wrist movement: 80% vs 79% (-1.3%)
98	Bone Fracture (Scaphoid)	Forearm cast immobilization	Median time to heal (months): 6 vs 6 (+0.0%)	*NA*	Wrist movement: 87% vs 91% (+4.6%)
99	Bone fracture (tibia)	*ND*	Healing success rate:<br>71% vs 66% (0.93-fold)	*NA*	Compliant patients needing secondary surgery to union: 11.5% vs 12.5% (+1.09-fold)
100	Bone Healing	High Tibial Osteotomy	Consolidation rate (%/week): 4.18% vs 4.64% (+1.11-fold)	Serum ALP increase (µg/L; in 1 month): +0.7 (from 18.4 to 19.1) vs +3.4 (from 17.9 to 21.3) (+4.86-fold)	*NA*

Ref.	Condition	Procedure	Outcomes Quantification: control vs stimulation (% of increase or decrease) at each recorded timepoint		
			Radiographic	Histological and Biochemical	Other Clinical Outcomes
101	Bone Fracture (distal radius)	Prospective, double-blind, randomized, and sham-controlled study using PEMF applied at the fracture site immediately after open reduction and internal fixation (ORIF) surgery.	**At week 4:**<br>Union Extension (%): 54 vs 70 (+ 29.6%)	*NA*	**At weeks 4 and 6:**<br>Hand Grip (kg): 5 vs 9 (+80.0%); 7 vs 16 (+128.6%/2.3-fold increase).<br><br>**At weeks 2, 4, 6, 12 and 24:**<br><br>**Patient-rated wrist evaluation (PRWE, #):**<br>Pain: 18.0 vs 17.6 (-2.2%), 21.2 vs 22.0 (+3.8%), 23.0 vs 21.0 (-8.7%), 19.0 vs 18.0 (-5.3%), 15.0 vs 16.4 (+9.3%).<br>Function**:** 44.8 vs 44.8 (0%), 39.2 vs 38.4 (-2.0%), 35.5 vs 27.2 (-23.4%), 23.0 vs 18.6 (-19.1%), 6.8 vs 12.4 (+82.4%)
102	Spine fusion (cervical; non-union)	Anterior cervical discectomy and fusion (ACDF)	**At months 6 and 12:**<br>Fusion rate: 68.6% vs 83.6% (+1.22-fold), 86.7% vs 92.8% (+1.07-fold)	*NA*	Visual analog scale (pain): +4.3%<br>Neck disability index: +17.4%<br>SF-12 (physical health): -5.4%
103	Spine fusion (lumbar)	*ND*	Fusion rate: 65% vs 92% (+1.42-fold)	*NA*	*NA*
104	Spine fusion (lumbar/sacral) *(Abstract only)*	One-level or two-level fusions (between L3 and S1) without instrumentation	Fusion rate: 43% vs 64% (+1.49-fold)	*NA*	*NA*
105	Spine fusion (Thoraco-lumbar)	PEMF vs CMF	Fusion rate:<br>**PEMF:** 100% vs 68.8% (0.69-fold)<br>**CMF:** 100% vs 87.5% (0.88-fold)	*NA*	*NA*

Ref.	Condition	Procedure	Outcomes Quantification: control vs stimulation (% of increase or decrease) at each recorded timepoint		
			Radiographic	Histological and Biochemical	Other Clinical Outcomes
106	Spine fusion (Thoraco-lumbar)	Patients with at least 1 of the following risk factors: prior failed fusion, multilevel fusion, nicotine use, osteoporosis, or diabetes. PEMF treatment initiated within 14 days of surgery.	88% successful fusions (n=125/144)	*NA*	Short Form (SF-36) Quality of Life Survey (mental and physical scores): 47.5 vs 51.0 (+7.4%), 31.1 vs 43.0 (+38.3%);<br><br>Visual Analog Scale (back pain and leg pain): 50.1 vs 22.9 (-54.3%), 25.3 vs 16.7 (-34.0%); EuroQol 5 Dimension (EQ-5D): 0.59 vs 0.76 (+28.8%);<br><br>Oswestry Disability Index (ODI): 48.5 vs 26.6 (-45.2%)
107	Non-union (cervical spinal fusion) *(Abstract only)*	(Case report)	*NA*	*NA*	*NA*
108	Osteoporosis (post-menopausal women)	Daily calcium and vitamin D supplementation	Vertebral BMD (% change from baseline): +2.04%<br>Femoral neck BMD (% change from baseline): +1.46%<br>Total hip BMD (% change from baseline): +1.57%<br>Fat fraction (from MRI) (% change from baseline): -4.81%	Serum bone-specific ALP: +3.23%<br>Serum CTX-I: -9.12%	*NA*
109	Bone Fracture (Vertebra)	Osteoporosis (post-menopausal women). Daily calcium and vitamin D supplementation	**At month 3:**<br>Radiographic fusion rate: 81.61% vs 86.91% (+1.06-fold)<br>Total hip BMD (g/cm^2): 0.61 vs 0.73 (+19.7%);<br>BMD (radius/tibia) (g/cm^3): 193 vs 203 (+5.2%); 153 vs 173 (+13.1%);<br>BV/TV (radius/tibia) (%): 7.0 vs 7.3 (+4.3%); 9.0 vs 9.9 (+10%);<br>Ct.Th (radius/tibia) (mm): 0.40 vs 0.54 (+35.0%); 0.48 vs 0.55 (+14.6%)<br>Tb.Th (radius/tibia) (μm): 63 vs 75 (+19.0%); 63 vs 75 (+19.0%)<br>Tb.N (radius/tibia) (1/mm): 1.31 vs 1.45 (+10.7%); 1.25 vs 1.45 (+16.0%)	*NA*	**At month 3:**<br>Lumbar back pain VAS: 3.91 vs 3.05 (-22.0%);<br>ECOS-16: 2.42 vs 2.12 (-12.4%);<br>Psysical function score: 1.86 vs 1.41 (-24.2%);<br>Psychosocial score: 2.22 vs 1.36 (-38.7);<br>EuroQoL VAS: 69.4 vs 82.6 (+19.0%);<br>6-MWT: 395.2 vs 446.4 (+13.0%);<br>Chair Sit-and-Reach (left/right): 89.6 vs 96.7 (+7.9%); 88.7 vs 98.6 (+11.2%)
110	Osteoporosis (post-menopausal women)	Calcium dosages at 1 g/day during the entire study	*NA*	*NA*	*NA*

Ref.	Condition	Procedure	Outcomes Quantification: control vs stimulation (% of increase or decrease) at each recorded timepoint		
			Radiographic	Histological and Biochemical	Other Clinical Outcomes
111	Osteoporosis (post-menopausal women)	BMD T-score -2.5 or less at lumbar spine and/or femoral neck, no active treatment for osteoporosis (exception of calcium and vitamine D supplements)	*NA*	**At days 30 and 60:**<br>Sclerostin (pmol/L): 23.9 vs 21.2 (-11.3%), 26.3 vs 26.8 (+1.9%)<br>DKK-1 (pmol/L): 31.2 vs 28.0 (-10.3%), 34.1 vs 27.5 (-19.3%)<br>B-Catenin (pmol/L): 23.8 vs 34.5 (+44.8%), 21.9 vs 39.7 (+81.3%)<br>RANKL (pmol/L): 3.71 vs 2.54 (-31.5%), 3.98 vs 2.14 (-46.2%)<br>OPG (pmol/L): 8.3 vs 8.3 (+0.0%), 7.9 vs 8.7 (+10.1%)<br>RANKL/OPG: 0.47 vs 0.51 (+8.5%), 0.52 vs 0.24 (-53.8%)<br>Serum ALP (ng/mL): 15.2 vs 15.9 (+4.6%), 15.1 vs 15.7 (+4.0%)<br>Serum CTX (ng/mL): 0.39 vs 0.39 (+0.0%), 0.35 vs 0.30 (-14.3%)<br>Serum Creatinine (ng/mL): 0.82 vs 0.82 (+0.0%), 0.81 vs 0.80 (-1.2%)<br>Serum Ca^{2+} (mg/dL): 9.13 vs 9.01 (-1.3%), 9.11 vs 9.18 (+0.8%)	*NA*
112	Limb lengthening (bone formation and disuse osteoporosis)	Limb lengthening at a rate of 1 mm/day with external fixator	Distraction length (cm): 7.0 vs 8.1 (+15.7%); Lengthening index (days/cm): 39 vs 44 (+12.8%)<br>**At gap, proximal segment and distal segment:**<br>BMD (compared to baseline): 95% vs 106% (+1.12-fold), 100% vs 118% (+1.18-fold), 54% vs 87% (+1.61-fold)	*NA*	*NA*
113	Implant	Hip prosthesis	**In Gruen zones 5 and 6:**<br>Responders (people with an increase of BMD of at least 3.5%): 40% vs 93% (+2.32-fold), 40% vs 66% (+1.65-fold)	*NA*	Merle D'Aubigne´ scores (for pain, walk and mobility, > better): 1.5 vs 2.5 (+66.7%)
114	Implant	Dental implant with Magdent healing caps (MED)	**At days 15, 30, 50:**<br>Maxillary implants stability: 62.1 vs 66.2 (+6.6%), 65 vs 74 (+13.8%), 68.5 vs 75.4 (+10.1%)	*NA*	*NA*

Ref.	Condition	Procedure	Outcomes Quantification: control vs stimulation (% of increase or decrease) at each recorded timepoint		
			Radiographic	Histological and Biochemical	Other Clinical Outcomes
115	Osteoarthritis	*ND*	*NA*	*NA*	WOMAC score (lower=better):<br>56.2 vs 42.9 (-23.7%)
116	Osteoarthritis	Single-blind randomized control study with patients with knee OA, assigned to receive 24 sessions of either progressive resistance exercise (PRE) only (control group) or combined PEMF and PRE (treatment group)	*NA*	*NA*	**At pre- and post-treatment, and 3- and 6-months follow up:**<br><br>**Knee injury and Osteoarthritis Outcome Score (KOOS) (#):**<br>Total: 43.5 vs 46.4 (+6.7%); 80.5 vs 76.2 (-5.3%); 73.1 vs 74.1 (+1.4%); 73.3 vs 69.4 (-5.3%)<br>Symptoms: 54.5 vs 54.9 (+0.7%); 84.6 vs 81.0 (-4.3%); 73.9 vs 78.3 (+6.0%); 73.7 vs 74.9 (+1.6%)<br>Pain: 46.5 vs 48.3 (+3.9%); 84.5 vs 80.4 (-4.9%); 76.4 vs 74.8 (-2.1%); 76.2 vs 73.4 (-3.7%)<br>Daily Activity: 42.6 vs 48.6 (+14.1%); 86.8 vs 82.2 (-5.3%); 78.6 vs 80.1 (+1.9%); 78.3 vs 73.3 (-6.4%)<br>Sport: 17.8 vs 22.2 (+24.7%); 69.7 vs 70.3 (+0.9%); 53.4 vs 55.0 (+3.0%); 48.4 vs 48.1 (-0.6%)<br>Quality of Life: 30.5 vs 30.1 (-1.3%); 66.0 vs 61.3 (-7.1%); 62.1 vs 62.5 (+0.6%); 57.2 vs 63.2 (+10.5%)<br><br>4-m walking test (m/s): 1.18 vs 1.08 (-8.5); 1.82 vs 1.67 (-8.2%); 1.67 vs 1.67 (0.0%); 1.60 vs 1.74 (+8.7%)<br>Chair stand test (s): 13.9 vs 16.4 (+18.0%); 9.9 vs 10.8 (+9.1%); 10.0 vs 9.3 (-7.0%); 8.8 vs 9.0 (+2.3%)<br>Numeric pain rating scale (NPRS): 6.8 vs 6.6 (-2.9%); 2.8 vs 3.1 (10.7%); 3.4 vs 3.0 (-11.8%); 2.9 vs 3.3 (+13.8)
117	Soft Tissue Healing	Tooth extraction	*NA*	*NA*	**Control & placebo vs stimulation:**<br>Pain scale (VAS) 7-day cumulative score:<br>198 vs 196 vs 144 (-26.9%)<br>Dehiscence:<br>17 vs 28 vs 14 (-37.8%)

References of Supplementary Tables

1. Márquez-Gamiño S, Sotelo F, Sosa M, et al. Pulsed electromagnetic fields induced femoral metaphyseal bone thickness changes in the rat. *Bioelectromagnetics*. 2008;29(5):406-409. doi:10.1002/bem.20396

2. Wang Y, Pu X, Shi W, et al. Pulsed electromagnetic fields promote bone formation by activating the sAC–cAMP–PKA–CREB signaling pathway. *Journal Cellular Physiology*. 2019;234(3):2807-2821. doi:10.1002/jcp.27098

3. Aaron RK, Ciombor DMcK, Jolly G. Stimulation of experimental endochondral ossification by low-energy pulsing electromagnetic fields. *Journal of Bone and Mineral Research*. 1989;4(2):227-233. doi:10.1002/jbmr.5650040215

4. Takano-Yamamoto T, Kawakami M, Sakuda M. Effect of a Pulsing Electromagnetic Field on Demineralized Bone-matrix-induced Bone Formation in a Bony Defect in the Premaxilla of Rats: *http://dx.doi.org/101177/00220345920710121301*. 1992;71(12):1920-1925. doi:10.1177/00220345920710121301

5. Liu Y, Hao L, Jiang L, Li H. Therapeutic effect of pulsed electromagnetic field on bone wound healing in rats. *Electromagnetic Biology and Medicine*. 2021;40(1):26-32. doi:10.1080/15368378.2020.1851252

6. Li Y, Pan Q, Zhang N, et al. A novel pulsed electromagnetic field promotes distraction osteogenesis via enhancing osteogenesis and angiogenesis in a rat model. *Journal of Orthopaedic Translation*. 2020;25:87-95. doi:10.1016/j.jot.2020.10.007

7. Bilgin HM, Çelik F, Gem M, et al. Effects of local vibration and pulsed electromagnetic field on bone fracture: A comparative study. *Bioelectromagnetics*. 2017;38(5):339-348. doi:10.1002/BEM.22043

8. Atalay Y, Gunes N, Guner MD, Akpolat V, Celik MS, Guner R. Pentoxifylline and electromagnetic field improved bone fracture healing in rats. *Drug Des Devel Ther*. 2015;9:5195-5201. doi:10.2147/DDDT.S89669

9. Yang HJ, Kim RY, Hwang SJ. Pulsed Electromagnetic Fields Enhance Bone Morphogenetic Protein-2 Dependent-Bone Regeneration. *Tissue Eng Part A*. 2015;21(19-20):2629-2637. doi:10.1089/ten.TEA.2015.0032

10. Oltean-Dan D, Dogaru GB, Apostu D, et al. Enhancement of bone consolidation using high-frequency pulsed electromagnetic fields (HF-PEMFs): An experimental study on rats. *Bosnian Journal of Basic Medical Sciences*. 2019;19(2):201. doi:10.17305/BJBMS.2019.3854

11. Ibiwoye MO, Powell KA, Grabiner MD, et al. Bone mass is preserved in a critical-sized osteotomy by low energy pulsed electromagnetic fields as quantitated by in vivo micro-computed tomography. *Journal of Orthopaedic Research*. 2004;22(5):1086-1093. doi:10.1016/J.ORTHRES.2003.12.017

12. Midura RJ, Ibiwoye MO, Powell KA, et al. Pulsed electromagnetic field treatments enhance the healing of fibular osteotomies. *Journal of Orthopaedic Research*. 2005;23(5):1035-1046. doi:10.1016/J.ORTHRES.2005.03.015

13. Leisner S, Shahar R, Aizenberg I, Lichovsky D, Levin-Harrus T. The Effect of Short-duration, High-intensity Electromagnetic Pulses on Fresh Ulnar Fractures in Rats. *Journal of Veterinary Medicine Series A*. 2002;49(1):33-37. doi:10.1046/J.1439-0442.2002.00386.X

14. Guizzardi S, Silvestre M Di, Govoni P, Scandroglio R. Pulsed electromagnetic field stimulation on posterior spinal fusions: A histological study in rats. *Journal of Spinal Disorders*. 1994;7(1):36-40. doi:10.1097/00002517-199407010-00005

15. Tucker JJ, Cirone JM, Morris TR, et al. Pulsed electromagnetic field therapy improves tendon-to-bone healing in a rat rotator cuff repair model. *Journal of orthopaedic research : official publication of the Orthopaedic Research Society*. 2017;35(4):902. doi:10.1002/JOR.23333

16. Huegel J, Choi DS, Nuss CA, et al. Effects of pulsed electromagnetic field therapy at different frequencies and durations on rotator cuff tendon-to-bone healing in a rat model. *Journal of shoulder and elbow surgery*. 2018;27(3):553. doi:10.1016/J.JSE.2017.09.024

17. Huegel J, Boorman-Padgett JF, Nuss CA, et al. Effects of Pulsed Electromagnetic Field Therapy on Rat Achilles Tendon Healing. *Journal of orthopaedic research : official publication of the Orthopaedic Research Society*. 2020;38(1):70. doi:10.1002/JOR.24487

18. Zhou J, Liao Y, Xie H, et al. Effects of combined treatment with ibandronate and pulsed electromagnetic field on ovariectomy-induced osteoporosis in rats. *Bioelectromagnetics*. 2017;38(1):31-40. doi:10.1002/BEM.22012

19. Zhou J, Liao Y, Zeng Y, Xie H, Fu C, Li N. Effect of intervention initiation timing of pulsed electromagnetic field on ovariectomy-induced osteoporosis in rats. *Bioelectromagnetics*. 2017;38(6):456-465. doi:10.1002/BEM.22059

20. van der Jagt OP, van der Linden JC, Waarsing JH, Verhaar JAN, Weinans H. Systemic treatment with pulsed electromagnetic fields do not affect bone microarchitecture in osteoporotic rats. *International Orthopaedics (SICOT)*. 2012;36(7):1501-1506. doi:10.1007/s00264-011-1471-8

21. Androjna C, Fort B, Zborowski M, Midura RJ. Pulsed electromagnetic field treatment enhances healing callus biomechanical properties in an animal model of osteoporotic fracture. *Bioelectromagnetics*. 2014;35(6):396-405. doi:10.1002/BEM.21855

22. Bernardo DV, Ferreira CL, Nunes CMM, et al. Effects of the Pulsed Electromagnetic Fields on Experimental Periodontitis and Estrogen Deficiency. *Bioelectromagnetics*. 2022;43(7):426-437. doi:10.1002/bem.22424

23. Jing D, Li F, Jiang M, et al. Pulsed Electromagnetic Fields Improve Bone Microstructure and Strength in Ovariectomized Rats through a Wnt/Lrp5/β-Catenin Signaling-Associated Mechanism. *PLoS One*. 2013;8(11):e79377. doi:10.1371/journal.pone.0079377

24. Wang Q, Zhou J, Wang X, et al. Coupling induction of osteogenesis and type H vessels by pulsed electromagnetic fields in ovariectomy-induced osteoporosis in mice. *Bone*. 2022;154:116211. doi:10.1016/j.bone.2021.116211

25. Androjna C, Yee CS, White CR, et al. A comparison of alendronate to varying magnitude PEMF in mitigating bone loss and altering bone remodeling in skeletally mature osteoporotic rats. *Bone*. 2021;143:115761. doi:10.1016/j.bone.2020.115761

26. Li B, Bi J, Li W, et al. Effects of pulsed electromagnetic fields on histomorphometry and osteocalcin in disuse osteoporosis rats. *Technology and Health Care*. 2017;25(S1):13-20. doi:10.3233/THC-171301

27. Jing D, Cai J, Wu Y, et al. Pulsed Electromagnetic Fields Partially Preserve Bone Mass, Microarchitecture, and Strength by Promoting Bone Formation in Hindlimb-Suspended Rats: PEMF PARTIALLY PROMOTE BONE FORMATION AND BONE QUALITY IN DISUSE RATS. *J Bone Miner Res*. 2014;29(10):2250-2261. doi:10.1002/jbmr.2260

28. Shen WW, Zhao JH. Pulsed electromagnetic fields stimulation affects BMD and local factor production of rats with disuse osteoporosis. *Bioelectromagnetics*. 2010;31(2):113-119. doi:10.1002/BEM.20535

29. Li WY, Li XY, Tian YH, et al. Pulsed electromagnetic fields prevented the decrease of bone formation in hindlimb-suspended rats by activating sAC/cAMP/PKA/CREB signaling pathway. *Bioelectromagnetics*. 2018;39(8):569-584. doi:10.1002/bem.22150

30. Jing D, Cai J, Wu Y, et al. Moderate-Intensity Rotating Magnetic Fields Do Not Affect Bone Quality and Bone Remodeling in Hindlimb Suspended Rats. *PLoS ONE*. 2014;9(7). doi:10.1371/JOURNAL.PONE.0102956

31. Topal O, Çina Aksoy M, Ciriş İM, Doğuç DK, Sert S, Çömlekçi S. Assessment of the effect of pulsed electromagnetic field application on the healing of bone defects in rats with heparin-induced osteoporosis. *Electromagnetic Biology and Medicine*. 2020;39(3):206-217. doi:10.1080/15368378.2020.1762636

32. Jiang Y, Gou H, Wang S, Zhu J, Tian S, Yu L. Effect of Pulsed Electromagnetic Field on Bone Formation and Lipid Metabolism of Glucocorticoid-Induced Osteoporosis Rats through Canonical Wnt Signaling Pathway. *Evid Based Complement Alternat Med*. 2016;2016:4927035. doi:10.1155/2016/4927035

33. Zhou J, Wang J, Qu M, et al. Effect of the Pulsed Electromagnetic Field Treatment in a Rat Model of Senile Osteoporosis In Vivo. *Bioelectromagnetics*. 2022;43(7):438-447. doi:10.1002/bem.22423

34. Nunes CMM, Ferreira CL, Bernardo DV, et al. Evaluation of pulsed electromagnetic field protocols in implant osseointegration: in vivo and in vitro study. *Clinical Oral Investigations*. 2021;25(5):2925-2937. doi:10.1007/s00784-020-03612-x

35. Grana DR, Marcos HJA, Kokubu GA. Pulsed electromagnetic fields as adjuvant therapy in bone healing and peri-implant bone formation: an experimental study in rats. *Acta odontologica latinoamericana : AOL*. 2008;21(1):77-83.

36. Veronesi F, Fini M, Sartori M, Parrilli A, Martini L, Tschon M. Pulsed electromagnetic fields and platelet rich plasma alone and combined for the treatment of wear-mediated periprosthetic osteolysis: An in vivo study. *Acta Biomaterialia*. 2018;77:106-115. doi:10.1016/j.actbio.2018.07.012

37. Akca K, Sarac E, Baysal U, Fanuscu M, Chang TL, Cehreli M. Micro-morphologic changes around biophysically-stimulated titanium implants in ovariectomized rats. *Head & Face Medicine*. 2007;3(1):28. doi:10.1186/1746-160X-3-28

38. Ma Y, Chen X, He F, et al. Low frequency pulsed electromagnetic fields exposure alleviate the abnormal subchondral bone remodeling at the early stage of temporomandibular joint osteoarthritis. *BMC Musculoskelet Disord*. 2022;23(1):987. doi:10.1186/s12891-022-05916-3

39. Ma Y, He F, Chen X, et al. Low-frequency pulsed electromagnetic fields alleviate the condylar cartilage degeneration and synovitis at the early stage of temporomandibular joint osteoarthritis. *Journal of Oral Rehabilitation*. 2023;n/a(n/a). doi:10.1111/joor.13636

40. Yang X, He H, Zhou Y, et al. Pulsed electromagnetic field at different stages of knee osteoarthritis in rats induced by low-dose monosodium iodoacetate: Effect on subchondral trabecular bone microarchitecture and cartilage degradation. *Bioelectromagnetics*. 2017;38(3):227-238. doi:10.1002/BEM.22028

41. Yang X, He H, Gao Q, He C. Pulsed electromagnetic field improves subchondral bone microstructure in knee osteoarthritis rats through a Wnt/β-catenin signaling-associated mechanism. *Bioelectromagnetics*. 2018;39(2):89-97. doi:10.1002/bem.22106

42. Fini M, Giavaresi G, Torricelli P, et al. Pulsed electromagnetic fields reduce knee osteoarthritic lesion progression in the aged Dunkin Hartley guinea pig. *J Orthop Res*. 2005;23(4):899-908. doi:10.1016/j.orthres.2005.01.008

43. Veronesi F, Torricelli P, Giavaresi G, et al. In vivo effect of two different pulsed electromagnetic field frequencies on osteoarthritis. *Journal of Orthopaedic Research*. 2014;32(5):677-685. doi:10.1002/JOR.22584

44. Fredericks DC, Piehl DJ, Baker JT, Abbott J, Nepola J V. Effects of Pulsed Electromagnetic Field Stimulation on Distraction Osteogenesis in the Rabbit Tibial Leg Lengthening Model. *Journal of Pediatric Orthopaedics*. 2003;23(4).

45. Pienkowski D, Pollack SR, Brighton CT, Griffith NJ. Low-power electromagnetic stimulation of osteotomized rabbit fibulae. A randomized, blinded study. *The Journal of bone and joint surgery American volume*. 1994;76(4):489-501. doi:10.2106/00004623-199404000-00003

46. Fredericks DC, Petersen EB, Rhodes M, Larew GA, Nepola J V. The Effect of Pulsed Electromagnetic Field and Combined Magnetic Field Exposure Time on Healing of a Rabbit Tibial Osteotomy. *The Iowa orthopaedic journal*. 2019;39(2):20-26.

47. Bao Z, Fan M, Ma L, Duan Q, Jiang W. The effects of pulsed electromagnetic fields combined with a static magnetic intramedullary implant on the repair of bone defects: A preliminary study. *Electromagnetic Biology and Medicine*. 2019;38(3):210-217. doi:10.1080/15368378.2019.1625785

48. Veronesi F, Cadossi M, Giavaresi G, et al. Pulsed electromagnetic fields combined with a collagenous scaffold and bone marrow concentrate enhance osteochondral regeneration: an in vivo study. *BMC Musculoskelet Disord*. 2015;16:233. doi:10.1186/s12891-015-0683-2

49. Matsumoto H, Ochi M, Abiko Y, Hirose Y, Kaku T, Sakaguchi K. Pulsed electromagnetic fields promote bone formation around dental implants inserted into the femur of rabbits. *Clinical Oral Implants Research*. 2000;11(4):354-360. doi:10.1034/J.1600-0501.2000.011004354.X

50. Özen J, Atay A, Oruç S, Dalkiz M, Beydemir B, Develi S. Evaluation of Pulsed Electromagnetic Fields on Bone Healing After Implant Placement in the Rabbit Mandibular Model. *Turk J Med Sci*. 2004;34(2):91-95.

51. Barak S, Neuman M, Iezzi G, Piattelli A, Perrotti V, Gabet Y. A new device for improving dental implants anchorage: A histological and micro-computed tomography study in the rabbit. *Clinical Oral Implants Research*. 2016;27(8):935-942. doi:10.1111/clr.12661

52. Yonemori K, Matsunaga S, Ishidou Y, Maeda S, Yoshida H. Early effects of electrical stimulation on osteogenesis. *Bone*. 1996;19(2):173-180. doi:10.1016/8756-3282(96)00169-x

53. Ijiri K, Matsunaga S, Fukuyama K, et al. The effect of pulsing electromagnetic field on bone ingrowth into a porous coated implant. *Anticancer Res*. 1996;16(5A):2853-2856.

54. Spadaro JA, Albanese SA, Chase SE. Electromagnetic effects on bone formation at implants in the medullary canal in rabbits. *Journal of Orthopaedic Research*. 1990;8(5):685-693. doi:10.1002/jor.1100080510

55. Jing D, Zhai M, Tong S, et al. Pulsed electromagnetic fields promote osteogenesis and osseointegration of porous titanium implants in bone defect repair through a Wnt/β-catenin signaling-associated mechanism. *Sci Rep*. 2016;6(1):32045. doi:10.1038/srep32045

56. Shimizu T, Zerwekh JE, Videman T, et al. Bone ingrowth into porous calcium phosphate ceramics: Influence of pulsing electromagnetic field. *Journal of Orthopaedic Research*. 1988;6(2):248-258. doi:10.1002/jor.1100060212

57. Ottani V, Raspanti M, Martini D, et al. Electromagnetic stimulation on the bone growth using backscattered electron imaging. *Micron*. 2002;33(2):121-125. doi:10.1016/S0968-4328(01)00008-7

58. Fini M, Cadossi R, Can& ’ V, et al. The effect of pulsed electromagnetic fields on the osteointegration of hydroxyapatite implants in cancellous bone: a morphologic and microstructural in vivo study. *Journal of Orthopaedic Research Journal of Orthopedic Research*. 2002;20:756-763. doi:10.1016/S0736-0266

59. Fini M, Giavaresi G, Giardino R, Cavani F, Cadossi R. Histomorphometric and mechanical analysis of the hydroxyapatite-bone interface after electromagnetic stimulation. *The Journal of Bone and Joint Surgery British volume*. 2006;88-B(1):123-128. doi:10.1302/0301-620X.88B1.16496

60. Buzzá EP, Shibli JA, Barbeiro RH, Barbosa JR de A. Effects of Electromagnetic Field on Bone Healing Around Commercially Pure Titanium Surface: Histologic and Mechanical Study in Rabbits. *Implant Dentistry*. 2003;12(2):182-187. doi:10.1097/01.ID.0000058385.23346.4D

61. Cai J, Li W, Sun T, Li X, Luo E, Jing D. Pulsed electromagnetic fields preserve bone architecture and mechanical properties and stimulate porous implant osseointegration by promoting bone anabolism in type 1 diabetic rabbits. *Osteoporosis International*. 2018;29(5):1177-1191. doi:10.1007/s00198-018-4392-1

62. Cai J, Shao X, Yang Q, et al. Pulsed electromagnetic fields modify the adverse effects of glucocorticoids on bone architecture, bone strength and porous implant osseointegration by rescuing bone-anabolic actions. *Bone*. 2020;133. doi:10.1016/j.bone.2020.115266

63. Xu D, Zhang T, Qu J, Hu J, Lu H. Enhanced patella-patellar tendon healing using combined magnetic fields in a rabbit model. *American Journal of Sports Medicine*. 2014;42(10):2495-2501. doi:10.1177/0363546514541539

64. Bassett CAL, Pawluk RJ, Pilla AA. Augmentation of bone repair by inductively coupled electromagnetic fields. *Science*. 1974;184(4136):575-577. doi:10.1126/science.184.4136.575

65. Inoue N, Ohnishi I, Chen D, Deitz LW, Schwardt JD, Chao EYS. Effect of pulsed electromagnetic fields (PEMF) on late-phase osteotomy gap healing in a canine tibial model. *Journal of Orthopaedic Research*. 2002;20(5):1106-1114. doi:10.1016/S0736-0266(02)00031-1

66. Ito M, Fay LA, Ito Y, Yuan MR, Edwards WT, Yuan HA. The effect of pulsed electromagnetic fields on instrumented posterolateral spinal fusion and device-related stress shielding. *Spine*. 1997;22(4):382-388. doi:10.1097/00007632-199702150-00005

67. Skerry TM, Pead MJ, Lanyon LE. Modulation of bone loss during disuse by pulsed electromagnetic fields. *Journal of Orthopaedic Research*. 1991;9(4):600-608. doi:10.1002/jor.1100090417

68. Stefani RM, Barbosa S, Tan AR, et al. Pulsed electromagnetic fields promote repair of focal articular cartilage defects with engineered osteochondral constructs. *Biotechnology and Bioengineering*. 2020;117(5):1584-1596. doi:10.1002/bit.27287

69. Kahanovitz N, Arnoczky SP, Nemzek J, Shores A. The effect of electromagnetic pulsing on posterior lumbar spinal fusions in dogs. *Spine*. 1994;19(6):705-709. doi:10.1097/00007632-199403001-00010

70. Do Nascimento C, Issa JPM, Da Silva Mello AS, De Albuquerque Junior RF. Effect of electromagnetic field on bone regeneration around dental implants after immediate placement in the dog mandible: A pilot study. *Gerodontology*. 2012;29(2). doi:10.1111/j.1741-2358.2011.00525.x

71. Enzler MA, Sumner-Smith G, Waelchli-Suter C, Perren SM. Treatment of nonuniting osteotomies with pulsating electromagnetic fields. A controlled animal experiment. *Clinical orthopaedics and related research*. 1984;(187):272-276.

72. Miller GJ, Burchardt H, Enneking WF, Tylkowski CM. Electromagnetic stimulation of canine bone grafts. *JBJS*. 1984;66(5):693-698.

73. Law H, Annan I, McCarthy I, et al. The effect of induced electric currents on bone after experimental osteotomy in sheep. *The Journal of Bone and Joint Surgery British volume*. 1985;67-B(3):463-469. doi:10.1302/0301-620X.67B3.3873459

74. Phillips M, Zoltan J, Petrisor B, Sprague S, Baumhauer J. The use of combined magnetic field treatment for fracture nonunions: A prospective observational study. *Journal of Long-Term Effects of Medical Implants*. 2016;26(3):261-270. doi:10.1615/JLongTermEffMedImplants.2016016816

75. Phillips M, Baumhauer J, Sprague S, Zoltan J. Use of combined magnetic field treatment for fracture nonunion. *Journal of Long-Term Effects of Medical Implants*. 2016;26(3):277-284. doi:10.1615/JLongTermEffMedImplants.2016016818

76. Sibanda V, Anazor F, Relwani J, Dhinsa BS. Outcomes of the Treatment of Fracture Non-union Using Combined Magnetic Field Bone Growth Stimulation: Experiences From a UK Trauma Unit. *Cureus*. Published online May 18, 2022. doi:10.7759/cureus.25100

77. Frykman GK, Taleisnik J, Peters G, et al. Treatment of nonunited scaphoid fractures by pulsed electromagnetic field and cast. *Journal of Hand Surgery*. 1986;11(3):344-349. doi:10.1016/S0363-5023(86)80140-X

78. Punt BJ, den Hoed PT, Fontijne WPJ. Pulsed electromagnetic fields in the treatment of nonunion. *European Journal of Orthopaedic Surgery & Traumatology 2007 18:2*. 2007;18(2):127-133. doi:10.1007/S00590-007-0271-8

79. Shi H fei, Xiong J, Chen Y xin, et al. Early application of pulsed electromagnetic field in the treatment of postoperative delayed union of long-bone fractures: a prospective randomized controlled study. *BMC Musculoskeletal Disorders*. 2013;14:35. doi:10.1186/1471-2474-14-35

80. Adams BD, Frykman GK, Taleisnik J. Treatment of scaphoid nonunion with casting and pulsed electromagnetic fields: A study continuation. *Journal of Hand Surgery*. 1992;17(5):910-914. doi:10.1016/0363-5023(92)90467-4

81. Sharrard WJ, Sutcliffe ML, Robson MJ, Maceachern AG. The treatment of fibrous non-union of fractures by pulsing electromagnetic stimulation. *J Bone Joint Surg Br*. 1982;64(2):189-193. doi:10.1302/0301-620X.64B2.6978339

82. Boyette MY, Herrera-Soto JA. Treatment of delayed and nonunited fractures and osteotomies with pulsed electromagnetic field in children and adolescents. *Orthopedics*. 2012;35(7). doi:10.3928/01477447-20120621-20

83. Murray HB, Pethica BA. A follow-up study of the in-practice results of pulsed electromagnetic field therapy in the management of nonunion fractures. *Orthopedic research and reviews*. 2016;8:67-72. doi:10.2147/ORR.S113756

84. Streit A, Watson BC, Granata JD, et al. Effect on Clinical Outcome and Growth Factor Synthesis with Adjunctive Use of Pulsed Electromagnetic Fields for Fifth Metatarsal Nonunion Fracture: A Double-Blind Randomized Study. *Foot and Ankle International*. 2016;37(9):919-923. doi:10.1177/1071100716652621

85. Sharrard WJW. A double-blind trial of pulsed electromagnetic fields for delayed union of tibial fractures. *Journal of Bone and Joint Surgery - Series B*. 1990;72(3):347-355. doi:10.1302/0301-620x.72b3.2187877

86. George B. Holmes Jr. Treatment of Delayed Unions and Nonunions of the Proximal Fifth Metatarsal with Pulsed Electromagnetic Fields: *http://dx.doi.org/101177/107110079401501006*. 1994;15(10):552-556. doi:10.1177/107110079401501006

87. Simonis RB, Good C, Cowell TK. The treatment of non-union by pulsed electromagnetic fields combined with a Denham external fixator. *Injury*. 1984;15(4):255-260. doi:10.1016/0020-1383(84)90010-X

88. Satter Syed A, Islam MS, Rabbani KS, Talukder MS. Pulsed electromagnetic fields for the treatment of bone fractures. *Bangladesh Medical Research Council bulletin*. 1999;25(1):6-10.

89. Cebrián JL, Gallego P, Francés A, et al. Comparative study of the use of electromagnetic fields in patients with pseudoarthrosis of tibia treated by intramedullary nailing. *International Orthopaedics*. 2010;34(3):437. doi:10.1007/S00264-009-0806-1

90. Bassett CAL, Schink-Ascani M. Long-term pulsed electromagnetic field (PEMF) results in congenital pseudarthrosis. *Calcified Tissue International*. 1991;49(3):216-220. doi:10.1007/BF02556121

91. Ito H, Shirai Y, Gembun Y. A Case of Congenital Pseudarthrosis of the Tibia Treated with Pulsing Electromagnetic Fields (17-Year Follow-up). *Journal of Nippon Medical School*. 2000;67(3):198-201. doi:10.1272/JNMS.67.198

92. Saltzman C, Lightfoot A, Amendola A. PEMF as Treatment for Delayed Healing of Foot and Ankle Arthrodesis: *http://dx.doi.org/101177/107110070402501102*. 2016;25(11):771-773. doi:10.1177/107110070402501102

93. Pereira A, Hidalgo Díaz JJ, Saur M, Salazar Botero S, Facca S, Liverneaux P. Carpal scaphoid non-union treatment: a retrospective trial comparing simple retrograde percutaneous screw fixation versus percutaneous screw fixation plus pulsed electromagnetic fields (Physiostim®). *European Journal of Orthopaedic Surgery and Traumatology*. 2017;27(4):521-525. doi:10.1007/s00590-017-1960-6

94. Mohajerani H, Tabeie F, Vossoughi F, Jafari E, Assadi M. Effect of pulsed electromagnetic field on mandibular fracture healing: A randomized control trial, (RCT). *Journal of Stomatology, Oral and Maxillofacial Surgery*. 2019;120(5):390-396. doi:10.1016/j.jormas.2019.02.022

95. Abdelrahim A, Hassanein HR, Dahaba M. Effect of Pulsed Electromagnetic Field on Healing of Mandibular Fracture: A Preliminary Clinical Study. *Journal of Oral and Maxillofacial Surgery*. 2011;69(6):1708-1717. doi:10.1016/J.JOMS.2010.10.013

96. Refai H, Radwan D, Hassanien N. Radiodensitometric Assessment of the Effect of Pulsed Electromagnetic Field Stimulation Versus Low Intensity Laser Irradiation on Mandibular Fracture Repair: A Preliminary Clinical Trial. *Journal of Maxillofacial & Oral Surgery*. 2014;13(4):451. doi:10.1007/S12663-013-0551-2

97. Hannemann PFW, Göttgens KWA, Van Wely BJ, et al. The clinical and radiological outcome of pulsed electromagnetic field treatment for acute scaphoid fractures: A randomised double-blind placebo-controlled multicentre trial. *Journal of Bone and Joint Surgery - Series B*. 2012;94 B(10):1403-1408. doi:10.1302/0301-620X.94B10.28844

98. Hannemann PFW, Van Wezenbeek MR, Kolkman KA, et al. CT scan-evaluated outcome of pulsed electromagnetic fields in the treatment of acute scaphoid fractures: A randomised, multicentre, double-blind, placebop controlled trial. *Bone and Joint Journal*. 2014;96 B(8):1070-1076. doi:10.1302/0301-620X.96B8.33767

99. Adie S, Harris IA, Naylor JM, et al. Pulsed electromagnetic field stimulation for acute tibial shaft fractures: A multicenter, double-blind, randomized trial. *Journal of Bone and Joint Surgery - Series A*. 2011;93(17):1569-1576. doi:10.2106/JBJS.J.00869

100. Ziegler P, Nussler AK, Wilbrand B, et al. Pulsed Electromagnetic Field Therapy Improves Osseous Consolidation after High Tibial Osteotomy in Elderly Patients—A Randomized, Placebo-Controlled, Double-Blind Trial. *Journal of Clinical Medicine 2019, Vol 8, Page 2008*. 2019;8(11):2008. doi:10.3390/JCM8112008

101. Factor S, Druckmann I, Kazum E, et al. A novel pulsed electromagnetic field device as an adjunct therapy to surgical treatment of distal radius fractures: a prospective, double-blind, sham-controlled, randomized pilot study. *Arch Orthop Trauma Surg*. 2024;144(1):543-550. doi:10.1007/s00402-023-05117-0

102. Foley KT, Mroz TE, Arnold PM, et al. Randomized, prospective, and controlled clinical trial of pulsed electromagnetic field stimulation for cervical fusion. *The Spine Journal*. 2008;8(3):436-442. doi:10.1016/J.SPINEE.2007.06.006

103. Mooney V. A randomized double-blind prospective study of the efficacy of pulsed electromagnetic fields for interbody lumbar fusions. *Spine*. 1990;15(7):708-712. doi:10.1097/00007632-199007000-00016

104. Linovitz RJ, Pathria M, Bernhardt M, et al. Combined magnetic fields accelerate and increase spine fusion: A double-blind, randomized, placebo controlled study. *Spine*. 2002;27(13):1383-1389. doi:10.1097/00007632-200207010-00002

105. Cheaney B, El Hashemi M, Obayashi J, Than KD. Combined magnetic field results in higher fusion rates than pulsed electromagnetic field bone stimulation after thoracolumbar fusion surgery. *Journal of Clinical Neuroscience*. 2020;74:115-119. doi:10.1016/j.jocn.2020.02.012

106. Weinstein MA, Beaumont A, Campbell P, et al. Pulsed Electromagnetic Field Stimulation in Lumbar Spine Fusion for Patients With Risk Factors for Pseudarthrosis. *International Journal of Spine Surgery*. 2023;17(6):816-823. doi:10.14444/8549

107. Mackenzie DM, Veninga FDM. Reversal of Delayed Union of Anterior Cervical Fusion Treated with Pulsed Electromagnetic Field Stimulation: Case Report. *Southern Medical Journal*. 2004;97(5):519-524.

108. Li S, Jiang H, Wang B, et al. Magnetic Resonance Spectroscopy for Evaluating the Effect of Pulsed Electromagnetic Fields on Marrow Adiposity in Postmenopausal Women With Osteopenia. *Journal of Computer Assisted Tomography*. 2018;42(5):792-797. doi:10.1097/RCT.0000000000000757

109. Liu W, Jin X, Guan Z, Zhou Q. Pulsed Electromagnetic Field Affects the Development of Postmenopausal Osteoporotic Women with Vertebral Fractures. *Biomed Res Int*. 2021;2021:4650057. doi:10.1155/2021/4650057

110. Tabrah F, Hoffmeier M, Gilbert F, Batkin S, Bassett CAL. Bone density changes in osteoporosis-prone women exposed to pulsed electromagnetic fields (PEMFs). *Journal of Bone and Mineral Research*. 1990;5(5):437-442. doi:10.1002/JBMR.5650050504

111. Catalano A, Loddo S, Bellone F, Pecora C, Lasco A, Morabito N. Pulsed electromagnetic fields modulate bone metabolism via RANKL/OPG and Wnt/β-catenin pathways in women with postmenopausal osteoporosis: A pilot study. *Bone*. 2018;116:42-46. doi:10.1016/j.bone.2018.07.010

112. Eyres KS, Saleh M, Kanis JA. Effect of pulsed electromagnetic fields on bone formation and bone loss during limb lengthening. *Bone*. 1996;18(6):505-509. doi:10.1016/8756-3282(96)00070-1

113. Dallari D, Fini M, Giavaresi G, et al. Effects of pulsed electromagnetic stimulation on patients undergoing hip revision prostheses: A randomized prospective double-blind study. *Bioelectromagnetics*. 2009;30(6):423-430. doi:10.1002/BEM.20492

114. Barak S, Matalon S, Dolkart O, Zavan B, Mortellaro C, Piattelli A. Miniaturized electromagnetic device abutment improves stability of the dental implants. *Journal of Craniofacial Surgery*. 2019;30(4):1055-1057. doi:10.1097/SCS.0000000000004763

115. Wuschech H, von Hehn U, Mikus E, Funk RH. Effects of PEMF on patients with osteoarthritis: Results of a prospective, placebo-controlled, double-blind study. *Bioelectromagnetics*. 2015;36(8):576-585. doi:10.1002/bem.21942

116. Yabroudi MA, Aldardour A, Nawasreh ZH, Obaidat SM, Altubasi IM, Bashaireh K. Effects of the combination of pulsed electromagnetic field with progressive resistance exercise on knee osteoarthritis: A randomized controlled trial. *Journal of Back and Musculoskeletal Rehabilitation*. 2024;37(1):55-65. doi:10.3233/BMR-220261

117. Stocchero M, Gobbato L, De Biagi M, Bressan E, Sivolella S. Pulsed electromagnetic fields for postoperative pain: A randomized controlled clinical trial in patients undergoing mandibular third molar extraction. *Oral Surgery, Oral Medicine, Oral Pathology and Oral Radiology*. 2015;119(3):293-300. doi:10.1016/j.oooo.2014.11.017
